# Cis-regulatory evolution shapes facial diversity in birds and mammals

**DOI:** 10.1126/sciadv.aec2511

**Published:** 2026-05-06

**Authors:** Stella Kyomen, Louk W. G. Seton, Laura E. Cook, Elio Escamilla-Vega, Andrea P. Murillo-Rincón, Alexander Jacobsen, Amor Damatac, Carsten Fortmann-Grote, Janina Fuss, Axel Visel, Markéta Kaucká

**Affiliations:** ^1^ Max Planck Institute for Evolutionary Biology, August-Thienemann-Straße 2, 24306 Plön, Germany.; ^2^ Environmental Genomics & System Biology Division, Lawrence Berkeley National Laboratory, 1 Cyclotron Road, Berkeley, CA 94720, USA.; ^3^ Zentrum für Molekulare Biowissenschaften (ZMB), Am Botanischen Garten 11, 24118 Kiel, Germany.

## Abstract

Birds and mammals exhibit extraordinary facial diversity, reflecting adaptations to distinct ecological niches and feeding strategies. While core face-building developmental programs are conserved and orchestrated by interactions between ectodermal organizers and the underlying mesenchyme, mechanisms driving facial shape variation remain poorly understood. Here, we integrate single-cell transcriptomic and chromatin accessibility profiling of mouse and chicken developing face to construct a comparative regulatory map. Although both ectodermal and mesenchymal populations display distinct regulatory features in each species, the mesenchyme exhibits markedly greater divergence, pointing to its central role in shaping facial morphology. We further reveal unexpected molecular complexity in the main face-shaping organizer, including a mouse-specific *Shh/Wnt5a* expression domain. At key morphogen loci (*Bmp4*, *Fgf8*, and *Wnt5a*), conserved and lineage-specific enhancers exhibit spatially restricted activity patterns that mirror divergent signaling domains. These findings demonstrate how cis-regulatory evolution modulates conserved developmental programs to generate morphological novelty, providing a valuable resource for studying vertebrate facial evolution.

## Teaser

Facial shape diversity in birds and mammals arises from conserved developmental programs tuned by divergent regulatory landscapes.

## INTRODUCTION

The vertebrate face displays impressive variation in shape and function, reflecting the adaptation of individual species to the environment. The development and shaping of the face occur during embryogenesis and require tight coordination of actions between the surface ectoderm, the underlying mesenchyme, and the developing nervous system. Central to this complex process are developmental organizers, signaling hubs that regulate cellular behaviors and tissue patterning through the release of conserved morphogens such as Hedgehog (Hh), bone morphogenetic proteins (BMPs), fibroblast growth factors (FGFs), and Wingless-related integration site (WNT) proteins (*1*–*5*). Differences in spatial and temporal expression patterns of morphogens have been observed across species and acknowledged as the source of morphological diversity (*1*, *5*–*7*). The importance of precise regulation of gene expression in time and space is further evidenced in numerous congenital syndromes (table S1) (*8*, *9*).

Among the facial developmental organizers, the frontonasal ectodermal zone (FEZ) stands out as a conserved mediator of upper face patterning, particularly in birds and mammals (*1*, *2*, *10*). The FEZ is a transient structure that emerges during the early stages of facial development, just before the fusion of facial prominences, conventionally defined as the dorsal-ventral boundary of *Fgf8* and *Shh* expression in the oral ectoderm (*1*, *11*). Its presence spans a narrow developmental window, approximately embryonic day 10.5 (E10.5) to E11.5 in mouse and Hamburger-Hamilton (HH) stages 20 to 25 in chicken, with peak signaling activity occurring at around E11.5 and HH22 (*1*, *11*–*13*), respectively. These stages represent key reference points for comparative analysis and offer a focused opportunity to investigate the gene regulatory mechanisms shaping facial morphology in birds and mammals. Experimental modulation of morphogen signaling in the FEZ has been shown to influence the three-dimensional geometry of the face, producing a range of morphological outcomes (*1*, *10*, *14*, *15*). While the developmental significance of the FEZ has been well established through a range of evolutionary developmental studies (*6**,* *12*), how evolutionary processes have calibrated gene expression in this organizer across dimensions (time, space, and concentration) remains poorly understood.

Recent advances in single-cell omics have provided powerful tools for dissecting the complexity of craniofacial development (*16*–*20*). However, these studies have often involved profiling of large anatomical regions, such as the whole head or major facial structures like the maxillary and mandibular prominences, thereby comprising a heterogeneous mix of tissues including tongue, oral epithelium, pharyngeal arches, and the forebrain. While these data are valuable for a general understanding of head development, they intrinsically mask localized signaling events of smaller or transient regions. A few studies have refined this approach by focusing on discrete facial subregions, such as the lambdoidal junction (*21*), palatal shelves (*20*, *22*), and the maxillary prominence (*23*), but these remain limited in scope and are predominantly restricted to mouse models. Consequently, high-resolution, cross-species analyses of critical yet understudied domains, such as those harboring facial organizers, are essential for deeper insight into the mechanisms driving the evolution of facial shape variability.

To understand how these region-specific cellular programs arise ultimately requires integrating cell-level information on gene expression programs with developmental stage-, location-, and cell type–specific cis-regulatory elements (CREs). CREs are predominantly located in noncoding regions of the genome, which evolve more rapidly than coding sequences and therefore represent key substrates for evolutionary change (*24*–*26*). A recent multiomics single-cell study of the mammalian neocortex demonstrated that lineage-specific enhancer activity underlies functional differences in cell type specification in mammals (*27*). The divergent regulatory architectures are also believed to control the development of species-specific facial morphology (*28*). Changes in noncoding genomic regions have also been linked to multiple developmental abnormalities. Mutations in enhancer sequences and the resulting pathologies, collectively termed “enhanceropathies,” illustrate how disruption of regulatory elements can lead to congenital disorders (*29*–*33*). Accordingly, understanding how enhancers contribute to craniofacial development and how the regulatory landscapes differ across species is essential for uncovering the genetic and molecular basis of facial diversity and craniofacial disorders.

In this study, we set out to understand how divergent gene expression patterns in facial developmental organizers of birds and mammals are established. We combine single-cell chromatin accessibility and gene expression profiling in mouse and chicken developing faces to characterize and compare the underlying transcriptomic and regulatory landscapes. We revisit the molecular organization of the facial developmental organizers, infer the differences in cellular communication, and identify cell population–specific regulatory elements that jointly control the formation of the distinctive facial shapes in mouse and chicken. We present a reference for future comparative studies exploring the morphological divergence across avian and mammalian species and identify genomic regions with potential implications for facial abnormalities.

## RESULTS

### Single-cell transcriptional profiling uncovers a distinct molecular organization of the developing face in mouse and chicken

First, we characterize the cellular identity and gene expression profiles of the frontal face in mouse and chicken embryos, with the goal of identifying divergent and conserved molecular features. Mouse E11.5 and chicken HH22 represent critical time points for investigating facial morphogenesis. At these stages, the facial prominences are clearly defined, and the FEZ is fully established, exhibiting a peak of signaling activity (fig. S1 and table S2) (*1*, *34*, *35*). Important signaling pathways such as Hh, FGF, BMP, NOTCH, and WNT orchestrate patterning of the face, rendering these stages optimal for comparative analysis (*2*, *4*, *36*).

**Fig. 1.**
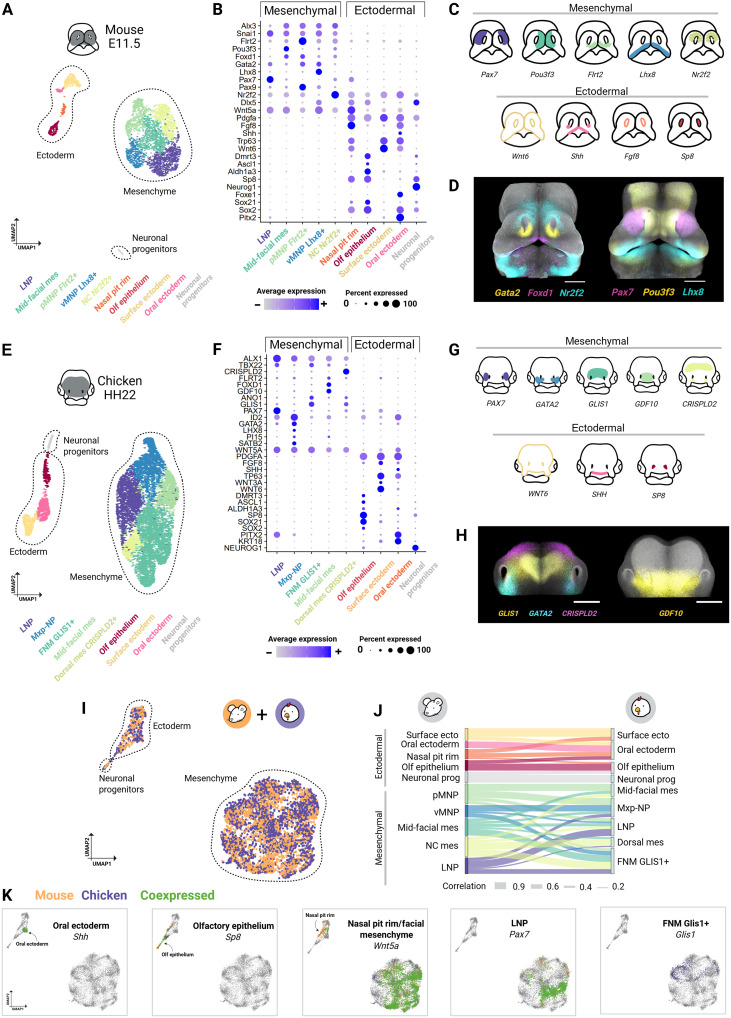
Single-cell transcriptional profiling reveals divergent cell populations in mouse and chicken facial prominences. Facial prominences containing the facial organizer FEZ (gray) were microdissected from mouse (E11.5) and chicken (HH22) embryos. (**A** and **E**) UMAP visualization of transcriptomic data from mouse (A) and chicken (E). Each dot represents a single cell colored by cluster identity. (**B** and **F**) Dot plots of cluster-specific gene expression profiles. Darker colors indicate higher expression, and circle size reflects the percentage of cells expressing the gene. Cluster labels match those in UMAP. (**C** and **G**) Schematic representation of marker gene expression domains confirmed by in situ HCR and image databases. In mice, *Flrt2* is expressed in the pMNP and *Nr2f2* in the NC mesenchyme posterior to the nasal pits (dashed lines). (**D** and **H**) in situ HCR of selected markers. (**I**) Combined UMAP projection of mouse (orange) and chicken (purple) data from SAMap. (**J**) Sankey plot showing homologous clusters between mouse (left) and chicken (right), with line thickness indicating alignment scores. Cluster color-coding matches (A and E). (**K**) UMAP of orthologous gene expression, with connections across species shown in green. Note the unique expression of *GLIS1* in chicken mesenchyme and *Wnt5a* in the mouse nasal pit rim. Scale bars, 500 μm. vMNP, ventral medial nasal prominence; NC, nasal cavity mesenchyme; MXP-NP, mesenchyme spanning maxillary prominence to nasal pits; dorsal mes, dorsal mesenchyme; olf epithelium, olfactory epithelium. Figure created in BioRender. Kyomen, S. (2026) https://biorender.com/g01q360.

We dissected the frontal face, including the FEZ and the underlying mesenchyme, from chicken HH22 and mouse E11.5 embryos. The maxillary and mandibular prominences were removed. Collected tissues were enzymatically dissociated, followed by a single-cell RNA sequencing (scRNA-seq) library preparation pipeline from 10x Genomics. After rigorous quality control, the final scRNA-seq dataset comprised 7475 high-quality cells from chicken and 3342 from mouse, which were subsequently used for analysis (Fig. 1, A and E, and figs. S2 and S3). Unbiased clustering and cluster annotation were performed in both datasets using highly variable genes and classical, well-characterized, cell type–specific markers (Fig. 1, B and F, and table S3). In both species, the cells were primarily categorized into mesenchymal and ectodermal populations, with mesenchymal clusters exhibiting clear positional specificity (Fig. 1, A to H). To validate the identity of each cell cluster, we used in situ hybridization chain reaction (HCR) version 3.1 and cross-referenced findings with publicly available image databases, including EMAGE, GenePaint, and GEISHA (Fig. 1, D and H) (*37*–*39*).

At E11.5 in mice, the frontonasal prominence is divided into medial nasal prominence and lateral nasal prominence (LNP), which flank the nasal pits. Mesenchymal populations recovered from mouse scRNA-seq data comprised LNP, mid-facial mesenchyme (mid-facial mes), posterior medial nasal prominence (pMNP), ventral medial nasal prominence (vMNP), and mesenchyme around the nasal cavity (NC mes). Within the ectoderm, cell clusters were identified as the surface ectoderm, oral ectoderm, nasal pit rim, and olfactory epithelium. A small cluster representing committed neuronal progenitors of the olfactory epithelium was also identified (Fig. 1, A to C, and table S3).

In contrast to the mouse, by stage HH22 in chicken, the frontonasal mass (FNM) consists of a single, flat prominence located at the midline above the oral cavity without a distinct medial-lateral division (fig. S1). These unique anatomical features were well reflected in acquired cell clusters. Five distinct facial mesenchyme populations were identified in the chicken dataset: the FNM, LNP, mid-facial mes, mesenchyme spanning maxillary prominence to nasal pits (MXP-NP), and dorsal frontonasal mesenchyme (dorsal mes) (Fig. 1, E to G, and table S3). Ectodermal populations were categorized as surface ectoderm, oral ectoderm, olfactory epithelium, and committed neuronal progenitors. Contrary to the mouse, a discrete nasal pit rim population in the chicken could not be recovered. However, the chicken nasal pit rim population is represented by the portion of surface ectoderm cells expressing *FGF8* (Fig. 1F). Overall, clustering accurately reflected the known morphological differences between mouse and chicken embryos at these stages.

### Comparative molecular profiling reveals high similarity of facial ectodermal populations

The formation and fusion of facial prominences are a conserved process across amniotes (*40*, *41*). However, observed morphological differences in the prominences across species raise the question of whether the cell populations forming the frontal face express conserved gene expression programs. To evaluate the correspondence of the identified cell populations and their transcriptional programs in mouse and chicken, we used SAMap (self-assembling manifold mapping), an algorithm designed for cross-species alignment and comparison of single-cell transcriptomic data (*42*). This approach enabled us to align homologous gene pairs on the basis of DNA sequence similarity and integrate the single-cell transcriptomes from both species into a unified, lower-dimensional space. Overall, the major cell types within the facial prominences, ectoderm, mesenchyme, and neuronal progenitors, displayed consistent clustering within the joint uniform manifold approximation and projection (UMAP) (Fig. 1I).

Ectodermal populations showed similarity between mouse and chicken, indicating conserved transcriptomic signatures (Fig. 1J and fig. S4). The conservation is particularly evident in the surface ectoderm (marked by *Wnt6* and *Trp63* expression), oral ectoderm (marked by *Shh* and *Pitx2* expression), and olfactory ectoderm [expressing *Sp8*, *Aldh1a3*, *Ascl1*, and *Dmrt3* in both species (Fig. 1, B, F, J, and K)]. The nasal pit rim cluster, marked by *Fgf8* and *Wnt5a* expression in mouse, aligned with both the surface ectoderm and oral ectoderm in chicken. However, *Wnt5a* expression in the nasal pit rim is mouse-specific, as evident in the joint UMAP (Fig. 1K). In contrast, the mesenchymal populations of mouse and chicken displayed greater variation in transcriptomic similarity. Several mesenchymal clusters did not map to a single counterpart in the cross-species alignment but instead showed partial alignment to multiple clusters (Fig. 1J and fig. S4). For example, the mouse MNP showed distributed alignments across several chicken mesenchymal populations, consistent with known differences in midline morphology between species (Fig. 1J and fig. S4). In addition, the chicken FNM, marked by *GLIS1*, *ANO1*, and *TBX22* expression, had no direct counterpart in the mouse dataset, presenting higher similarity with mid-facial mes and nasal cavity mesenchyme (Fig. 1, J and K, and fig. S4). In summary, while ectodermal populations exhibited strong cross-species conservation of their molecular signatures, mesenchymal populations showed substantial variability, highlighting the presence of divergent molecular profiles underlying the known differences in facial shape.

### Inference of cell-cell communication networks reveals signaling interactions of facial organizers

**Fig. 2.**
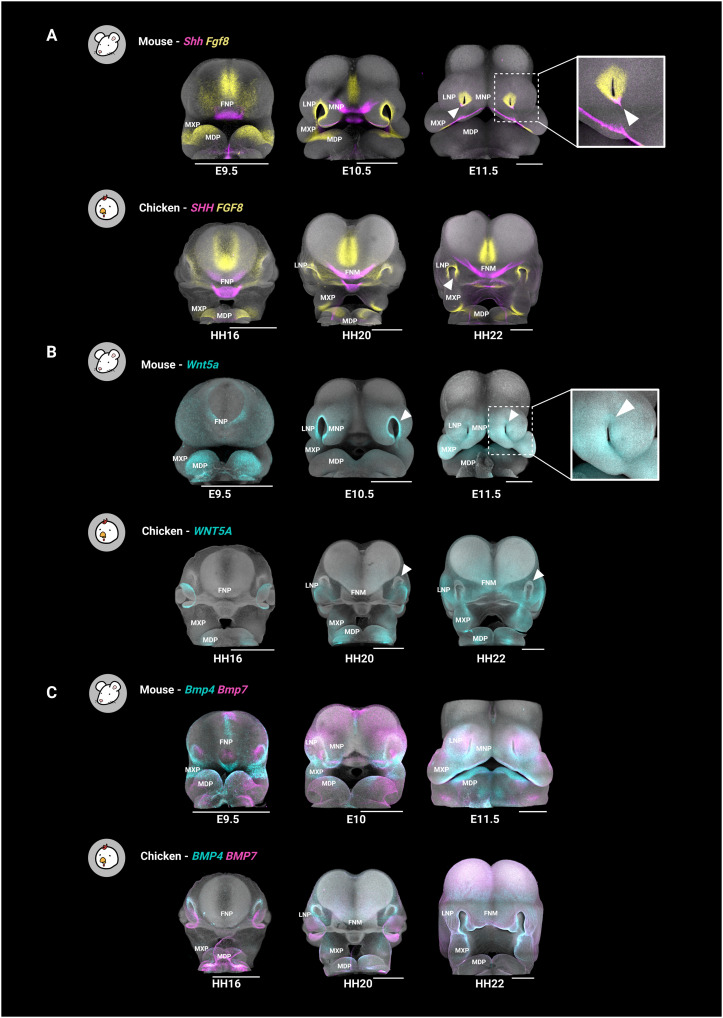
Expression of conserved ligands during early facial development in mouse and chicken. Frontal views of mouse (E9.5 to E11.5) and chicken (HH16 to H22) embryos. Note the unique expression domains of *Shh* in the ventral nasal pit rim [(**A**), white arrowheads] and *Wnt5a* in the nasal pit rim [(**B**), white arrowheads] in mouse. Bmp4/7 expression is observed in both the mesenchyme and ectoderm in both species (**C**). Scale bars, 500 μm. FNP, frontonasal process; MXP, maxillary prominence; MDP, mandibular prominence; MNP, medial nasal prominence. Figure created in BioRender. Kyomen, S. (2026) https://biorender.com/g01q360.

Facial development is orchestrated by intricate signaling interactions between ectodermal and mesenchymal tissues, yet the evolutionary conservation of these interactions remains largely unexplored. To investigate conserved and divergent epithelial-mesenchymal cross-talk in mouse and chicken, we applied CellChat, a tool for inferring and visualizing cell-cell communication networks from single-cell transcriptomic data (*43*). To assure a robust comparison of mouse and chicken datasets, we have restricted our analysis to one-to-one orthologs obtained from the SAMap homology table (see Materials and Methods). Our analysis specifically focused on secreted signaling pathways, which involve ligands diffusing through the extracellular space to activate cell surface receptors and influence tissue behavior. Populations with a higher expression of ligands are designated as “senders,” while those expressing the cognate receptors are identified as “receivers.” Our interpretations further center on WNT, BMP, Hh, NOTCH, and FGF signaling pathways because of their critical roles in face development (table S1) (*5*, *44*). To complement the computational predictions and provide spatial and temporal context, we performed in situ HCR to map ligand expression patterns across early facial development, from E9.5 to E11.5 in mouse and HH16 to HH22 in chicken (Fig. 2).

**Fig. 3.**
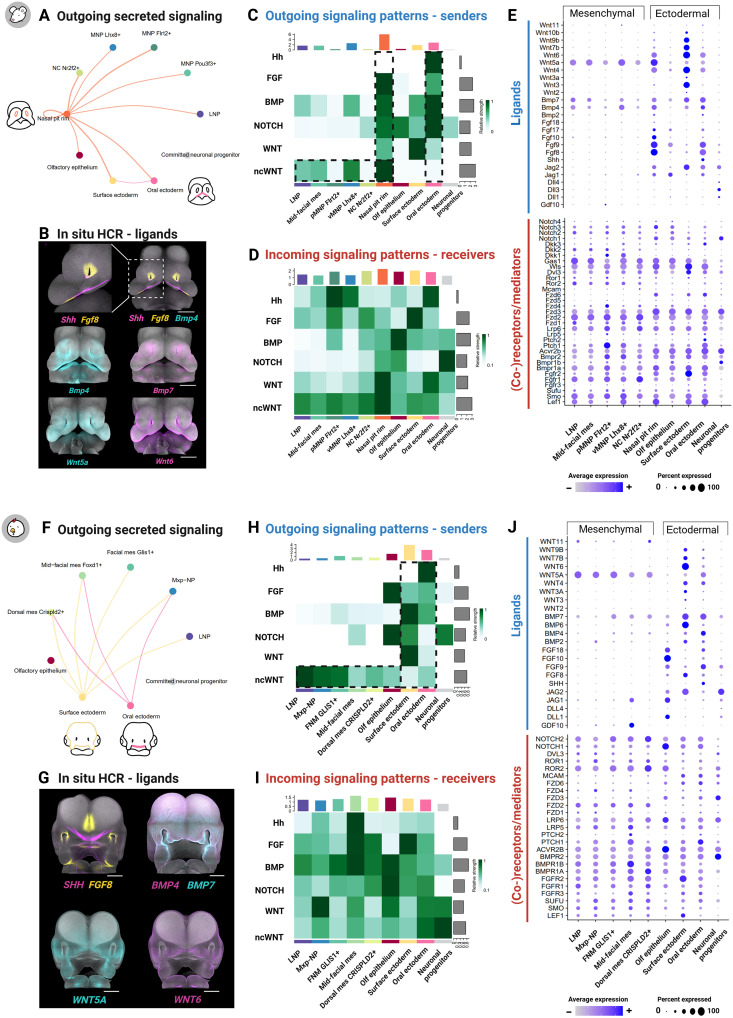
Inference of cell-cell communication networks of facial organizers. (**A** and **F**) Circle plots showing inferred communication between cell populations using secreted signaling pathways. The linewidth indicates communication probability, and color represents secreted signaling sender. (**B** and **G**) In situ HCR of selected ligands. (**C** and **H**) Heatmap showing the contribution of cell clusters to the outgoing secreted signaling activity of major morphogen families. Color bars indicate the contribution of each cluster to secreted signaling, while gray bars denote the relative importance of each pathway to secreted signaling. (**D** and **I**) Heatmap illustrating the contribution of cell clusters to incoming secreted signaling activity with broad distribution of signal-receiving populations. (**E** and **J**) Dot plot of gene expression for selected ligands, (co-)receptors, mediators, and regulators associated with major morphogen families in mouse (E) and chicken (J). Scale bars, 500 μm. Panels (B) and (G) are reproduced from Fig. 1 (A to C) to facilitate a direct comparison with outgoing signaling patterns inferred by CellChat. Figure created in BioRender. Kyomen, S. (2026) https://biorender.com/g01q360.

Ectodermal populations in both mouse and chicken emerged as the primary sources of secreted signaling, positioning them as central hubs in coordinating facial morphogenesis (Fig. 3, A and F). Among these signaling sources, the oral ectoderm secretes ligands of the Hh, FGF, and BMP pathways in both species, reinforcing its role in orchestrating facial morphogenesis (Fig. 3, B, C, G, and H). The oral ectoderm harbors the FEZ, a key developmental organizer conventionally defined by the expression boundary of *Shh* and *Fgf8*. While previous studies described these expression domains as mutually exclusive (*1**,* *11*), in situ HCR revealed a more complex molecular organization, with *Shh*, *Fgf8*, and *Bmp4* colocalizing in specific regions of the oral ectoderm in both species (figs. S5 and S6). This suggests an underappreciated level of molecular complexity within the FEZ, challenging the classical model of strict morphogen boundaries (*1**,* *11*). Moreover, communication predictions pointed at Hh signaling as a highly conserved epithelial-to-mesenchymal communication axis in both species. In mouse, mesenchymal cells adjacent to the *Shh*-expressing ectoderm, specifically the pMNP, were the primary recipients of Hh signals (Fig. 3D). A similar pattern was observed in chicken, where mid-facial mes, positioned near the *Shh*-positive oral ectoderm, was the strongest receiver of Hh signaling (Fig. 3I).

In mouse, the nasal pit rim emerged as a prominent signaling center, secreting ligands from noncanonical WNT, FGF, and BMP pathways (Fig. 3, A to C). These findings align with previous studies suggesting the role of the nasal pit rim in mid-face and nasal cavity development (*45*, *46*). Notably, in situ HCR revealed a unique Hh signaling domain in the ventral nasal pit rim of mouse embryos at E11.5, a previously overlooked domain in mouse that is absent in chicken (Fig. 3, B and G, and figs. S5 to S7). In addition, while *Bmp4* expression was detected in the nasal pit ectoderm of both species, *Wnt5a* and *Wnt6* expression domains were exclusively present in mouse (Fig. 3, B and G, and figs. S5 to S7). This suggests that the nasal pit rim serves as a specific source of Hh and canonical and noncanonical WNT signaling in mouse and underscores notable divergence in the molecular organization of facial organizers in the two analyzed species. In contrast, in chicken, the surface ectoderm was pointed out as a major contributor to outgoing signaling, particularly through the secretion of canonical WNT, BMP, and FGF ligands (Fig. 3, F and H).

Additional differences between mouse and chicken were observed in the expression of NOTCH pathway ligands. Canonical ligands such as *Jag1*, *Jag2*, and *Dll1* were predominantly expressed in the olfactory epithelium of both species, especially within neuron-rich regions, as confirmed by in situ HCR (fig. S7). Notably, in mouse embryos, *Jag1-2* also showed detectable expression in the surface, oral ectoderm, and the nasal pit rim, a pattern not detected in chicken (fig. S7). These observations indicate that while NOTCH signaling is conserved in neuron-rich regions at the assessed stages, the oral ectoderm and nasal pit rim act as additional sources of ligand secretion in mouse, highlighting differential spatial deployment of NOTCH signaling across species. The spatial pattern of NOTCH ligand expression observed at the analyzed stage precedes the well-characterized roles of NOTCH signaling in later craniofacial morphogenesis, such as secondary palate formation (*47*).

The mesenchymal populations also contribute to cell-cell communication, particularly through noncanonical WNT and BMP pathways. In both species, noncanonical WNT ligand secretion displayed a wider distribution, originating from several mesenchyme clusters (Fig. 3, C, E, H, and J). While BMP ligands are predominantly secreted by the surface ectoderm in chicken and the oral and nasal pit rim ectoderm in mouse, BMP signaling activity is also detected within the mesenchyme in both species (Fig. 3, C, E, H, and J). In situ HCR confirmed that *Wnt5a* and *Bmp4/7* exhibit distinct expression domains within the underlying mesenchyme (Figs. 2, B and C, and 3, B and G). These results suggest that the facial mesenchyme is not merely a passive recipient of epithelial signals but actively shapes the morphogenetic signaling landscape by locally producing extracellular cues for specific pathways.

In general, while ligand secretion was found to be rather specific to certain cell populations, the corresponding receptors, co-receptors, and signaling mediators were found to be broadly expressed across both ectodermal and mesenchymal populations in both species (Fig. 3, E and J). This widespread expression of receptors and mediators enables both ectodermal and mesenchymal cells to respond to diverse signals from distinct sources, promoting diverse cellular responses along the facial morphogenesis. Overall, the cell-cell communication inference supported the utilization of conserved molecular tools mediating epithelial-mesenchymal interactions but highlighted the evolutionary divergence of signaling spatial domains.

To place our findings in a broader evolutionary context, we reexamined recent work in lizards (*Anolis sagrei*), in which deviations from the canonical FEZ organization have recently been proposed (*48*). Previous analyses reported that at early stages [stage 3 (*49*)], *SHH* expression is restricted to the ectoderm lining the posterior oral cavity and is absent from the oral ectoderm adjacent to the forebrain in later stages. In addition, previous observations describe *FGF8* being expressed in the surface ectoderm at early stages but absent from the midline oral ectoderm later in development, with expression in the nasal pit detected only at later stages (*48*).

Using in situ HCR across stages 3 to 6 (figs. S8 and S9), we confirm several of these observations, such as the *SHH* expression restricted to the posterior oral ectoderm at stage 3. However, our data further reveal features of FEZ organization that were not previously described. Beginning at stage 3, *SHH* and *FGF8* form a sharp interface within the ectoderm of the maxillary prominence, comparable to that observed in mouse and chicken embryos (fig. S9). At stages 5 and 6, *SHH* expression expands anteriorly to encompass the oral ectoderm adjacent to the forebrain, covering the roof of the mouth and overlapping with *FGF8* in discrete regions of the anterior oral ectoderm, a configuration resembling the one we observed in mouse and chicken (figs. S5, S8, and S9). Moreover, *FGF8* expression in the nasal pit is already present from stage 3 onward (figs. S8 and S9). Consistent with the expression patterns observed in chicken, *SHH* expression is absent from the ventral rim of the nasal pit at all stages examined in *Anolis*.

Together, these results demonstrate that *Anolis* exhibits a conserved FEZ signaling architecture characterized by sustained *SHH-FGF8* interfaces within the oral ectoderm and maxillary prominence. Notably, certain features, such as *SHH* expression at the nasal pit rim (observed in mouse but not in chicken or *Anolis*), reflect modest lineage-specific variation within an otherwise conserved FEZ framework. However, we acknowledge that more definite conclusions regarding whether FEZ and its deployment can be extrapolated to all amniotes will depend on broader taxonomic sampling.

### Chromatin accessibility profiling highlights evolutionary divergence in facial development regulation

**Fig. 4.**
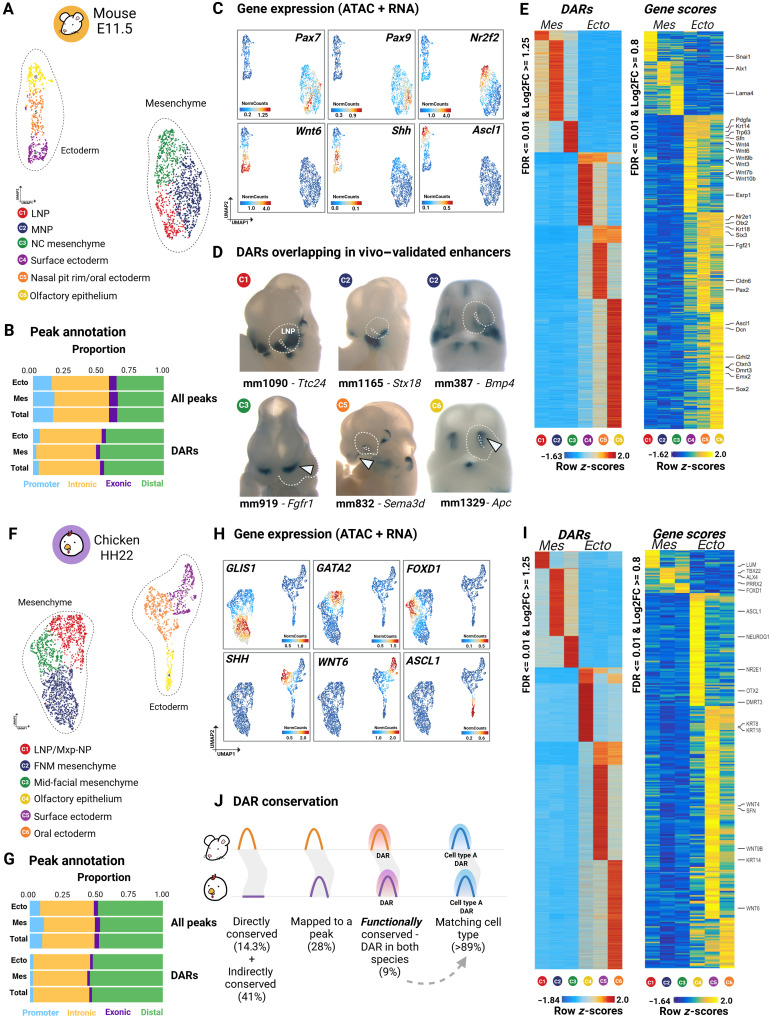
Chromatin accessibility profiling reveals evolutionary divergence in facial development regulation. (**A** and **F**) UMAP visualization of mouse and chicken scATAC-seq data. Each dot represents a single cell colored by its cluster. (**B** and **G**) Bar plots showing peak and DAR annotations by their genomic position across ectodermal and mesenchymal populations. The “total” bar represents the final peak set. Note the larger proportion of distal and intronic regions in DARs compared to exonic and promoter elements. (**C** and **H**) UMAPs showing inferred expression of selected marker genes based on integrated scATAC-seq and scRNA-seq data. (**D**) Transgenic E11.5 mouse embryos show the tissue-specific activity of cCREs identified in mouse DARs. White arrowheads point at enhancer activity in the NC mesenchyme (mm919), oral ectoderm (mm832), and olfactory epithelium (mm1329). (**E** and **I**) (Left) Heatmaps illustrating DARs in each cell population, with distinct profiles for mesenchymal and ectodermal clusters. (Right) Heatmaps of gene scores for cell-specific marker genes. (**J**) Schematic representation of conservation levels assessed through IPP. Peaks were classified as directly conserved (projection score >0.98; 14.3% for mouse-chicken), indirectly conserved (score >0.84; 41.1%), or nonconserved (score <0.84; 44.4%). Functionally conserved DARs were defined as DARs in one species that aligned to a DAR in the other species, regardless of cell type correspondence. Only a small fraction of DARs were functionally conserved (9% of mouse and 2% of chicken), yet the majority retained the same cell type identity across species. Figure created in BioRender. Kyomen, S. (2026) https://biorender.com/g01q360.

Our analyses demonstrated that the developing face is organized into multiple cell populations with distinct transcriptional identities, reflecting the complex cellular architecture underlying facial morphogenesis and the differences between species. To explore the cis-regulatory landscapes that establish cell- and population-specific gene expression programs in the embryonic face, we performed single-cell analysis of transposase-accessible chromatin using sequencing (scATAC-seq) on microdissected frontal face of mouse (E11.5) and chicken (HH22) embryos. After stringent quality control, we gathered information on the chromatin accessibility of 1879 cells in mouse and 2836 cells in chicken (Fig. 4, A and F; fig. S10; and table S4). The annotation of accessible chromatin regions revealed the enrichment of distal and intronic peaks, with depletion of promoter regions, a trend commonly observed in scATAC-seq data (Fig. 4, B and G) (*50*). Peak calling annotations were comparable across both datasets, confirming the robustness and reproducibility of the assays (Fig. 4, B and G, and table S4).

Unbiased iterative clustering identified six clusters in both species, initially annotated using gene activity scores that reflect the predicted expression levels on the basis of the accessibility of nearby regulatory elements (Fig. 4, A and F) (*51*). Well-characterized, cell type–specific genes were used to support cluster identity (Fig. 4, C, E, H, and I). To further refine these annotations, we integrated the scRNA-seq and scATAC-seq datasets (figs. S11 and S12). Enhancer-promoter coaccessibility of ATAC peaks, known as peak-to-gene links, was then inferred to provide a more in-depth characterization of CREs, linking transcriptional activity to the underlying regulatory landscape (figs. S11 and S12).

In both species, scATAC-seq data revealed distinct chromatin accessibility profiles between ectodermal and mesenchymal populations (Fig. 4, E and I). In the mouse scATAC-seq dataset, mesenchymal clusters were annotated as LNP, MNP, and nasal cavity mesenchyme, while ectodermal clusters were identified as the surface ectoderm, oral ectoderm/nasal pit rim, and olfactory epithelium. After integration, some mouse scATAC-seq clusters aligned with multiple RNA-seq clusters (fig. S11). Specifically, the oral ectoderm and nasal pit rim clusters in mouse could not be separated upon integration. Mouse peaks overlapped with in vivo–validated enhancers with reported activity in forming facial prominences, providing additional support for cluster annotation (Fig. 4D). In the chicken, scRNA-ATAC integration revealed a clear distinction of main cell clusters (fig. S12). Chicken mesenchymal clusters from scATAC-seq were classified as LNP/MXP-NP, FNM, and mid-facial mes, while ectodermal clusters included the olfactory epithelium, surface ectoderm, and oral ectoderm.

To uncover regulatory programs driving cellular identity in the facial populations, differentially accessible regions (DARs) were identified for each scATAC cluster. This analysis revealed 12,096 DARs in the mouse dataset and 45,030 in the chicken dataset (Fig. 4, E and I, and table S4). The larger number of DARs in the chicken dataset is likely due to the higher cell count and the higher number of unique fragments in chicken (fig. S10). Cell type–specific peaks were largely enriched in distal intergenic and intronic regions, showing lower overlap with promoters and exonic regions compared to the full set of accessible chromatin peaks (Fig. 4, B and G). These findings align with previous studies reporting that distal regulatory elements are more frequently associated with cell- or tissue-specific chromatin accessibility than promoters (*27*, *52*–*54*).

Ectodermal populations represent more specialized cell groups (e.g., surface versus olfactory ectoderm), exhibiting more prominent regulatory differences that yield abundant cluster-specific DARs (Fig. 4, E and I, and table S4). In contrast, the facial mesenchyme at the analyzed stage is known to exhibit heterogeneity related to positional programs rather than fate commitment or functional specialization (*55*). Consistent with these transcriptomic differences, mesenchymal heterogeneity is less frequently resolved as cluster-specific DARs (Fig. 4, E and I, and tables S3 and S4). This contrast between the more prominent ectodermal regulatory complexity and the subtler mesenchymal regulatory complexity was consistent across species and robust to alternative clustering resolutions and DAR detection thresholds, indicating that it does not arise from differences in cluster size, sequencing depth, or quality control metrics. Thus, while chromatin accessibility reflects the specialization among ectodermal populations, transcriptional profiling better captures the more gradual positional heterogeneity within the mesenchyme, illustrating how the two modalities report distinct but complementary dimensions of cellular heterogeneity.

To assess the evolutionary conservation of the identified CREs, we compared chromatin accessibility profiles from mouse and chicken embryos using interspecies point projection (IPP), a synteny-based approach that maps orthologous genomic positions without relying on direct sequence similarity (*56*). In this approach, genomic coordinates are projected between species through intermediate genomes using conserved synteny, enabling the identification of corresponding loci even when local sequence similarity is low or absent. To place these comparisons within a broader evolutionary context, we included six additional vertebrate species as bridging outgroups (table S4). Mouse and chicken peaks were reciprocally projected between species and through each bridging genome to quantify positional conservation across evolutionary timescales. Accessible regions were then classified as directly conserved (projection score >0.98), indirectly conserved (score >0.84), or nonconserved (score <0.84). Higher projection scores indicate shorter distances to syntenic anchor points and, therefore, greater confidence in positional conservation.

Approximately 14% of mouse peaks could be directly mapped to the chicken genome, while 41% were indirectly conserved (Fig. 4J, fig. S13, and table S4), demonstrating that most regulatory regions are not easily traceable using sequence conservation across this evolutionary distance. Human and opossum were the most frequently used bridging species for cross-species projections (table S4). Only 28% of these conserved peaks (direct + indirect) overlapped with an open chromatin region in chicken, suggesting limited preservation of chromatin accessibility across species. To assess evolutionary conservation at the level of regulatory function, we defined cell type–specific DARs as functionally conserved if they aligned to a DAR in the other species, irrespective of cell type correspondence. Under this definition, only 9% of cluster-specific DARs in mouse and 2% of DARs in chicken were functionally conserved, indicating extensive divergence in the regulatory grammar between the two species (Fig. 4J, fig. S13, and table S4). Notably, most functionally conserved DARs retained the same cell type identity across species. For example, functionally conserved ectodermal DARs in chicken predominantly corresponded to ectodermal DARs in mouse (Fig. 4J, fig. S13, and table S4). Functionally conserved DARs were markedly more frequent in the ectoderm than in the mesenchyme. Across both species, mesenchymal DARs showed significantly lower conservation compared to ectodermal DARs (*P* < 0.001), corresponding to an ~4.4-fold higher conservation rate in ectoderm cells, independent of the species (fig. S13). These results agree with SAMap analysis, which independently indicated higher cross-species transcriptional similarity among ectodermal populations than among mesenchymal populations. Regulatory landscapes in the developing face have diverged extensively between mouse and chicken, with limited functional conservation of cell type–specific accessible regions and a pronounced bias toward conservation in ectodermal cell types.

To assess the potential biological significance of functionally conserved DARs, a gene ontology enrichment analysis was conducted using Genomic Regions Enrichment of Annotations Tool (GREAT), which assigns noncoding genomic regions to nearby genes on the basis of defined regulatory domains and tests whether gene ontology terms are statistically overrepresented among the associated genes compared to a genomic background (fig. S14 and table S4) (*57*). Consistent with their noncoding nature, genomic regions associated with functionally conserved DARs were enriched for terms related to the regulation of transcription, transcription factor (TF) complexes, and DNA binding (fig. S14). The analysis also revealed notable associations between functionally conserved DARs and genes linked to craniofacial phenotypes in both mouse knockout studies and human disorders, including the abnormality of the face, mouth, and skeleton (fig. S14). These findings are consistent with the distinct cellular fates of mesenchymal and ectodermal derivatives in later stages of craniofacial development.

### In vivo mapping of craniofacial enhancer activity identifies variation in the regulation of morphogen signaling

To better understand the regulatory mechanisms driving facial morphogenesis, candidate CREs (cCREs) involved in this process were identified and characterized. scATAC-seq peaks were annotated on the basis of genomic location (e.g., intronic, distal, and intergenic), overlap with publicly available enhancer databases [e.g., VISTA Enhancer Browser (*58*)], and chromatin immunoprecipitation sequencing (ChIP-seq) data targeting enhancer-associated histone modifications (e.g., H3K27ac). Evolutionary conservation, indicated by UCSC PhyloP (*59*), was also considered. The accuracy of candidate cis-regulatory sequence (cCRE) discovery was further improved by inferring enhancer-promoter coaccessibility and inspecting coverage plots.

In addition, ChIP-seq profiling for the active enhancer mark H3K27ac was performed on chicken embryos at HH22. Following stringent quality control and integration of two biological replicates, 15,972 high-confidence peaks were identified, with 1346 regions overlapping the chicken scATAC-seq dataset (table S5). Consistent with prior studies of noncoding regulatory landscapes, 97% of these peaks localized to intronic or intergenic regions (table S5). This ChIP-seq H3K27ac dataset complements the chicken scATAC-seq data by providing a broader map of active regulatory regions, jointly forming a valuable resource to prioritize candidate enhancers for future functional studies in avian species.

By cross-referencing mouse scATAC-seq peaks with the VISTA Enhancer Browser, we identified 1082 in vivo–validated enhancers with demonstrated activity at E11.5. Of these, 246 enhancers intersected cell type–specific DARs from our scATAC-seq dataset from mouse. These enhancers exhibit activity across a range of embryonic tissues, including frontonasal mesenchyme, nose, and branchial arches (fig. S15). Moreover, several of these regulatory regions demonstrated activity in specific subregions in the face (Fig. 4D and figs. S16 and S17). For instance, mm1416 showed strong activity in the ectoderm surrounding the nasal pit rim, aligning with chromatin accessibility patterns in our dataset (fig. S16). A DAR identified in the olfactory epithelium, corresponding to enhancer mm1329, drove reporter activity in this specific facial region in LacZ transgenic embryos (fig. S16). In mesenchymal populations, a DAR in the NC mesenchyme cluster overlapped with mm919, an enhancer with demonstrated activity in the posterior nasal cavity (fig. S17). These findings reinforce the predictive power of this dataset for identifying active regulatory elements and provide functional insights into their roles in both facial development and sensory organ development. The comprehensive list of 1082 VISTA enhancers that intersect peaks in our scATAC-seq dataset, along with their validated tissue activity and genomic coordinates, is provided in table S6. Images of transgenic embryos identified in this study are provided in fig. S18.

**Fig. 5.**
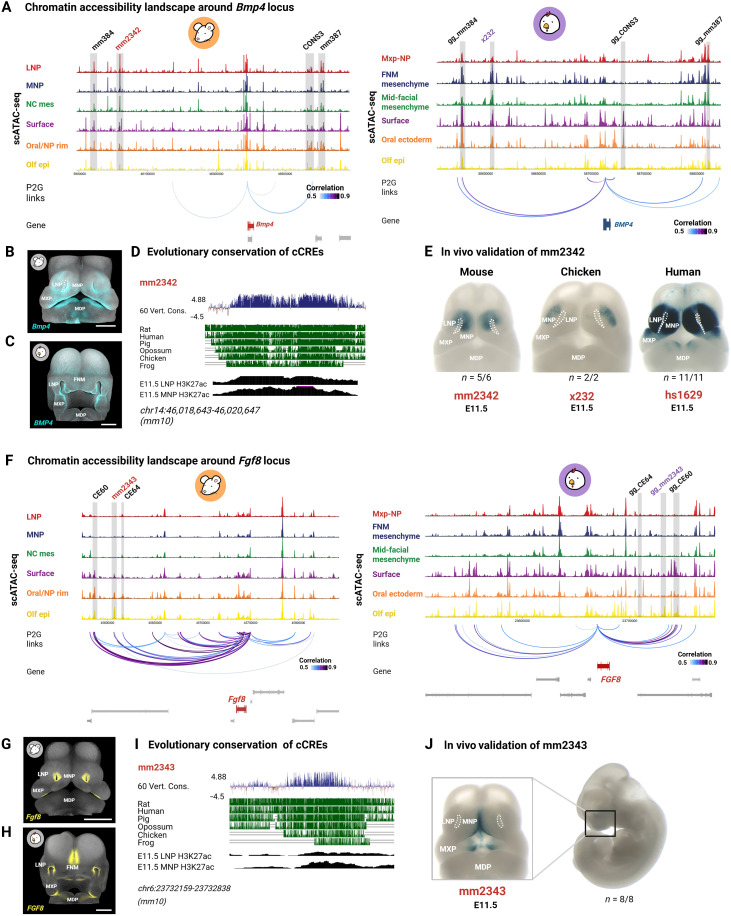
Enhancer activity and chromatin accessibility near *Bmp4* and *Fgf8* loci. (**A**) Genome track showing the *Bmp4* locus, with scATAC-seq signals from mouse (left) and chicken (right) colored by cell clusters. Gray boxes highlight cCREs in mouse, with corresponding regions in chicken. Red/purple labels indicate cCREs identified in this study. Inferred peak-to-gene links between cCREs and the *Bmp4* and *Fgf8* promoters are shown below, with darker colors indicating stronger correlations. (**B** and **C**) In situ showing *Bmp4* expression in mouse E11.5 (B) and chicken HH22 (C) embryos. (**D**) Evolutionary conservation of mm2342, with PhyloP scores (60-way vertebrate alignment) and conservation tracks. Coverage plots of ChIP-seq (H3K27ac) from mouse E11.5 LNP and MNP are displayed below. (**E**) Transgenic E11.5 mouse embryos showing the enhancer activity of mm2342 in the LNP and MNP mesenchyme, alongside its chicken (x232) and human (hs1629) orthologs. Note the variation in LacZ staining patterns in the facial region depending on the orthologous version of the enhancer. (**F**) Genome track of the *Fgf8* locus. (**G** and **H**) *Fgf8* expression in mouse E11.5 (G) and chicken HH22 (H) embryos. (**I**) Evolutionary conservation of mm2343. (**J**) Transgenic mouse E11.5 embryos showing the enhancer activity of mm2343 in the surface and oral ectoderm. Scale bars, 500 μm. Panels (B), (C), (G), and (H) are reproduced from Fig. 1, A and C to facilitate a direct comparison with mm2342 and mm2343 enhancer activities in transgenic mouse embryos. Figure created in BioRender. Kyomen, S. (2026), https://biorender.com/g01q360.

Next, we explored the regulatory landscape of selected morphogens in more detail. We focused on *Bmp4*, *Fgf8*, and *Wnt5a*, which play well-established roles in facial development at the analyzed stage but whose cis-regulatory architectures remain comparatively underexplored, particularly across species, in contrast to *Shh*, whose regulatory landscape has been extensively characterized (*60*). The scATAC-seq data revealed that several known enhancers, e.g., mm384, mm387, hs1629, and CONS3, were accessible in *Bmp4*+ facial populations, including mouse LNP, MNP, nasal pit rim, and oral ectoderm (Fig. 5, A and B) (*61*, *62*). Orthologs of these cCREs were also accessible in *BMP4*+ populations in the chicken scATAC-seq dataset, suggesting a conserved regulatory role in face formation across birds and mammals (Fig. 5, A to C). A peak of particular interest, mm2342, was identified as the murine ortholog of the human hs1629 cCRE (Fig. 5, A and E, and fig. S19). Located ~360 kb upstream of *Bmp4*, mm2342 was primarily accessible in mesenchymal clusters, particularly in the LNP and MNP in mouse (Fig. 4A). In chicken, the orthologous genomic region exhibited accessibility across multiple clusters, including the FNM mesenchyme, mid-facial mes, and ectodermal populations, such as the surface ectoderm and oral ectoderm, all of which express *BMP4* (Fig. 5, A and C). Comparative analysis across species revealed that mm2342 is highly conserved in vertebrates (Fig. 5D and fig. S19). In vivo activity mapping confirmed its activity in the LNP and MNP mesenchyme, aligning with the endogenous *Bmp4* expression domain in mouse (Fig. 5E). mm2342 appears to have more spatially restricted activity compared to hs1629 (Fig. 5E). Its chicken ortholog, x232, shows enhancer activity specifically confined to the LNPs, suggesting a specialized and species-dependent regulatory function (Fig. 5E). Notably, mm2342 is located within a topologically associating domain (TAD) that includes its predicted target gene, *Bmp4*, as revealed by Hi-C data from E11.5 mouse facial prominences (fig. S20) (*63*).

The *Lbx1-Fgf8* region contains known *Fgf8* CREs in mice, particularly active in the limb and midbrain-hindbrain boundary (*64*, *65*). Within this genomic region, a peak, mm2343, was identified ~1700 base pairs (bp) within the intronic region of *Fbxw4* and downstream of *Fgf8* in mouse (Fig. 5F). In mouse, mm2343 showed high chromatin accessibility specifically in ectodermal populations expressing *Fgf8*, with strong coaccessibility to the *Fgf8* promoter (Fig. 5, F and G). In chicken, the corresponding region of mm2343 was accessible in the olfactory epithelium cluster, despite *FGF8* expression being more restricted to the nasal pit rim (Fig. 5, F and H). Evolutionary analysis revealed that mm2343 is highly conserved among mammals, with partial conservation extending to other tetrapod lineages (Fig. 5I and fig. S19). In vivo validation confirmed that mm2343 functions as an enhancer in the developing face, with activity specific to the oral and surface ectoderm (Fig. 5J). Consistent with its regulatory role, mm2343 resides within a TAD that includes the *Fgf8* gene, as shown by Hi-C data (fig. S20).

**Fig. 6.**
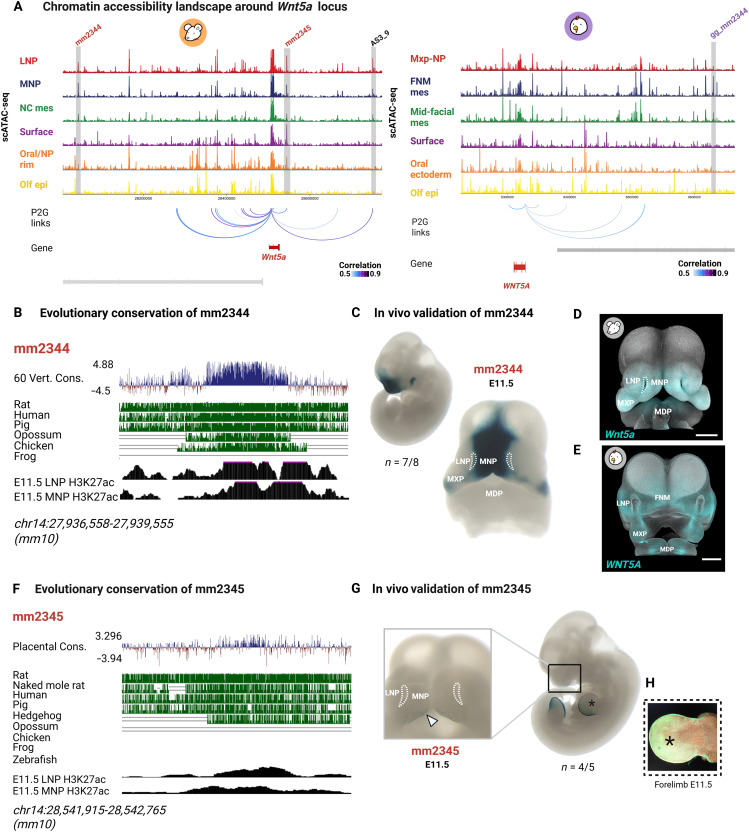
Enhancer activity and chromatin accessibility near the *Wnt5a* locus. (**A**) Genome track showing the *Wnt5a* locus in mouse (left) and chicken (right), with aggregated scATAC-seq signals for cell clusters colored by cluster assignment. Gray boxes highlight cCREs in mouse with corresponding regions in chicken. Red/purple labels indicate cCREs identified in this study. Inferred peak-to-gene links to the *Wnt5a* promoter are shown below, with darker colors indicating stronger correlations. (**B**) Evolutionary conservation of mm2344, with PhyloP conservation scores and tracks. Coverage plots of ChIP-seq (H3K27ac) from mouse E11.5 LNP and MNP are displayed below. (**C**) Transgenic mouse E11.5 embryos showing the strong enhancer activity of mm2344 in the MNP mesenchyme. (**D** and **E**) Wnt5a expression in mouse E11.5 (D) and chicken HH22 (E) embryos. (**F**) Evolutionary conservation of mm2345, with PhyloP conservation scores and tracks. (**G**) Transgenic mouse E11.5 embryos showing the enhancer activity of mm2345 in the oral ectoderm. (**H**) *Wnt5a* expression in the developing mouse limb resembles mm2345 enhancer activity in the limbs. Scale bars, 500 μm. Panels (D) and (E) are reproduced from Fig. 1B to facilitate a direct comparison with mm2344 and mm2345 enhancer activities in transgenic mouse embryos. Figure created in BioRender. Kyomen, S. (2026), https://biorender.com/g01q360.

The regulatory landscape of *Wnt5a*, a morphogen essential for oriented snout outgrowth and guiding cell migration during palate development (*66*, *67*), was further explored. A peak was identified within the genomic region surrounding the *Wnt5a* locus, overlapping the previously characterized AS3_9 enhancer, known for its strong activity in the face and palate of mice at E14.5 (Fig. 6A) (*68*). In addition, two putative CREs, mm2344 and mm2345, were found around 600 kb upstream and 400 kb downstream of the *Wnt5a* promoter, respectively (Fig. 6A). mm2344 exhibited distinct chromatin accessibility within cell clusters, primarily confined to the LNP and MNP. An orthologous genomic region in chicken displayed accessibility restricted to the FNM mesenchyme (Fig. 6A). Evolutionary conservation analysis demonstrated that mm2344 is conserved in vertebrate lineages (Fig. 6B and fig. S19). In vivo activity mapping revealed the strong activity of mm2344 in the mid-facial mes and posterior MNP and MXP, aligning with *Wnt5a* expression in both mouse and chicken (Fig. 6, C to E). In contrast, mm2345 was accessible in the LNP, MNP, and oral ectoderm/nasal pit rim clusters, showing coaccessibility with the *Wnt5a* promoter (Fig. 6A). Conservation analysis revealed that mm2345 is conserved across mammals but not detected in chicken. This conclusion is further supported by the IPP analysis, in which mm2345 was the only novel CRE described in this study that failed to project to the chicken genome (table S4). Together, these results point to divergence in the regulatory landscape controlling *Wnt5a* expression (Fig. 6E and fig. S19). Functional validation of mm2345 confirmed its enhancer activity in the MNP and oral ectoderm (Fig. 6G). Notably, mm2345 also exhibited enhancer activity in the ectoderm and mesenchyme of emerging limb buds at E11.5, mirroring *Wnt5a* expression in these regions (Fig. 6H). Hi-C data from E11.5 mouse facial prominences show that mm2344 lies within a TAD containing *Wnt5a*, supporting its potential to interact with the gene promoter (fig. S20). In contrast, mm2345 is located near the *Wnt5a* promoter, harboring a TAD boundary (fig. S20).

Overall, these findings reveal cell population–specific CREs operating during facial development, including both conserved and evolutionary divergent enhancers within the regulatory landscapes of conserved morphogens *Bmp4*, *Fgf8*, and *Wnt5a*. The results highlight the interplay between conserved and divergent regulatory mechanisms in building and shaping vertebrate craniofacial structures.

### Analysis of TF binding motifs identifies lineage-specific TF recruitment in facial prominences

Regulatory elements harbor clusters of TF binding sites (TFBSs) that facilitate the simultaneous binding of multiple TFs and cofactors. This arrangement enables enhancers to integrate diverse regulatory signals in a context-dependent manner, reflecting developmental stage, cell type, or tissue specificity. Variation in both the expression of TFs and sequence composition of TFBSs contributes to the evolution of specific gene regulatory landscapes. To reveal TFBSs within cell population–specific DARs, these regions were screened using motif enrichment analysis in mouse and chicken.

A subset of TF families showed enrichment in corresponding clusters in both species (figs. S21A and S22A). For example, motifs for the p53 family of TFs, particularly TRP63, were enriched in the regulatory elements active in surface ectoderm, reflecting their known role in epidermal specification in vertebrates (figs. S21B and S22B) (*69*, *70*). SOX gene family motifs were similarly prevalent in the olfactory epithelium in both species, consistent with their involvement in neurogenic differentiation (figs. S21B and S22B) (*71*).

The motif enrichment analysis additionally pinpointed differences across species. In the mesenchyme, FOX family TF motifs displayed distinct distribution patterns between mouse and chicken. These TFs are important regulators of chromatin accessibility in neural crest–derived cells during facial cartilage formation (*72*). In mouse, FOX motifs were predominantly enriched within the regulatory elements operating in the mesenchyme surrounding the nasal cavity, while in chicken, they appeared more prominently in the mid-facial mes (figs. S21B and S22B). SMAD2 motifs also exhibited variation, with enrichment in the oral ectoderm/nasal pit rim cluster in mouse, while displaying a broader distribution across mesenchymal populations in chicken (figs. S21C and S22C). SMAD2 has been implicated in beak morphogenesis and is known to have a prominent role as a mediator in the BMP signaling pathway (*73*, *74*). PBX3 motifs, involved in regulating *Wnt9b* expression in the surface ectoderm, were highly accessible in ectodermal clusters in chicken, supporting prior findings (fig. S22C) (*75*). In addition, LEF1 (lymphoid enhancer binding factor 1) motifs were enriched in the oral/nasal pit rim cluster of mouse, where it is expected to interact with WNT signaling components to promote tooth development (fig. S21C) (*76*).

To further determine whether enriched TFBSs correspond to active TF recruitment in specific cell populations, TF footprinting analysis was performed. This approach, leveraging DNA accessibility profiles, identifies binding events at enriched motifs (*51*). Positive footprint signals, indicating the physical binding of a factor to a regulatory element, emerged for known lineage-specific regulators in both species. GRHL1 (grainyhead-like transcription factor 1) footprints, for example, were identified exclusively in ectodermal clusters in both mouse and chicken (figs. S21D and S22D). GRHL1 is recognized for its conserved role maintaining the integrity of epithelial tissues, influencing epithelial differentiation and skin barrier formation (*77*). In chicken mesenchymal clusters, particularly the frontonasal mesenchyme, additional TF footprints were detected for HAND2 (heart and neural crest derivatives expressed 2) (fig. S22D). HAND2 has been linked to skeletogenesis in zebrafish and mouse, although its functional role in chicken craniofacial tissues remains to be fully elucidated through targeted experiments (*78*, *79*).

### Genetic variation in craniofacial traits aligns with regulatory elements identified in facial populations

**Fig. 7.**
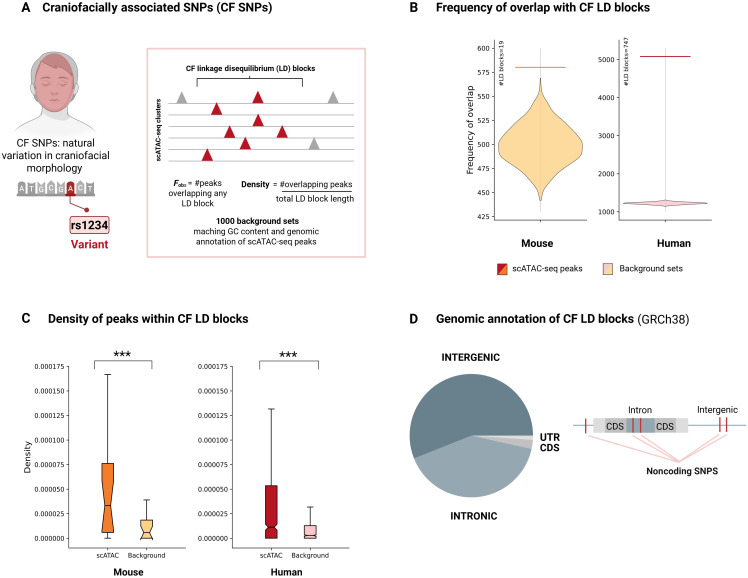
Overlap of scATAC-seq peaks with craniofacial trait–associated LD blocks. (**A**) Overview of craniofacial (CF) SNPs identified in GWAS and the analysis pipeline. (**B**) Violin plots showing the frequency of overlap between scATAC-seq peaks or background sets and LD blocks containing CF SNPs in mouse and human. (**C**) Box plots showing the density of scATAC-seq peaks (darker colors) and background regions (light colors) in LD blocks containing CF SNPs in mouse and human. (**D**) Genomic annotation content of CF LD blocks in the human genome (GRCh38), highlighting their prevalence in noncoding regions. Figure created in BioRender. Kyomen, S. (2026) https://biorender.com/g01q360.

Craniofacial morphology is a highly heritable trait, and genetic studies have begun to uncover loci associated with its natural variation across species. To assess whether regions of open chromatin identified by scATAC-seq might have regulatory roles during craniofacial development, we integrated genome-wide association study (GWAS) data from Pallares *et al.* (*80*), who reported 19 linkage disequilibrium (LD) blocks in the mouse genome associated with variation in craniofacial morphology. The expectation was that if accessible chromatin regions contribute to gene regulation during facial development, they would overlap these GWAS LD blocks more frequently than expected by chance. To test this hypothesis, the number of scATAC-seq peaks overlapping LD blocks was quantified and compared to a null distribution generated from 1000 sets of background peaks matched for GC content and genomic annotation (Fig. 7A). The observed overlap occurred significantly more often than in background sets (*P* < 0.001), supporting the enrichment of craniofacial trait–associated LD blocks within these open chromatin regions (Fig. 7B and table S7). Furthermore, the density of scATAC peaks was significantly higher within LD blocks than in background peaks (*P* < 0.001), reinforcing this conclusion (Fig. 7C and table S7).

Although these findings support a regulatory role for the accessible regions, the mouse GWAS loci reported by Pallares *et al.* (*80*) likely represent only a fraction of the genetic variation influencing craniofacial morphology. In contrast, human GWASs have identified hundreds of loci associated with facial morphology (*81*–*83*). To expand the analysis, human GWAS results were integrated with scATAC-seq data, thereby increasing the number of LD blocks linked to craniofacial traits. This approach provided a stronger framework to test whether accessible chromatin regions in the developing mouse face correspond to regulatory elements under the assumption that at least partial conservation of function exists between mice and humans.

A total of 1687 genome-wide significant single-nucleotide polymorphisms (SNPs) (*P* < 5 × 10^−8^) were compiled from the EMBL (European Molecular Biology Laboratory) GWAS Catalog. Because LD between SNPs and causal variants may produce spurious correlations, analyses were based on LD blocks surrounding significant SNPs rather than the SNPs themselves. SNPs were grouped according to the superpopulation in which the GWAS was performed, and LD blocks were generated following the pipeline described in Materials and Methods. This analysis resulted in a total of 747 LD blocks. Genomic annotation revealed that the majority of sequence within these blocks was noncoding, particularly intronic and intergenic regions, consistent with previous research (Fig. 7D) (*83*).

To calculate the frequency of co-occurrence with human GWAS LD blocks, genomic positions of scATAC peaks and the 1000 background peak sets were converted from mouse to human coordinates using UCSC’s liftOver tool. The frequency of overlap between scATAC peaks and LD blocks was again far greater than expected by chance (*P* < 0.0001), and the peak density within LD blocks was significantly higher than that of background peaks (*P* < 0.001) (Fig. 7, B and C, and table S7).

Together, these results demonstrate that open chromatin regions in facial tissues are nonrandomly distributed within LD blocks linked to facial morphology traits, supporting a functional connection between genetic variation and the regulation of craniofacial development. These findings further suggest the potential evolutionary conservation of regulatory mechanisms underlying craniofacial morphology between mouse and human.

## DISCUSSION

A substantial diversity in facial shapes is observed in birds and mammals, with notable differences in beak and snout geometry, reflecting an array of adaptations and lifestyles. This diversity emerges from tightly regulated developmental processes that control the formation, growth, and fusion of the frontonasal prominences, structures that lay the blueprint for facial morphology. Despite these differences, the core facial developmental organizers and their molecular components are conserved between birds and mammals. Differential gene expression patterns across species have been recognized as a key factor contributing to morphological variation, arising from complex mechanisms involving both conserved and divergent CREs (*28*, *84*). The unique structural organization of these regulatory elements enables them to interpret developmental signaling in a manner that is both stage- and location-specific, playing a crucial role in shaping species-specific traits (*24*, *62*, *85*, *86*). Therefore, we set out to dissect the regulatory and molecular underpinnings in high resolution, specifically focused on the FEZ, a major face-shaping developmental organizer.

The FEZ is conventionally defined as the boundary between mutually exclusive *Shh* and *Fgf8* expression domains in the oral ectoderm (*1*, *12*). We reveal a more complex molecular organization within the FEZ, with *Shh*, *Fgf8*, and *Bmp4* colocalizing in specific regions, challenging the classical model of a strict *Shh*-*Fgf8* boundary. In addition, in developing mouse embryos, *Shh* is produced by a small population of ectodermal cells in the ventral nasal pit rim at the interface between the surface ectoderm and the olfactory epithelium. This *Shh* source complements the previously known *Shh* from the oral ectoderm. In contrast, in chickens, *Shh* is not expressed in the nasal pit rim, contributing to differential gene expression patterns observed between mammals and birds.

Extending a comparative perspective to *A. sagrei* provides a broader framework for understanding how facial morphogenetic signaling is organized across amniotes. Prior work had suggested that early facial morphogenesis in lizards may not strictly conform to the avian FEZ model. This conclusion was largely based on reported spatial expression patterns and the apparent absence of certain localized *SHH* domains, even though experimental perturbations of *Hh* and *FGF* signaling in lizards alter facial morphology in pathway-specific ways (*48*). Our findings indicate that while there are lineage-specific variations, key molecular components of the FEZ are present in lizard, chicken, and mouse facial ectoderm.

First, the partial overlap of *SHH* and *FGF8* expression domains in discrete regions of the oral ectoderm suggests that the spatial relationship between these ligands is more flexible than previously appreciated, with localized zones of coexpression rather than strictly segregated boundaries (figs. S5, S6, and S9). Furthermore, mouse alone displays a small *SHH*-positive domain at the ventral nasal pit rim that is absent in both lizards and birds. This feature likely represents a mammalian novelty rather than a conserved amniote trait. Together, these findings support a model in which the conserved signaling molecules associated with the FEZ are present in developing faces across amniotes, but the precise spatial architecture of these domains is flexible, providing a plausible developmental mechanism for generating facial diversity without requiring strict conservation of FEZ geometry.

By placing FEZ organization within this broader evolutionary context, our work emphasizes that conserved molecular signaling centers can accommodate lineage-specific modifications and that the features such as the nasal pit *SHH* domain in mammals may contribute to the diversity of amniote facial morphologies. We focus the detailed analyses on mouse and chicken because these well-characterized model species allow comprehensive exploration of ectodermal and mesenchymal transcriptomic and regulatory profiles in a mammal and a sauropsid, offering a comparative framework for interpreting patterns observed in other taxa, including lizards. Extending analyses to additional amniote lineages will be essential to determine which aspects of FEZ deployment are broadly conserved versus lineage-restricted, thereby clarifying how conserved signaling modules can underlie both developmental robustness and morphological diversification.

We also observed additional unique expression domains, such as *Wnt5a*, a key regulator of tissue polarity and outgrowth (*87*). In mouse, *Wnt5a* is expressed in the nasal pit rim, whereas in chicken, it is largely restricted to the mesenchyme, indicating differences in the genomic regulation of WNT signaling during facial morphogenesis. These findings support the view that although core signaling pathways are conserved (*4*, *7*), their precise spatiotemporal regulation differs across species and contributes to the diversity of facial morphologies observed in nature. These observations further underscore the importance of conducting comparative genomics in a tissue- and stage-specific manner and highlight the need for more detailed investigation of the gene expression programs controlling facial development.

The regulatory landscapes operating in the developing face are incredibly complex and largely unique to certain clades (*28*). Our results show that only a small percentage of DARs are directly conserved between chicken and mouse, with conservation levels varying between cell types, in agreement with previous comparisons of brain regulatory architecture in these species (*88*). Among functionally conserved DARs, we show that the cis*-*regulatory landscapes of ectodermal populations present higher conservation between chicken and mouse compared to mesenchymal populations. The higher conservation of ectodermal regulatory modules points to preservation of core molecular programs, while greater divergence in mesenchymal regulatory landscapes likely provides flexibility in facial morphogenesis by tuning how mesenchymal cells respond to ectodermal signals. Together, these observations support a central role for cis-regulatory evolution in driving variation in facial shape across amniotes.

We have recovered previously overlooked transcriptomic and regulatory differences among facial prominences and identified cCREs with a cell population–specific activity. It is likely that these population-specific cCREs have gone unnoticed because of the lower-resolution data obtained in traditional whole-embryo screens. In addition to supporting future comparative studies, these data offer a reference framework for clinical investigations of congenital craniofacial disorders. There is increasing evidence that CREs play an important role not only in development and evolution but also in pathological conditions. For instance, in the case of the Pierre Robin syndrome, mutations in a long-range enhancer affect *Sox9* expression in the lower jaw and lead to congenital defects (*31*). Other examples include genetic variants (SNPs) found in CREs linked to abnormalities such as cleft lip and palate (*33*). Our study integrated human GWAS data with chromatin accessibility maps from developing mouse facial tissues to explore the regulatory basis of facial variation. We found that many craniofacial SNPs are located in intronic and intergenic regions, pointing to an association between genetic variation and the regulation of craniofacial development. Moreover, our results indicate that LD blocks associated with facial morphology are enriched for open chromatin regions in facial tissues, suggesting that these genomic intervals may serve as hubs of regulatory activity during craniofacial development.

Our study provides a comprehensive analysis of the molecular architecture and regulatory landscapes of the developmental organizer that drives facial development in mouse and chicken. However, certain limitations should be acknowledged. Our analysis focuses on a specific developmental window in mouse and chicken, where the main face-shaping developmental organizer FEZ demonstrated the peak of signaling activity. Expanding the analyses to additional stages, particularly in chicken, would offer a more complete view of regulatory dynamics. While we identified putative CREs, mapped their activity, and compared them to the respective gene expression patterns, targeted functional validation, such as CRISPR deletions, will ultimately confirm their exact role in the complex developmental process. This study focused primarily on in vivo mapping of murine cCREs. Future validations of cCREs identified in chicken will allow us to understand their tissue specificity and determine whether chicken regulatory elements operate in homologous areas of the developing face as in mouse or account for establishing divergent gene expression patterns. Last, with future technological advances allowing spatial profiling of open chromatin landscapes, it will be possible to refine cell type– and region-specific regulatory landscapes.

In summary, this study uncovers how cis-regulatory evolution rewires conserved developmental programs to generate the extraordinary diversity of vertebrate facial forms. By resolving regulatory landscapes at a single-cell resolution in mouse and chicken, we reveal that mesenchymal populations represent a key source of regulatory divergence. By examining the regulatory landscapes of individual cell populations in mouse and chicken, conserved CREs were shown to guide core face development, while divergent elements contribute to the emergence of distinct facial shapes. In addition, we show that the ectodermal developmental organizer exhibits unexpected molecular complexity, with the mouse-specific *Shh/Wnt5a* expression domain present in the murine nasal pit rim. Together, these findings establish a solid framework and information-rich resource for investigation of morphological evolution of the facial shape in mammals and birds. The single-cell transcriptomic and open chromatin data presented in this study, together with the associated microscopy images, can be explored in a user-friendly interactive online database (www.evolbio.mpg.de/kyomen-cis-regulatory-evolution).

## MATERIALS AND METHODS

### Animal husbandry

#### Chicken

Freshly laid fertilized chicken eggs were obtained from Lohmann Breeders GmbH & Co (Ankum, Germany) and placed in a rotating incubator set at 37°C with controlled humidity for a duration of 3.5 to 4 days at the Max Planck Institute for Evolutionary Biology, Plön, Germany. Embryos were collected, staged according to Hamburger and Hamilton (*89*), and placed in ice-cold phosphate-buffered saline (PBS) before dissection. All procedures were performed on chicken embryos before 13 days of embryonic development and therefore fall outside the scope of German and European animal welfare regulations such that no specific ethical approval was required. Short-term housing of fertilized eggs was approved by the local veterinary authority.

#### Mice

All experiments were performed in strict accordance with the legal requirements of the Local Ethics Committee Germany (Kiel, Germany), the Federation of European Laboratory Animal Science Associations, and the German animal welfare law (Tierschutzgesetz §11). C57BL/6 mice were obtained from Charles River Laboratories (Germany; strain code 632) and housed at Max Planck Institute for Evolutionary Biology’s Animal Facility. Housing and breeding were approved by the MLLEV (Ministerium für Landwirtschaft, ländliche Räume, Europa und Verbraucherschutz des Landes Schleswig-Holstein) under permit PLÖ-0004697. Animals were housed in ventilated cages with food and water ad libitum. Mating pairs were set overnight, and the presence of a vaginal plug was assessed on the next day, which was considered E0.5. Pregnant females were euthanized by cervical dislocation. E11.5 embryos (45 to 47 somites) were carefully dissected out, placed in ice-cold PBS, and microdissected.

### Sample preparation for single-cell omics

Frontonasal prominences were carefully dissected from freshly collected mouse E11.5 and chicken HH22 embryos using sharp forceps. For both single-cell technologies, facial prominences from at least 30 embryos were pooled. The following procedure was applied to both species independently. Dissected facial tissues were placed into 1.5-ml Eppendorf tubes containing ice-cold PBS. The samples were centrifuged at 500*g* for 5 min at +4°C. After carefully removing the PBS, 1 ml of prewarmed trypsin solution (0.05% trypsin/0.02% EDTA, Pan Biotech, P10-0235SP) was added to each tube and incubated in a thermomixer at 37°C for 15 min with gentle agitation. To ensure thorough mixing, the samples were gently vortexed for 30 s every 5 min during the incubation period. Following incubation, tissue dissociation was carried out by carefully pipetting the samples up and down for 15 s. Once the cell suspension appeared homogeneous, 200 μl of prewarmed fetal bovine serum was added to the samples to quench the enzymatic reaction. After another round of centrifugation at 500*g* for 5 min at +4°C, the supernatant was carefully removed without disrupting the pellet. The cells were then resuspended in 1 ml of cold PBS and 2.5 μl of ribonuclease inhibitor (Thermo Fisher Scientific, N8080119) and spun down at 500*g* for 5 min at +4°C. The washing step was repeated three more times to remove any excess trypsin and cell debris. The resulting suspension was subsequently filtered through a 35-μm cell strainer (Falcon, 352235). Assessment of cell viability and concentration was performed by adding a 1:1 proportion of 0.4% Trypan blue dye (Gibco, 15250061) to an aliquot of the cell suspension. Cells were loaded in C-Chip hemocytometer slides (NanoEnTek, DHC-N01) and assessed using a stereomicroscope. When the cell viability was at least 95%, the cell suspension was diluted in nuclease-free water to contain 1200 cells/μl, ensuring optimal conditions for downstream processes and aligned with the experimental requirements outlined by the 10x Genomics protocol, targeting 10,000 cells.

For scATAC-seq, the nucleus isolation protocol was executed following the guidelines of 10x Genomics. After dissociation, cells were centrifuged at 500*g* for 5 min at +4°C. The supernatant was removed, and the cell pellet was incubated in 100 μl of cold lysis buffer (10 mM tris-HCl, 10 mM NaCl, 3 mM MgCl_2_, 0.1% Tween 20, 0.1% Nonidet P40 Substitute, and 1% bovine serum albumin) for 5 min on ice. After incubation, 500 μl of wash buffer (10 mM tris-HCl, 10 mM NaCl, 3 mM MgCl_2_, 0.1% Tween 20, and 1% bovine serum albumin) was added. The samples were then centrifuged at 500*g* for 5 min at +4°C, and the resulting nuclei pellet was resuspended in Diluted Nuclei Buffer (10x Genomics Nuclei Buffer; 2000153). Nucleus concentration and viability were assessed using the same procedures as for the scRNA-seq. The nucleus concentration was appropriately adjusted to achieve the desired input for tagmentation.

### scRNA-seq: GEM generation, library preparation, and sequencing

The scRNA-seq library was generated following the 10x Genomics protocol designed for the Chromium Next GEM Single Cell 3′ Reagent Kits v3. Briefly, the Chromium Next GEM Chip G was loaded with a mixture of the final cell suspension and Master Mix (RT Reagent B 2000165, Template Switch Oligo 3000228, Reducing Agent B 2000087, and RT Enzyme C 2000085/2000102). The remaining rows were allocated for Single Cell 3′ v3.1 Gel Beads (2000164) and partitioning oil, with one row left unfilled. Any unused wells were filled with a 50% glycerol solution.

Upon the conclusion of the run, the Gel Beads-in-emulsion (GEMs) were recovered from the chip and placed into a PCR (polymerase chain reaction) tube on ice. GEMs were subsequently incubated in a thermal cycler for 55 min (53°C for 45 s, followed by 85°C for 5 s). The GEM cleanup procedure was performed according to the manufacturer’s instructions. Postcleanup, a recovered sample volume of 35 μl was combined with the cDNA Amplification Reaction Mix. Subsequently, an additional round of incubation was conducted, following the settings described in the cDNA Amplification process outlined in the manufacturer’s 10x Genomics protocol, for a total of 13 cycles. Last, the sample underwent another cleanup phase using SPRIselect (Beckman Coulter SPRIselect Reagent Kit, B23318), after which the quantification of cDNA was assessed using Invitrogen Qubit dsDNA assay kits and a Qubit fluorometer (Thermo Fisher Scientific, 10616763). Libraries were sequenced with paired-end 150-bp reads, targeting 50,000 reads per nucleus on the Illumina NovaSeq 6000.

### scATAC-seq: GEM generation, library preparation, and sequencing

The scATAC-seq libraries were generated in accordance with the recommended protocol provided by 10x Genomics (Chromium Single Cell ATAC). In summary, 5 μl of resuspended nucleus dilution was combined with 10 μl of Transposition Mix to yield a final volume of 15 μl. These samples were then subjected to an incubation step in a thermal cycler at 37°C for 30 min. GEM generation was performed using the Chromium Next GEM Chip H, following the protocol of the manufacturer. After the incubation, 40 μl of eluted DNA was retrieved using the magnetic separator. An additional cleanup step was conducted using the SPRIselect reagent (Beckman Coulter SPRIselect Reagent Kit B23318).

For library preparation, each sample was supplemented with 57.5 μl of Sample Index PCR Mix along with 2.5 μl of individual Single Index N Set A (3000427). These samples were then incubated following the 10x Genomics protocol for a specified number of cycles that match the targeted nucleus recovery. Subsequent to amplification, fragment size selection was carried out until a recovered sample volume of 20 μl was obtained. DNA was quantified using the Invitrogen Qubit dsDNA assay kit and a Qubit fluorometer (Thermo Fisher Scientific, 10616763). The resulting libraries were stored at −20°C until sequencing. Libraries were sequenced with paired-end 50-bp reads, targeting 50,000 reads per nucleus on the Illumina NovaSeq 6000 S1.

### ChIP-seq: Chicken (HH22)

We performed ChIP-seq for H3K27ac, a known enhancer-associated histone modification. Briefly, frozen frontonasal tissue of chicken embryos (HH22) was pulverized with a mortar and pestle, resuspended in PBS, and cross-linked with 1% formaldehyde at room temperature for 10 min. Chromatin was sonicated to generate fragments averaging 200 to 600 bp in size. Chromatin was incubated for 3 hours at 4°C with 10 μg of Cell Signaling H3K27ac antibody (8173S; lot: 106590037-6). Protein A and G Dynabeads (Invitrogen) were then added to this chromatin/antibody mixture for 30 min at 4°C. Immunocomplexes were sequentially washed, and protein/DNA complexes were eluted in an SDS buffer (1% SDS, 50 mM tris, pH 8.0, and 10 mM EDTA) at 37°C for 1 hour. Samples were treated with 1 μl of proteinase K (20 mg/ml) at 65°C and reverse cross-linked overnight. Last, the DNA was purified using Nucleic Acid Extraction Reagent (Y202-G30), and the quality was assessed on the Agilent Bioanalyzer. The ChIP-seq libraries were prepared using the AcceGen DNA Library Prep Kit from Illumina (AG1216). Libraries and input samples were pooled and sequenced with paired-end 150-bp reads on the Illumina NovaSeq 6000.

### Quantification and statistical analysis

#### scRNA-seq: Chicken (HH22)

The 10x Genomics Cell Ranger (7.1.0) pipeline was used to process the chicken RNA-seq data. First, a custom chicken reference was constructed on the basis of the galGal6 assembly (GenBank assembly GCA_000002315.5) using “cellranger mkref.” The reads were then aligned to this reference and quantified with “cellranger count.” After alignment, quality check and downstream analyses were performed in Seurat (4.3.0). Low-quality cells containing extensive mitochondrial counts, low gene counts, and doublets were filtered out. The data were then normalized and regressed out for cell cycle heterogeneity. After scaling, linear dimensional reduction with principal components analysis was performed on variable genes. Cells were then subjected to unbiased clustering at a resolution of 0.8. For two-dimensional visualization of clusters, UMAP was applied to principal component embeddings. Cell clusters were then annotated on the basis of the output of the function FindAllMarkers (Wilcoxon rank-sum test, min.pct = 0.25, log-change threshold = 0.25) alongside manual inspection of expression profiles of known cell-specific markers.

#### scRNA-seq: Mouse (E11.5)

The mouse scRNA-seq dataset analyzed in this study was derived from an scRNA-seq experiment conducted on E11.5 facial tissue, encompassing the lateral nasal, medial nasal, and maxillary prominences. The dataset is part of a manuscript under consideration. Facial prominences were dissected and dissociated following the protocol outlined earlier. The experiment used the Chromium Next GEM Single Cell 3′ Reagent Kits v3, adhering to the manufacturer’s standard protocol. Sequencing data were aligned using Cell Ranger (version 7.1.0) with the prebuilt *Mus musculus* reference genome (mm10) provided by 10x Genomics. Quality control and initial analyses were performed in Scanpy (version 1.9.3), with doublet detection executed using Scrublet (*90*). Doublet thresholds were determined on the basis of the multiplet rate table from 10x Genomics, and identified doublets were excluded. Quality control metrics—including total counts, unique gene counts, mitochondrial gene content, and ribosomal gene content—were calculated using Scanpy’s “pp.calculate_qc_metrics” function. Cells not meeting the following thresholds were excluded: total counts (12,000 to 50,000), unique gene counts (3200 to 8000), mitochondrial content (1.9 to 5.2%), and ribosomal content (15 to 33%). For cell type annotation, the expression matrix was normalized using “pp.normalize_total” and log transformed via “pp.log1p.” Highly variable genes were identified with “pp.highly_variable_genes,” followed by principal components analysis for initial dimensionality reduction. Cells were visualized in a two-dimensional space using the UMAP algorithm, clustered using “tl.leiden,” and annotated on the basis of established marker genes. Mesenchymal and ectodermal cells were subset, and the same analysis pipeline was applied to refine their annotation. Further analysis was conducted in Seurat after exporting the dataset to R. To achieve equal representation of cell clusters across RNA-seq and ATAC-seq experiments, the mouse scRNA-seq dataset was subset to exclude clusters associated with maxillary prominences. Subsequent analyses mirrored the steps applied to the chicken scRNA-seq dataset, ensuring consistency in methodology across species.

#### Mouse-chicken scRNA-seq joint embedding

Joint embedding of mouse and chicken scRNA-seq datasets was generated using the SAMap algorithm (version 1.0.15) (*42*). First, gene-gene bipartite maps of each species and their respective transcriptome were constructed by reciprocal BLAST using blastp following the map_genes.sh script available at SAMap GitHub repository (https://github.com/atarashansky/SAMap/). Seurat objects were then converted to .h5ad format using SeuratDisk’s “convert.” A SAMap object was then built with input of mouse and chicken scRNA-seq .h5ad files alongside the gene map output of map_genes.sh. Dataset integration was performed using the “SAMAP.run” function. For visualization, we computed a cross-species UMAP with a subset of 3000 cells from each species. Sankey plots using the ggalluvial package in R were generated using the full datasets, with a minimum alignment score of 0.2 for paired clusters.

#### Cell-cell cross-talk network analysis

To infer cell-cell communication among clusters in the scRNA-seq datasets, we used the CellChat package (version 2.1.0), which reconstructs intercellular signaling networks by integrating known ligand-receptor interactions with transcriptomic data (*43*). CellChat uses a curated database of ligand-receptor pairs and multisubunit complexes and infers cell-cell communication by quantifying the probability and strength of signaling between groups of cells on the basis of the differential expression of ligands in sender populations and their corresponding receptors in receiver populations. Because our dataset includes chicken cells and the built-in CellChat databases are based on mouse and human interactions, we first mapped mouse-chicken orthologs using a homology graph generated by SAMap. For each gene, only the top orthologous match was retained to avoid ambiguous one-to-many or many-to-one mappings, and genes lacking any ortholog were excluded from downstream CellChat analysis in both species. This ensured that ligand-receptor pairs and signaling pathways inferred by CellChat were based on comparable gene identities across both datasets. Processed single-cell gene expression matrices in Seurat objects were exported to CellChat, and the working ligand-receptor database was set to CellChatDB.mouse for both chicken and mouse datasets. Cell-cell communication was then inferred using the “computeCommunProbPathway” function, which considers differential expression of ligands and receptors across cell groups (*P* < 0.05). Signaling network strength and pathway contributions were further quantified using CellChat’s built-in aggregation functions.

#### scATAC-seq: Preprocessing, dimensionality reduction, and clustering

Raw sequencing reads were processed using the Cell Ranger ATAC pipeline (version 2.1.0). Base calls were demultiplexed with the “cellranger-atac mkfastq” function. For chicken, a custom reference genome was created using “cellranger-atac mkref” on the basis of the galGal6 assembly (GenBank assembly: GCA_000002315.5). For the mouse dataset, the precomputed mm10 reference from 10x Genomics was used. Sequencing reads were aligned to their respective references with the “cellranger-atac count” function. Subsequent analyses used the Analysis of Regulatory Chromatin in R (ArchR) package (version 1.0.2) (*51*). The Cell Ranger ATAC outputs served as inputs to generate Arrow files, containing the relevant dataset information. For chicken, a custom ArchR reference was built using the galGal6 assembly, with sex chromosomes (M and W) and gene annotations beginning with “LA_,” “LOC,” and “MIR” excluded. For the mouse, the preexisting mm10 ArchR reference was used. Initial filtering parameters, including transcription start site enrichment scores, fragment size distribution, and doublet removal, are detailed in the Supplementary Materials. Dimensionality reduction was achieved using latent semantic indexing (LSI), a method optimized for addressing the sparsity inherent in scATAC-seq datasets. The iterative LSI process was applied via the ArchR “addIterativeLSI” function. Clustering was performed with the ArchR “addClusters” function using Seurat as the method with default parameters. UMAP embeddings were generated with the “addUMAP,” specifying 30 nearest neighbors. Resolutions were set to 0.8 for the mouse dataset and 0.4 for the chicken dataset. Final UMAP visualizations were produced using the “plotEmbedding” function with default parameters.

#### scATAC-seq: Peak calling, identification of DARs, and gene scores

First, pseudobulk replicates were created by applying ArchR’s function “addGroupCoverages.” Peak calling was performed using MACS2 (Model-based Analysis for ChIP-Seq 2) by applying the function “addReproduciblePeakSet” (*91*). A new matrix was then created with insertion counts derived from the identified peaks using the “addPeakMatrix” function. To calculate the DARs, the “getMarkerFeatures” function was applied with the peak matrix as input and cutoff of false discovery rate (FDR) <= 0.01 and Log2FC >= 1.25. Gene activity scores for each cell group were calculated by the “addGeneScoreMatrix” function. Cluster-specific genes based on gene scores were identified by applying the “getMarkerFeatures” function with the gene score matrix as input (FDR <= 0.01 and Log2FC >= 0.8). Transcription start site enrichment and the number of unique fragments per cell were used as bias. To improve the visual interpretation of gene scores, the MAGIC (Markov Affinity-based Graph Imputation of Cells) algorithm was applied by ArchR’s “addImputeWeights” function (*92*). Then, heatmaps of marker genes and marker peaks per clusters were obtained via “plotMarkerHeatmap.”

#### scATAC-seq: Motif enrichment analysis and TF footprinting

Motif enrichment was performed on DARs using ArchR’s function “peakAnnoEnrichment,” applying a cutoff of FDR <= 0.05 and Log2FC >= 1. Motif annotation was applied using “addMotifAnnotations” with the default parameters. For the mouse and chicken datasets, motif annotation was based on JASPAR 2022 core vertebrate motifs. To generate TF footprints, the same pseudobulk ATAC profiles used during peak calling were used as input to the “getFootprints” function, using Tn5 bias subtraction as the normalization method.

#### Integration of scATAC-seq with scRNA-seq datasets and peak-to-gene links

Unconstrained integration of scRNA-seq and scATAC-seq data was performed using the function “addGeneIntegrationMatrix” from ArchR. In this function, the scRNA-seq gene expression matrix is aligned to the scATAC-seq gene score matrix, so each cell in ATAC has a gene expression signature. Integration labels were then used to assess scATAC-seq cluster annotation and perform peak-to-gene linkage analysis. Using the function “addPeak2GeneLinks,” we calculated the correlation between chromatin accessibility and gene expression. Last, we identified the significant peak-to-gene links with the following cutoff of correlation >0.4 and RNA-seq correlation >0.7.

#### Interspecies point projection (IPP)

Genomic regions of interest were mapped between mouse and chicken genomes using IPP, a method that infers evolutionary conservation beyond direct sequence alignment (*56*). IPP requires pairwise alignment files linking the reference, query, and bridge genomes; these files were generated using the genome assemblies listed in table S4. The IPP algorithm was then executed with the default parameters described in the original publication. Mouse and chicken peaks were projected reciprocally between species and through each bridging genome. Genomic regions were classified as directly conserved (projection score >0.98), indirectly conserved (score >0.84), or nonconserved (score <0.84). Conservation proportions were then evaluated in both projection directions (mouse-to-chicken and chicken-to-mouse). Resulting projection outputs for DARs from both mouse and chicken and in vivo enhancers are provided in table S4.

#### Hi-C

Hi-C contact maps of facial prominences from E11.5 embryos were obtained from National Center for Biotechnology Information (NCBI) Gene Expression Omnibus (GEO) depository under accession number GSM5944421 (*63*). The .hic file was imported to Juicebox Web App (*93*) for visualization with balanced normalization. TADs were identified using the Arrowhead algorithm included in Juicer Tools version 2.20.00. Domain calling was performed at multiple resolutions: 50, 25, and 10 kb, with Knight-Ruiz–normalized matrices. The output files were uploaded to Juicebox Web App as two-dimensional annotation tracks. Domain boundaries were inspected in the context of the contact matrix, focusing on regions of interest.

#### Gene ontology

Gene ontology terms associated with conserved DARs from ectodermal and mesenchymal clusters were obtained by applying GREAT (*57*). The whole-mouse genome (mm10) was used as background. The analysis was performed following the default parameters (5 kb upstream and 1 kb downstream basal and extension for proximal regulatory regions, up to 1000 kb for distal regions).

#### ChIP-seq: Chicken (HH22) data analysis

Initial quality control was performed by trimming adapter sequences from the raw FASTQ files of two biological replicates, along with their corresponding input controls, using cutadapt (version 1.8.3), ensuring that only high-quality reads were retained for alignment. The trimmed paired-end reads were then aligned to the chicken genome (galgal6) using Bowtie2 (version 2.2.4) with default parameters. The alignment process produced SAM files, which were subsequently converted to sorted BAM files using samtools. Peak calling was performed with MACS2 using a *q*-value threshold of 0.05 and an extension size of 200 bp, applying the “--nomodel” option. Only overlapping peaks from both replicates were considered to ensure reliable and reproducible results. These peaks were then annotated using ChIPseeker (version 1.2.6) to identify associated genomic features, such as promoters and exons.

#### Identification of candidate CREs

Genomic regions were assigned as cCREs if conforming to at least three of the following criteria: peak annotated as intronic, distal, or intergenic; the peak-to-gene links between the peak and its associated gene have correlation higher than 0.4; the presence of ENCODE registry of cCREs with evidence of H3K27ac marks; in vivo–validated enhancer activity; assessed conservation using UCSC phyloP scores (60 species and placentals); and overlapping peak in publicly available ChIP-seq targeting H3K27ac histone modification. Single-cell expression readouts of nearby genes were also taken into consideration. The list of in vivo–validated enhancers from the VISTA database that intersect peaks in our scATAC-seq dataset is provided in table S7.

#### Chromatin accessibility around craniofacial morphology–associated SNPs

Mouse craniofacial GWAS LD blocks were obtained from Pallares *et al.* (*80*) and converted from mm8 to mm10 using the UCSC LiftOver tool. To assess whether the overlap between GWAS LD blocks and scATAC-seq peaks could occur by random chance, 1000 sets of background regions were generated to match the distribution of GC content and genomic annotations [intergenic, exon, intron, promoter, and 5′/3′UTR (5′/3′ untranslated region)] of the scATAC-seq peaks. To do this, the mouse genome (mm10) was first tiled into 500-bp segments, which were annotated and analyzed for GC content. Then, background regions were sampled using stratified sampling without replacement via the “matchRegions” function from the nullranges package.

Enrichment of scATAC peaks was tested in two complementary ways in linkage blocks. First, we looked at the frequency of overlap between scATAC peaks and linkage blocks to see if our scATAC peaks were more likely to fall within a linkage block than expected by random chance. The frequency of overlap was defined as the number of scATAC-seq peaks that intersected, either partially or fully, with any LD block. Statistical significance was determined by comparing the observed overlap to the distribution from the 1000 background sets; the empirical *P* value was defined as the proportion of background sets with an equal or greater overlap than the observed value. Next, we tested to see whether the density of scATAC peaks in linkage blocks was higher than would be expected by random chance. For this, we tested for a difference in scATAC peak density in the linkage blocks using a permutation test, with one of the background sets of peaks acting as a control distribution.

To expand the number of genomic regions linked to craniofacial morphology, we integrated human GWAS results across 1794 studies. SNPs associated with facial morphology traits were downloaded from the EMBL GWAS Catalog (www.ebi.ac.uk/gwas/), specifically selecting the “facial morphology trait” dataset mapped to the GRCh38 human genome assembly, comprising a total of 2400 SNPs. These variants were filtered to retain only those reaching genome-wide significance (*P* < 5 × 10^−8^), resulting in 1687 SNPs. SNPs were then grouped according to the superpopulation in which the GWAS was performed: American mixed race (AMR), African (AFR), East Asian (EAS), European (EUR), and South Asian (SAS).

Linkage information for these SNPs was obtained from phase 3 of the 1000 Genomes Project (hereafter 1KG), which includes whole-genome sequencing data from 2504 individuals aligned to the GRCh38 assembly (*94*). Phased VCFs were downloaded and subset to retain only phased SNPs passing quality control filters. Linkage blocks were defined by calculating the LD within the corresponding superpopulation between all 1KG SNPs in a 500-kb window around the genomic position of each significant GWAS SNP. As not all GWAS SNPs were present in the 1KG VCF, we used the closest upstream and downstream 1KG SNPs to act as proxies. The linkage block around each GWAS SNP was then taken as the most upstream and downstream 1KG SNP with an *r*^2^ greater or equal to 0.8 to either of the proxy 1KG SNPs. This was done for all GWAS SNPs. Any overlapping linkage blocks were merged together, resulting in 747 linkage blocks. Enrichment was tested as before for the mouse LD blocks; however, this time, both the original scATAC-seq peaks and control regions were then converted from mm10 to the human GRCh38 assembly using the UCSC LiftOver tool.

### LacZ transgenic mice

Four elements from the list of mouse cCREs and one chicken ortholog were selected for in vivo validation on the basis of their cell type specificity, overlap with H3K27ac ChIP-seq, proximity to target genes, and lack of existing experimental data. The transgenic enhancer-reporter protocol was performed using the enSERT transgenesis technique, following the method outlined by Osterwalder and colleagues (*95*). Each candidate enhancer’s sequence was synthesized by Twist Biosciences and cloned into the pCR4-*Shh*::lacZ-H11 vector (Addgene, no. 139098), which includes the mouse *Shh* minimal promoter, a lacZ reporter gene, and H11 safe harbor locus homology arms. The cloned construct, along with Cas9 protein and H11 single guide RNAs, was injected into mouse embryonic pronuclei (FVB) and transferred to pseudopregnant female hosts (CD-1). Embryos were harvested at E11.5, fixed in 4% paraformaldehyde, and stained for β-galactosidase activity. Only embryos with successful transgene integration at the H11 locus and at the correct developmental stage were analyzed for reporter gene activity. Specific enhancer coordinates are provided in table S6.

### In situ HCR

Dissected mouse (E9.5 to 11.5), chicken (HH16, HH20, HH22, HH24, and HH27), and lizard embryos (stages 3 to 6; provided by N. Feiner) were briefly washed in ice-cold PBS and fixed in freshly prepared 4% paraformaldehyde (paraformaldehyde in PBS) solution overnight at +4°C with gentle rotation. Embryos were then washed with 0.1% PBS-Tween (Tween 20, Sigma-Aldrich, P9416), dehydrated in a graded methanol series (25%-50%-75%-100%) in 0.1% PBS-Tween, and stored at −20°C in glass vials for further use.

The whole-mount HCR followed a modified protocol from Molecular Instruments Inc. and was applied to at least three embryos per stage and probe combination. In summary, fixed embryos were subjected to overnight bleaching in Dent’s Bleach solution (2:1 Dent’s Fix solution, Vaprox-Steris, PB006EUR) at +4°C to mitigate autofluorescence. After bleaching, embryos were briefly washed with 100% methanol and incubated overnight at 4°C in Dent’s Fix solution (4:1 methanol:dimethyl sulfoxide) with gentle rotation. Embryos were rehydrated in a methanol series (100%-75%-50%-25%-0%) diluted in 0.1% PBS-Tween and postfixed using 4% paraformaldehyde for 20 min at room temperature. Embryos were incubated overnight at 37°C in a solution containing 2 pmol of diluted probes in a hybridization buffer (Molecular Instruments, Inc). After the hybridization step, a series of four washes with wash buffer (Molecular Instruments Inc.) was performed to eliminate unbonded probes. Subsequently, embryos were incubated in a solution containing 30 pmol of hairpins H1 and H2 diluted in an amplification buffer (Molecular Instruments Inc.) overnight at room temperature, protected from light. After a wash in 0.1% 5×-SSC-Tween (20× SSC; Fisher Bioreagents, BP1325-4), embryos were incubated in 1× DAPI (4′,6-diamidino-2-phenylindole) solution (Thermo Fisher Scientific, D21490) overnight at 4°C on slow rotation. Samples were cleared with BABB (benzyl alcohol and benzyl benzoate) following a modified protocol described by Becker *et al.* (*96*). Briefly, embryos underwent a 3-min/grade dehydration in increasing methanol series (25%-50%-75%-100%) and were subsequently placed in BABB (*96*) solution until the tissues appeared transparent. Samples were scanned using a Zeiss LSM980 with Airyscan2 confocal microscope, with d Plan-Apochromat 10×/0.45 M27 (Zeiss 420640-9900) objective. All images were exported from ZEN 3.9 software. Probe set information can be found in table S3.

## Supplementary Materials

### The PDF file includes:

Figs. S1 to S22 Tables S1 and S2 Legends for tables S3 to S7 References
