# Supplementary material for: Cis-regulatory evolution shapes facial diversity in birds and mammals
Source: Sci Adv. 2026 May 6;12(19):eaec2511. doi: 10.1126/sciadv.aec2511 (PMC13148318; doi:10.1126/sciadv.aec2511)
Supplement: Supplementary file 1 — Figs. S1 to S22 Tables S1 and S2 Legends for tables S3 to S7 References [file sciadv.aec2511_sm.pdf]

Science Advances
AAAS

Supplementary Materials for

Cis-regulatory evolution shapes facial diversity in birds and mammals

Stella Kyomen *et al.*

Corresponding author: Markéta Kaucká, kaucka@evolbio.mpg.de

Sci. Adv. **12**, eaec2511 (2026)
DOI: 10.1126/sciadv.aec2511

The PDF file includes:

Figs. S1 to S22
Tables S1 and S2
Legends for tables S3 to S7
References

Other Supplementary Material for this manuscript includes the following:

Tables S3 to S7

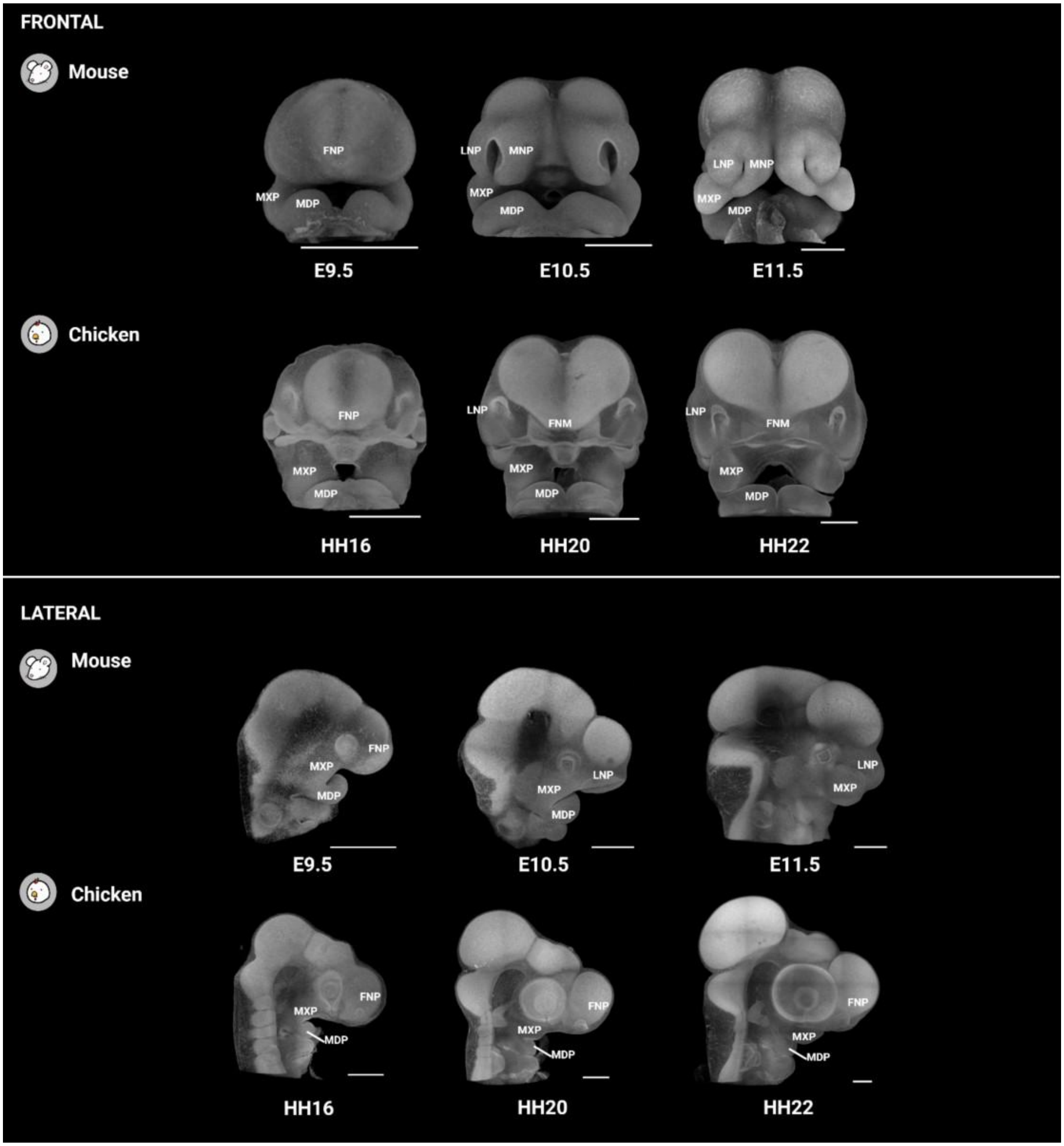

Figure S1. Early facial development in mouse and chicken. Frontal views of mouse (E9.5-E11.5) and chicken (HH16-HH22) embryos. Note that in mice, the lateral nasal prominences (LNP) and medial nasal prominences (MNP) are pronounced, bulging prominently around the nasal pits. In contrast, chicken embryos exhibit a relatively flat nasal region, with facial prominences expanding more laterally. Scale bars: 500 µm. *Abbreviations:* FNP, frontonasal process; MXP, maxillary prominence; MDP, mandibular prominence; LNP, lateral nasal

prominence; MNP, medial nasal prominence; FNM, frontonasal mass. Figure created in BioRender. Kyomen, S. (2026) https://BioRender.com/g01q360

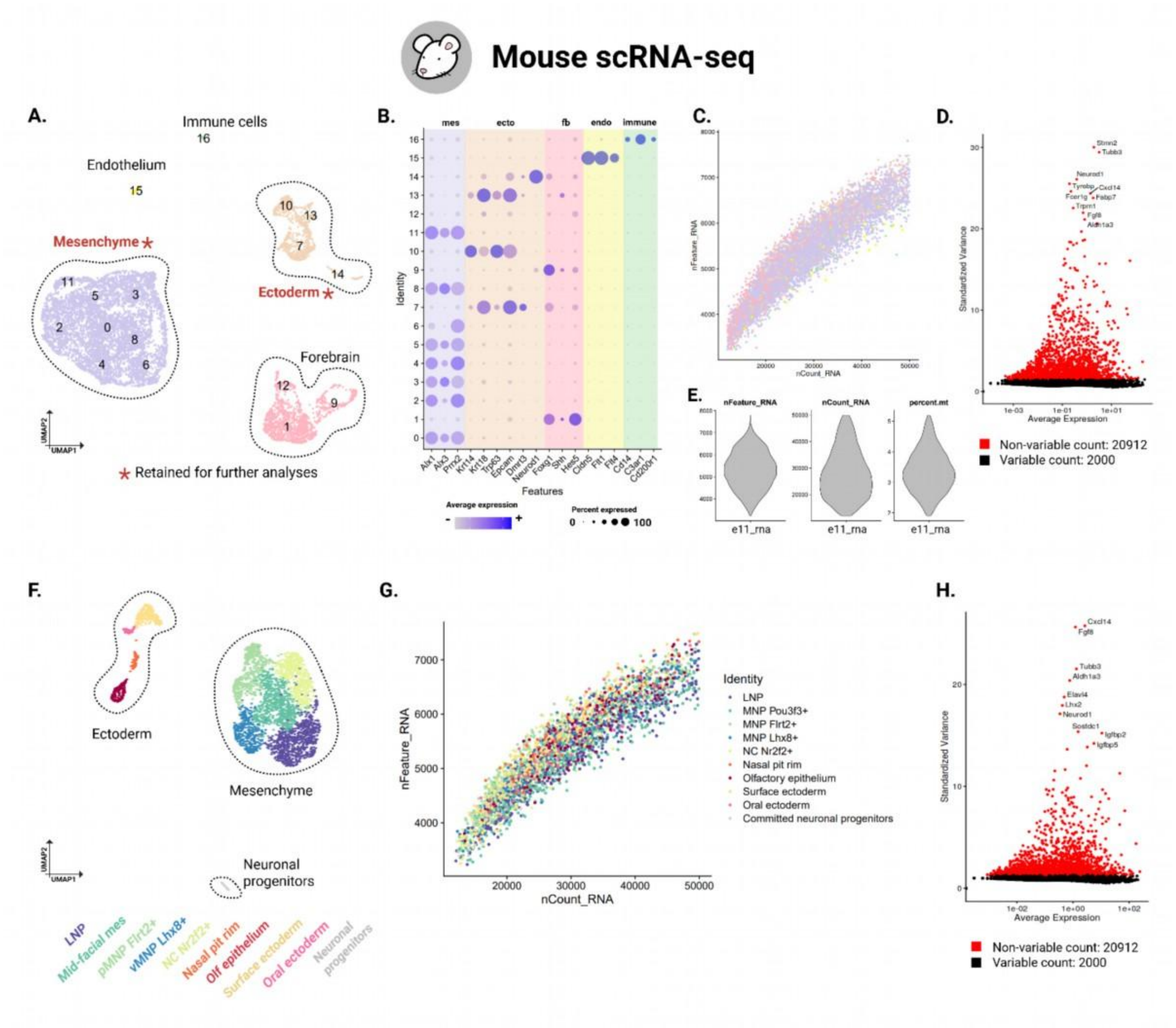

Figure S2. Quality control of mouse scRNA-seq data. A. UMAP showing initial clustering of mouse scRNA-seq data. Only mesenchymal and ectodermal clusters were retained for downstream analyses. **B.** Dot plot illustrating selected marker genes used to annotate major cell populations in the scRNA-seq dataset. **C.** Scatter plot of number of features versus number of counts in the initial clustering, colored by cluster identity. **D.** Selection of the top 2,000 highly variable genes across cells based on initial clustering. **E.** Violin plots showing the distribution of feature counts, total counts, and mitochondrial gene percentages. **F.** UMAP showing refined clustering of facial cell populations from the scRNA-seq dataset, excluding endothelium, blood, immune, and forebrain-derived cells. **G.** Scatter plot of number of features versus number of counts for the final clustering of facial populations, colored by cluster. **H.** Screening of the top 2,000 highly variable genes across cells based on the facial subset of the mouse scRNA-seq data. *Abbreviations:* mes, mesenchyme;

ecto, ectoderm; fb, forebrain; endo, endothelium; immune, immune cells; LNP, lateral nasal prominence; mid-facial mes, mid-facial mesenchyme; vMNP, ventral medial nasal prominence; pMNP, posterior medial nasal prominence; NC, nasal cavity mesenchyme; olf epithelium, olfactory epithelium. Figure created in BioRender. Kyomen, S. (2026) https://BioRender.com/g01q360

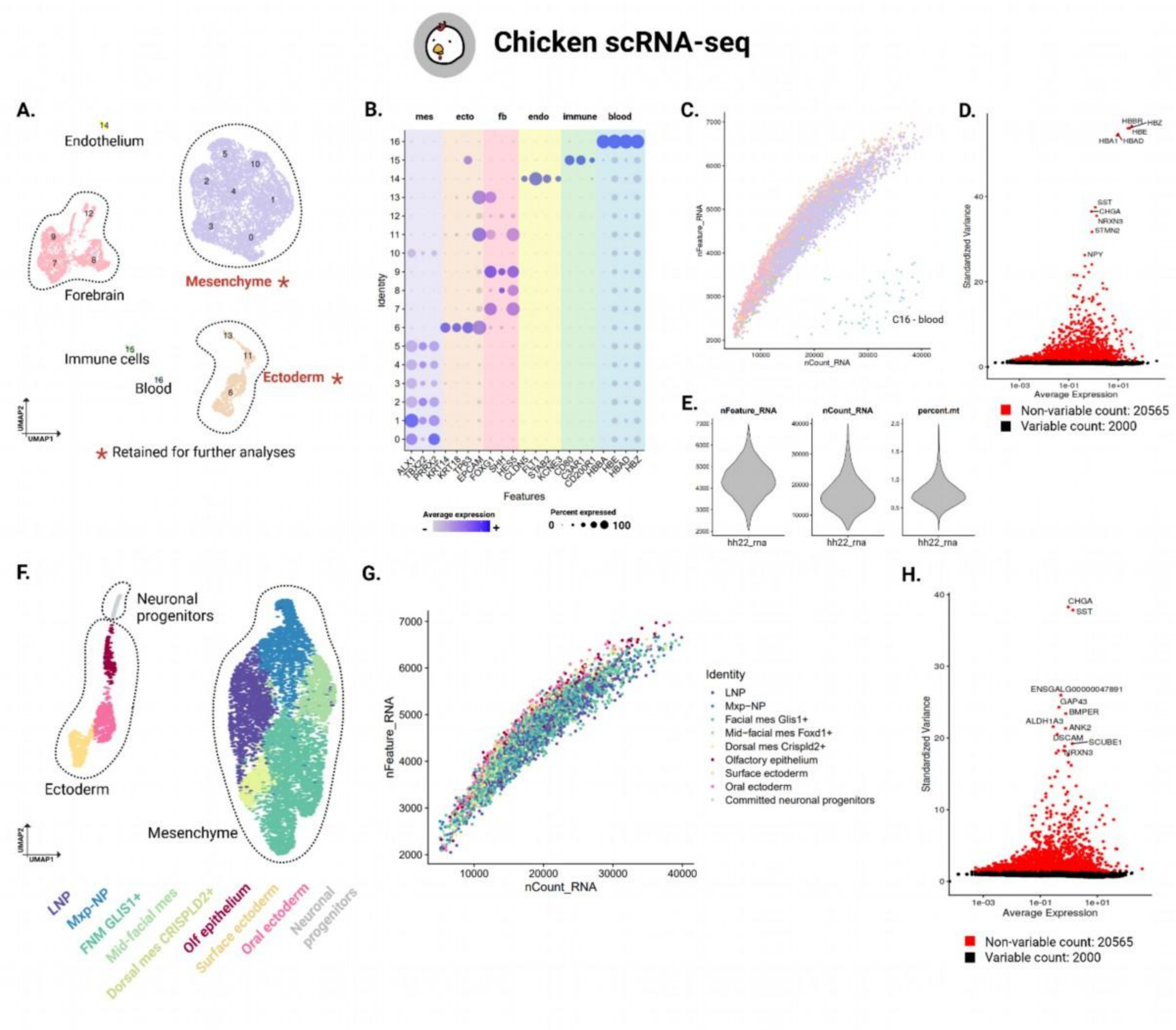

Figure S3. Quality control of chicken scRNA-seq data. UMAP showing initial clustering of chicken scRNA-seq data. Only mesenchymal and ectodermal clusters were retained for downstream analyses. **B.** Dot plot illustrating selected marker genes used to annotate major cell populations in the scRNA-seq dataset. **C.** Scatter plot of number of features versus number of counts in the initial clustering, colored by cluster identity. **D.** Selection of the top 2,000 highly variable genes across cells based on initial clustering. **E.** Violin plots showing the distribution of feature counts, total counts, and mitochondrial gene percentages. **F.** UMAP showing refined clustering of facial cell populations from the scRNA-seq dataset, excluding endothelium, blood, immune, and forebrain-derived cells. **G.** Scatter plot of number of features versus number of counts for the final clustering of facial populations, colored by cluster. **H.** Screening of the top 2,000

highly variable genes across cells based on the facial subset of the chicken scRNA-seq data. *Abbreviations:* mes, mesenchyme; ecto, ectoderm; fb, forebrain; endo, endothelium; immune, immune cells; LNP, lateral nasal prominence; MxP-NP, mesenchyme spanning maxillary prominence to nasal pits; FNM, frontonasal mass mesenchyme; Dorsal mes, dorsal mesenchyme; olf epithelium, olfactory epithelium. Figure created in BioRender. Kyomen, S. (2026) https://BioRender.com/g01q360

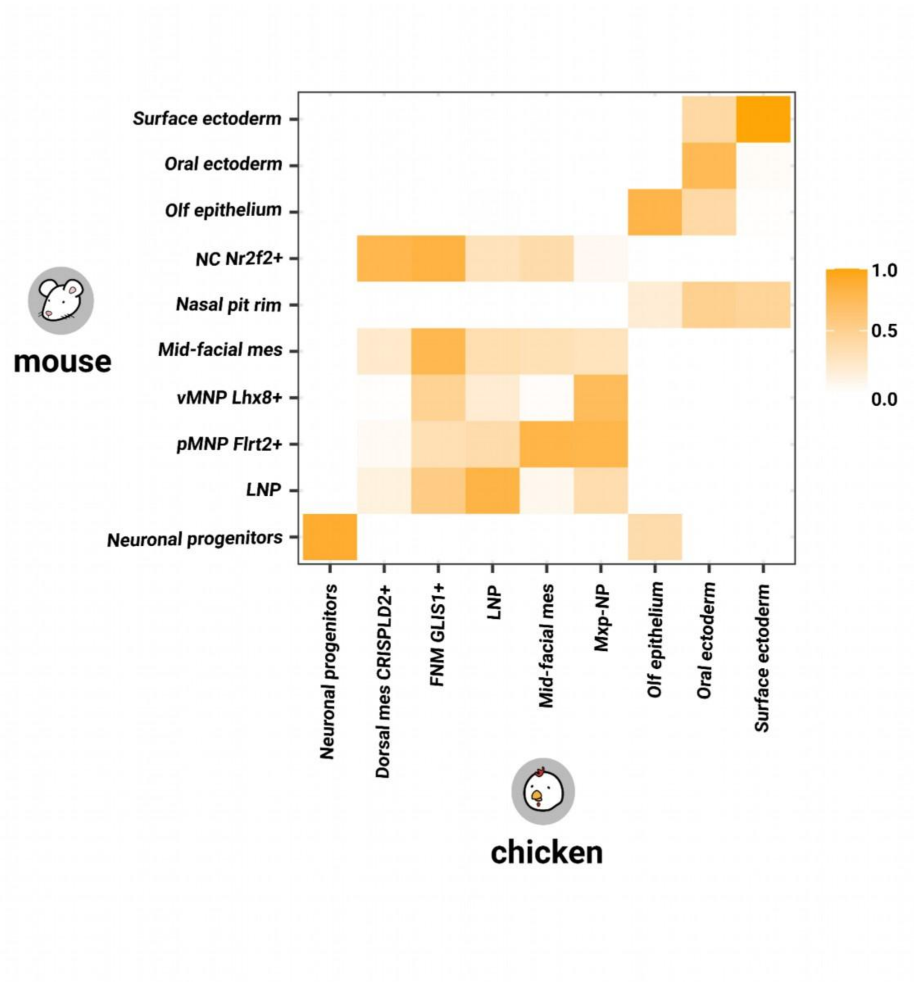

Figure S4. Heatmap of SAMap alignment scores comparing mouse (Y-axis) and chicken (X-axis) scRNA-seq clusters. Higher scores (dark orange) indicate stronger cross-species transcriptomic correspondence. Ectodermal clusters show more direct alignments than mesenchymal clusters, which display broader and more diffuse mapping scores. Figure created in BioRender. Kyomen, S. (2026) https://BioRender.com/g01q360

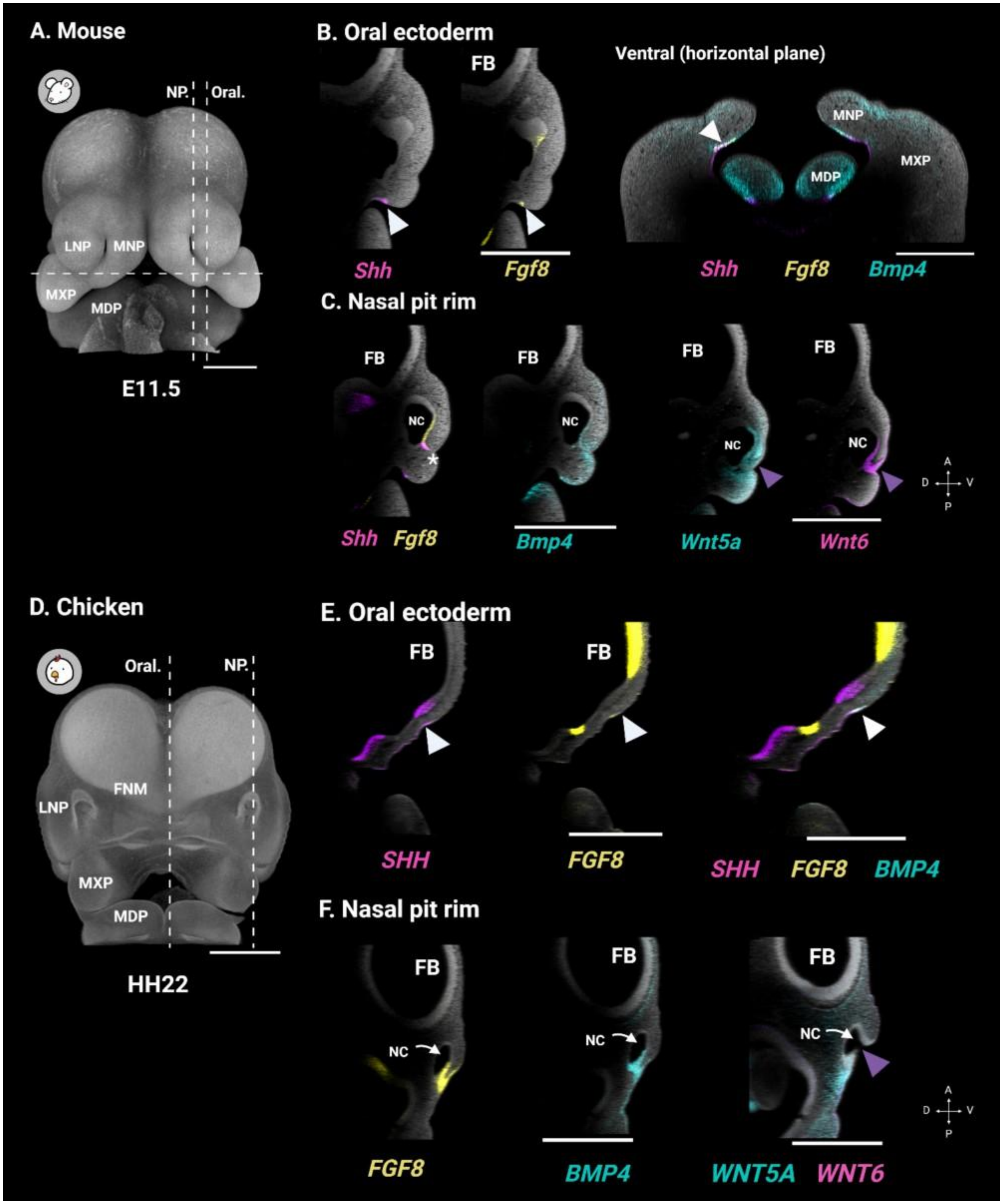

Figure S5. *Shh, Fgf8,* and *Bmp4* expression in mouse and chicken facial prominences. A, D. Representation of facial prominences indicating the position of cross-section in mouse (A) and

chicken (D) embryos**. B, E.** Cross-sections showing *Shh*, *Fgf8*, and *Bmp4* expression domains in the oral ectoderm. Note the small domain where *Shh, Fgf8,* and *Bmp4* co-localize, overlapping with the FEZ in both mouse (B, white arrowhead) and chicken (E, white arrowhead). **C, F.** Cross-sections from *in situ* HCR showing morphogen expression in the nasal pit rim. Note the mouse-specific *Shh* domain in the ventral nasal pit rim (white asterisk). *Wnt5a* and *Wnt6* expression in the nasal pit rim are also absent in chicken (purple arrowhead). Scale bars: 500 µm. *Abbreviations:* FNP, frontonasal process; MXP, maxillary prominence; MDP, mandibular prominence; LNP, lateral nasal prominence; MNP, medial nasal prominence; FNM, frontonasal mass; NP, nasal pit; FB, forebrain; NC, nasal cavity; D, dorsal; V, ventral; A, anterior; P, posterior. Figure created in BioRender. Kyomen, S. (2026) https://BioRender.com/g01q360

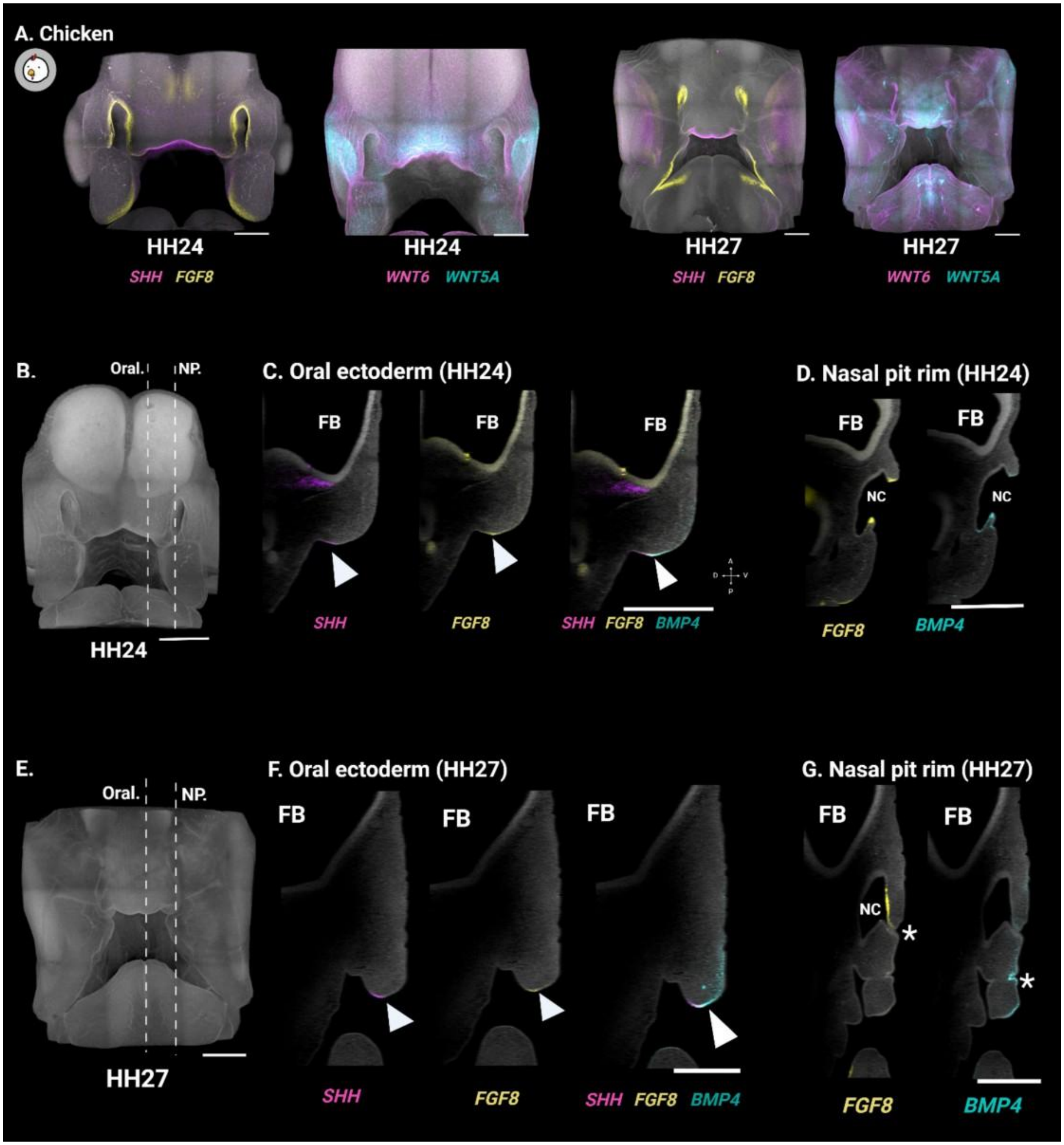

Figure S6. Expression of selected ligands during late-stage face development in chicken. A. *in situ* HCR showing morphogen expression in HH24 (left) and HH27 (right) chicken embryos. **B, E.** Representation of facial prominences indicating position of cross-sections in HH24 (B) and HH27 (E) chicken embryos. **C, F.** Cross-section of *in situ* HCR showing expression domains of

SHH, *FGF8* and *BMP4* in the oral ectoderm of HH24 (C) and HH27 (F) chicken embryos. Following the fusion of the frontonasal mass with the maxillary process at HH27, *SHH* expression persists in the oral ectoderm but becomes highly localized to the tip of the prospective beak. The region where *SHH, FGF8,* and *BMP4* co-localize remains detectable at the assessed stages (white arrowhead). **D, G.** Cross-section of *in situ* HCR showing expression domains of *FGF8* and *BMP4* in the nasal pit rim of HH24 (D) and HH27 (G) chicken embryos. In later stages, the *FGF8* domain in the nasal pit rim becomes progressively restricted to the olfactory epithelium, while *BMP4* is confined to the surface ectoderm (white asterisks). Notably, *SHH* and *WNT5A* remain absent from the nasal pit rim at these stages, differing from what is observed in mice. Scale bars represent 500 µm. *Abbreviations:* FNP, frontonasal process; MXP, maxillary prominence; MDP, mandibular prominence; LNP, lateral nasal prominence; MNP, medial nasal prominence; FNM, frontonasal mass; NP, nasal pit; FB, forebrain; NC, nasal cavity. Figure created in BioRender. Kyomen, S. (2026) https://BioRender.com/g01q360

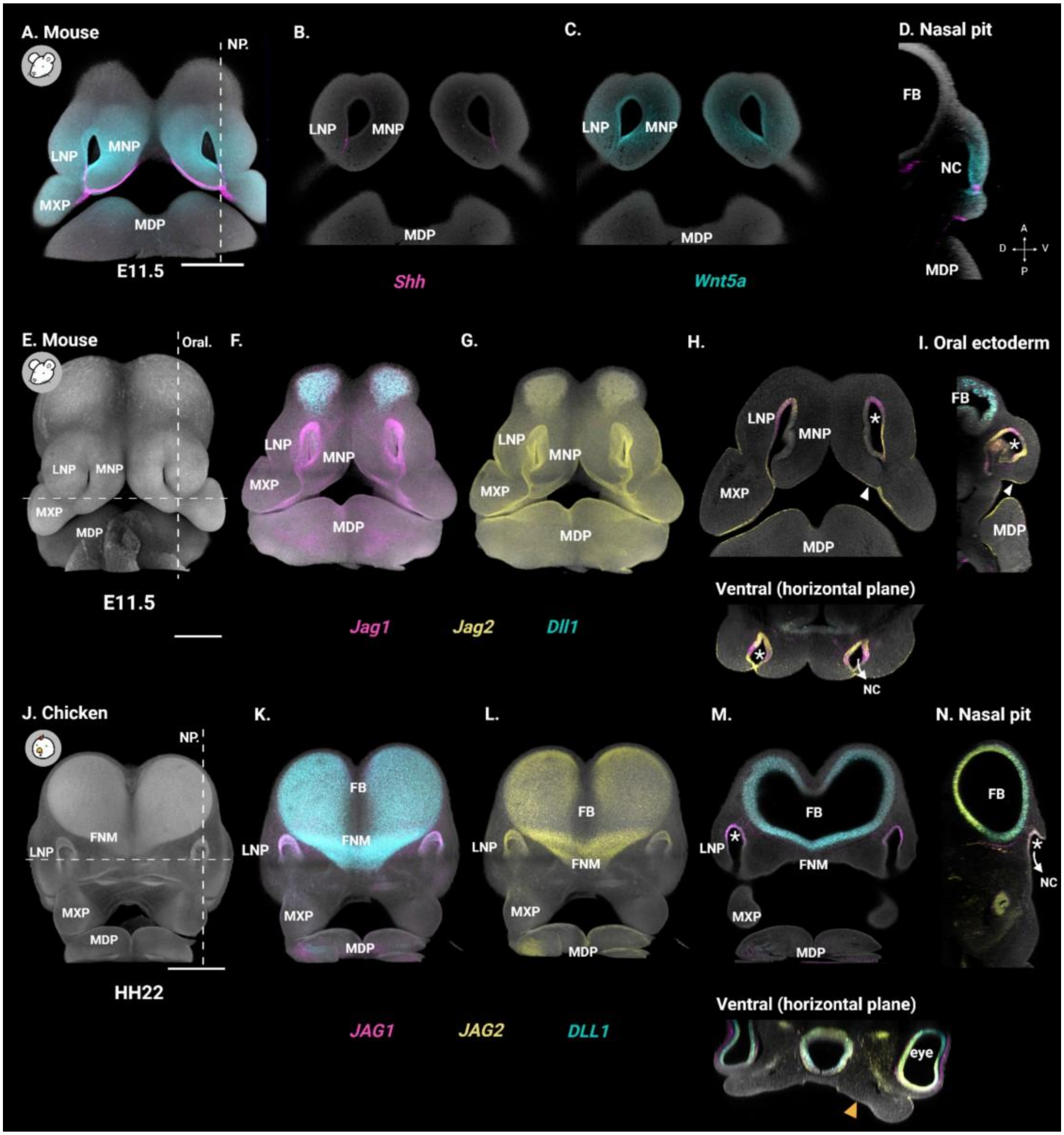

Figure S7. Expression of *Shh* and *Wnt5a* in the nasal pit rim of mouse embryos. A. *in situ* HCR showing the expression of *Shh* and *Wnt5a* in the facial prominences of mouse E11.5 embryo. **B, C.** Cross-sections of *in situ* HCR showing the expression of *Shh* in the caudal part of the nasal pit rim (B) and *Wnt5a* in the nasal pit rim and LNP/MNP mesenchyme (C). **D.** Lateral view of *Shh* and *Wnt5a* domains in the nasal pit rim. **E**. Representation of facial prominences indicating

position of cross-sections in mouse embryo at E11.5. **F, G.** Expression of *Jag1*, *Jag2*, and *Dll1* in facial prominences at E11.5 in mouse embryo. *Dll1* is strongly expressed in the forebrain and in discrete neuron-rich regions of the olfactory epithelium. **H, I.** *Jag1* and *Jag2* are prominently expressed in the olfactory epithelium (white asterisks) and are also detected in the oral ectoderm, nasal pit rim, and surface ectoderm, particularly over the mandibular prominence (white arrowheads). **J.** Representation of facial prominences indicating the position of cross-sections in chicken embryo at HH22. **K, L.** *In situ* HCR showing expression of *JAG1*, *JAG2*, and *DLL1* in chicken facial prominences at HH22. As in mouse, *DLL1* is highly expressed in the forebrain. *JAG1* expression is restricted to the olfactory epithelium, whereas *JAG2* is additionally detected in the surface ectoderm covering the mandibular prominence but not in the frontonasal mass. **M, N.** Cross-sections showing *JAG1* expression in discrete regions of the olfactory epithelium (white arrowheads) and the absence of *JAG1/2* expression in the oral ectoderm of chicken embryos (orange arrowheads). Scale bars: 500 µm. *Abbreviations:* MXP, maxillary prominence; MDP, mandibular prominence; LNP, lateral nasal prominence; MNP, medial nasal prominence; FB, forebrain; FNM, frontonasal mass; D, dorsal; V, ventral; A, anterior; P, posterior. Figure created in BioRender. Kyomen, S. (2026) https://BioRender.com/g01q360

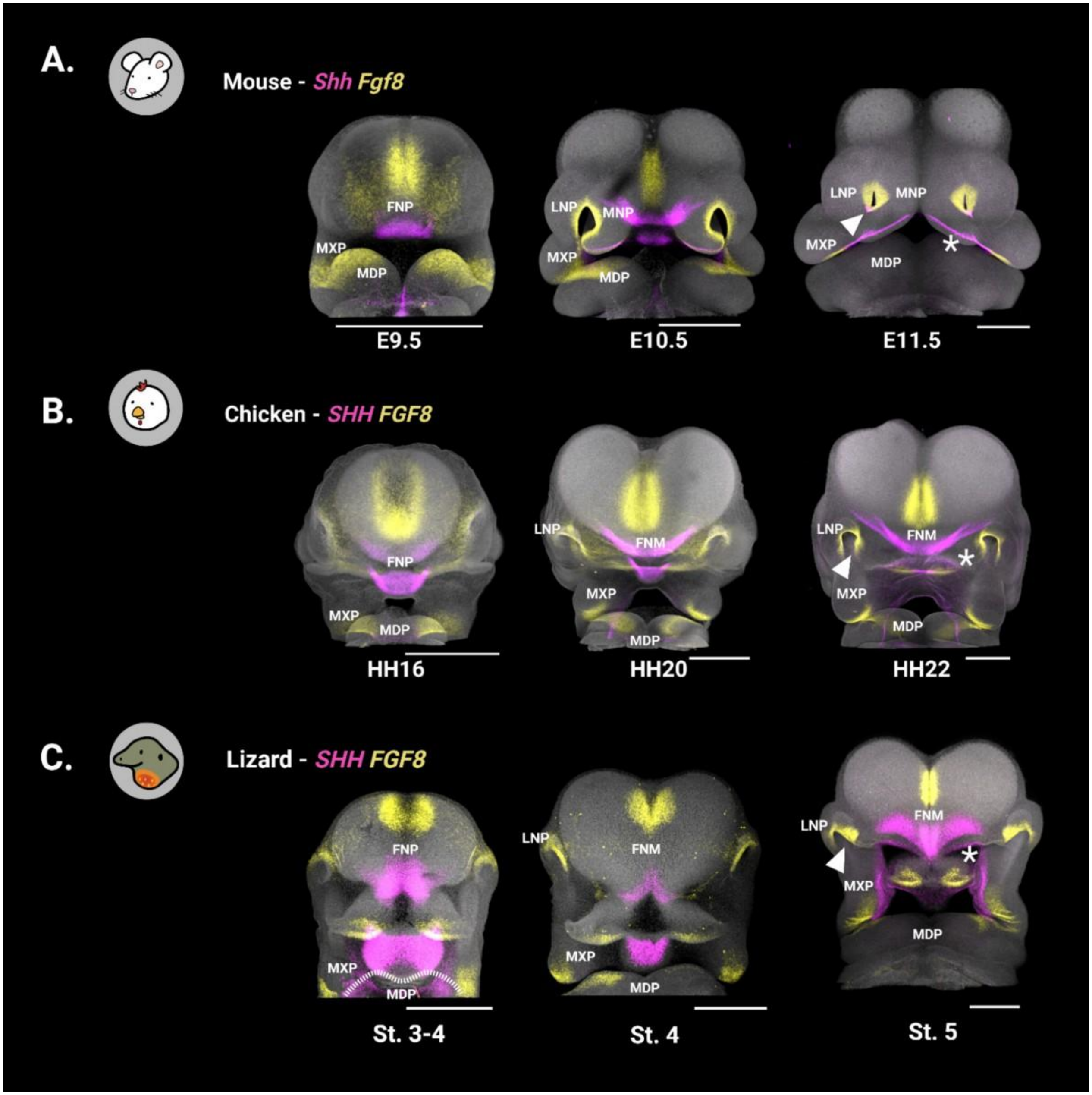

Figure S8. Expression of *Shh* and *Fgf8* in the facial prominences of mouse, chicken, and lizard embryos. A, B, C. *in situ* HCR showing the expression of *Shh* and *Fgf8* in the facial prominences of mouse (A), chicken (B) and lizard (C) embryos during early facial development. The small SHH-positive domain at the caudal rim of the nasal pit (white arrowhead) is detected only in mouse, whereas broad *Shh* expression in the oral ectoderm (white asterisk) is conserved across all three species. Scale bars: 500 µm. *Abbreviations:* MXP, maxillary prominence; MDP, mandibular prominence; LNP, lateral nasal prominence; MNP, medial nasal prominence; FB, forebrain; D, dorsal; V, ventral; A, anterior; P, posterior. Panels A and B are reproduced from Figure 1A to facilitate direct comparison with *SHH* and *FGF8* expression in lizard embryos. Figure created in BioRender. Kyomen, S. (2026) https://BioRender.com/g01q360

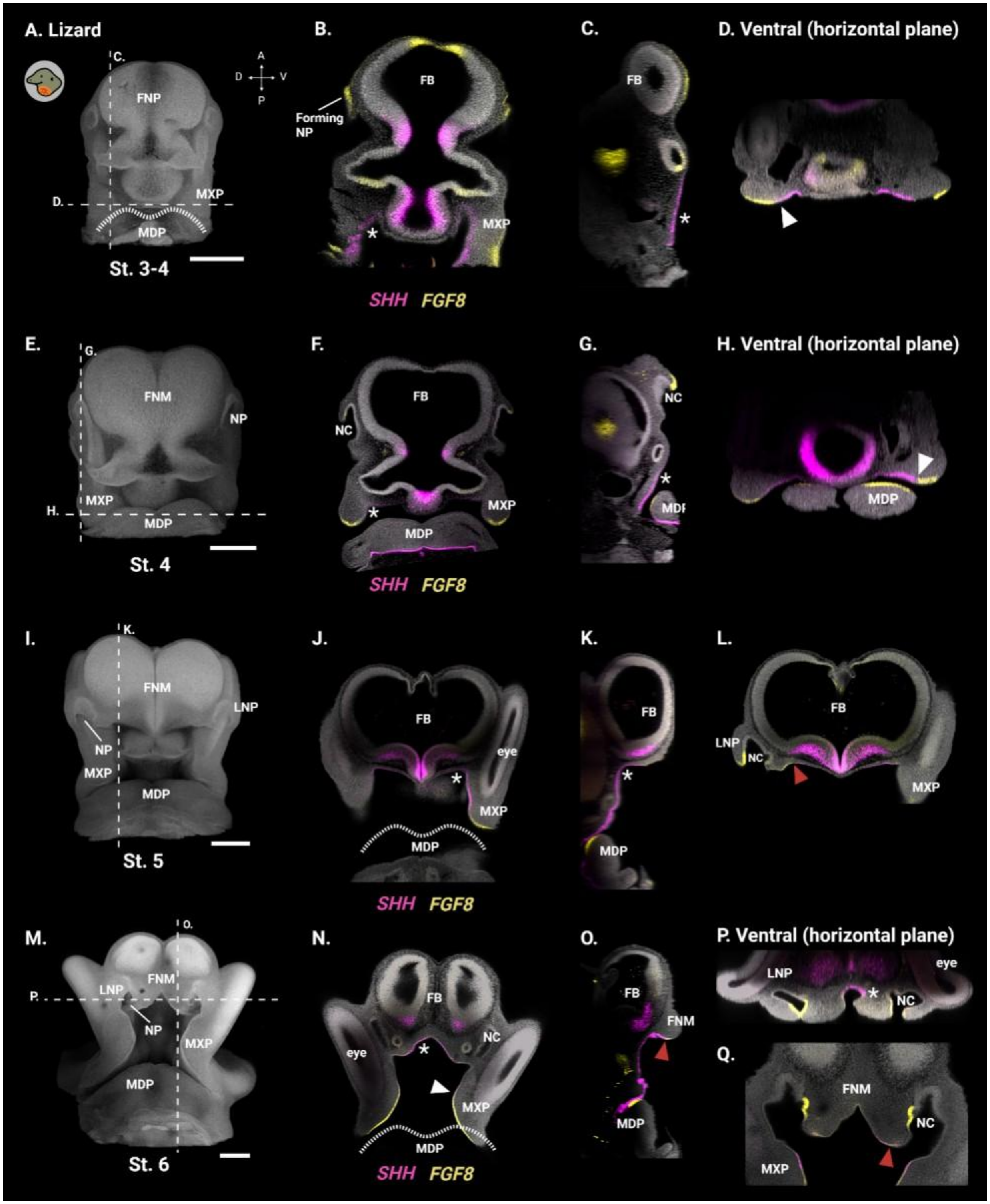

Figure S9. Expression of *SHH* and *FGF8* in the facial prominence of *Anolis sagrei* embryos. **A, E, I, M.** Representation of facial prominences indicating position of cross-sections in embryonic stages St. 3-6. At stages 3-4, *SHH* is expressed in the ectoderm lining the posterior oral cavity (white asterisks in B, C, F, G) and forms a sharp border with *FGF8* in the maxillary

prominence (white arrowheads in D, H). At later stages, *SHH* expression expands to line the full oral ectoderm (white asterisks in J, K, N, P), while the SHH-FGF8 interface in the maxillary prominence remains evident through stage 6 (N, white arrowhead). *SHH* and *FGF8* also show partial co-localization in discrete regions of the anterior oral ectoderm (L, O, Q, red arrowheads). Scale bars: 500 µm. *Abbreviations:* MXP, maxillary prominence; MDP, mandibular prominence; LNP, lateral nasal prominence; MNP, medial nasal prominence; FB, forebrain; D, dorsal; V, ventral; A, anterior; P, posterior. Figure created in BioRender. Kyomen, S. (2026) https://BioRender.com/g01q360

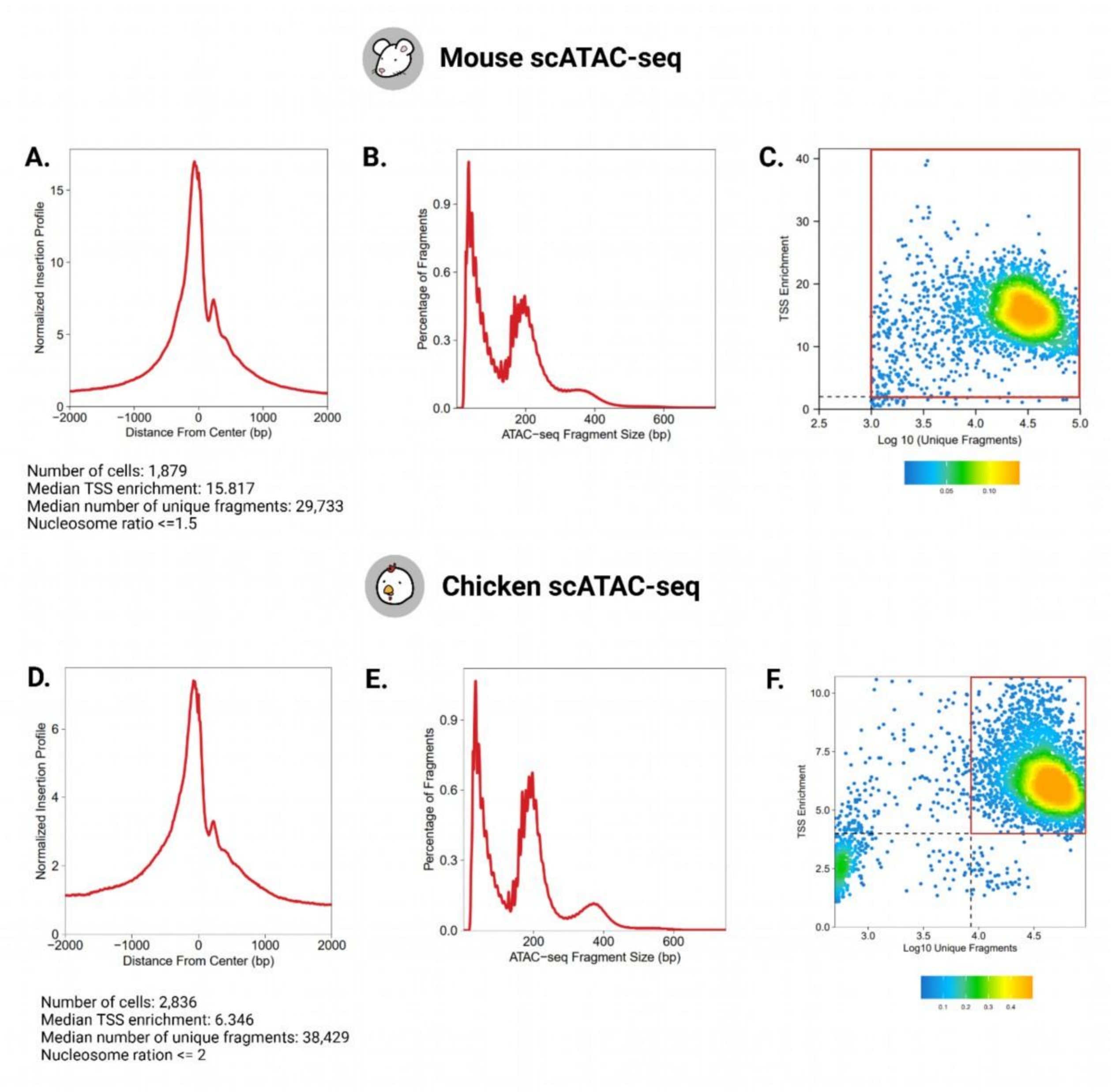

Figure S10. Quality control of scATAC-seq data. A, D. TSS enrichment profiles of mouse (A) and chicken (D) scATAC-seq data. Additional information on quality control of each sample is displayed below. **B**, **E.** Fragment size distribution of mouse (B) and chicken (E) scATAC-seq data. **C, F.** TSS enrichment score plotted against unique nuclear fragments per cell. Color scale represents point density in arbitrary units. Dashed lines mark applied thresholds and red box highlights points that meet filtering criteria. Figure created in BioRender. Kyomen, S. (2026) https://BioRender.com/g01q360

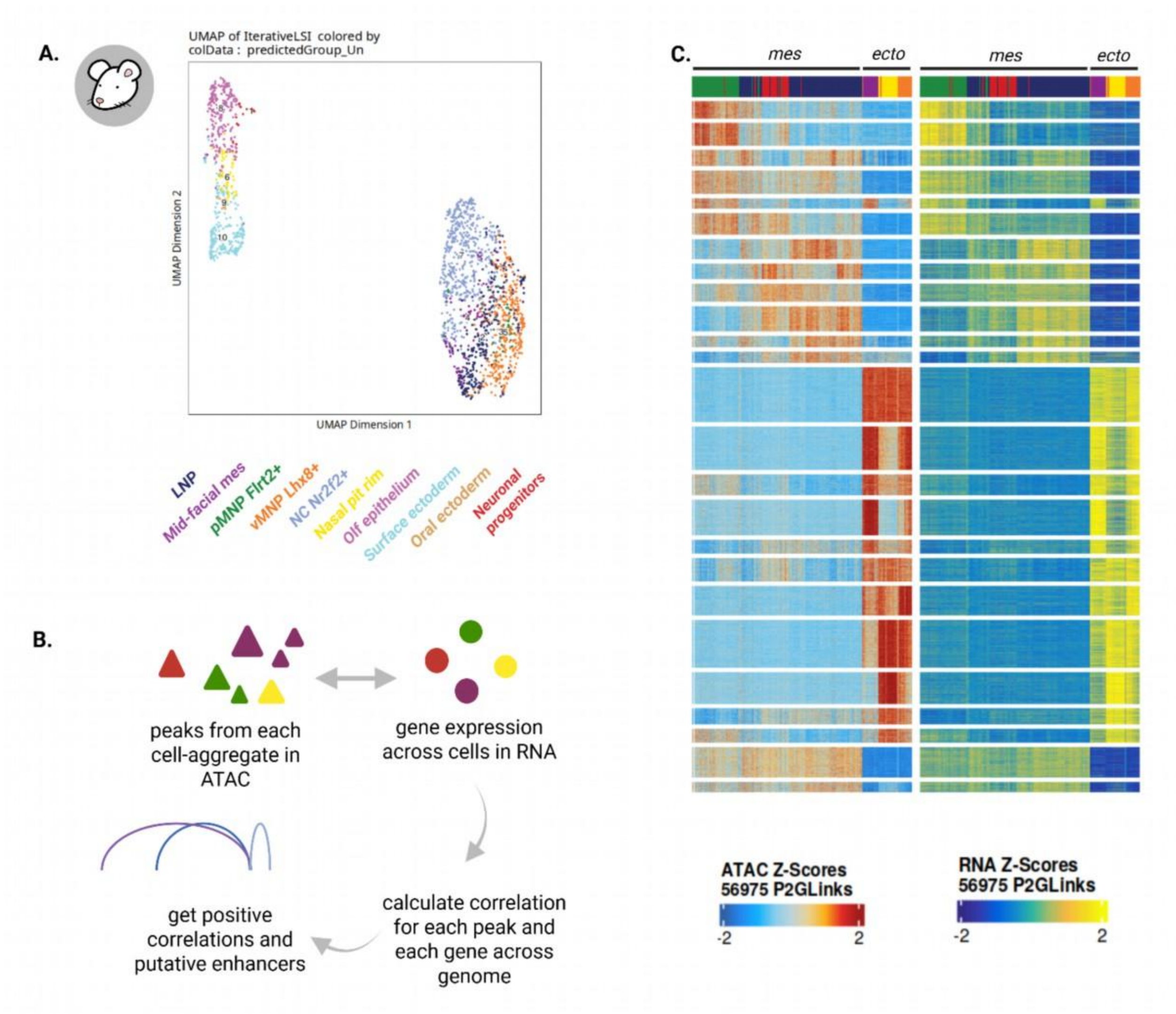

Figure S11. Visualization of ATAC-RNA integration in mouse. A. UMAP showing predicted groups obtained from unconstrained ATAC-RNA integration in mouse. **B.** Schematic representation of peak-to-gene linkage analysis by ArchR. **C.** Heatmap of peak-to-gene links identified across the mouse scATAC-seq dataset. *Abbreviations:* LNP, lateral nasal prominence; mid-facial mes, mid-facial mesenchyme; vMNP, ventral medial nasal prominence; pMNP, posterior medial nasal prominence; NC, nasal cavity mesenchyme; olf epithelium, olfactory epithelium; mes, mesenchyme; ecto, ectoderm. Figure created in BioRender. Kyomen, S. (2026) https://BioRender.com/g01q360

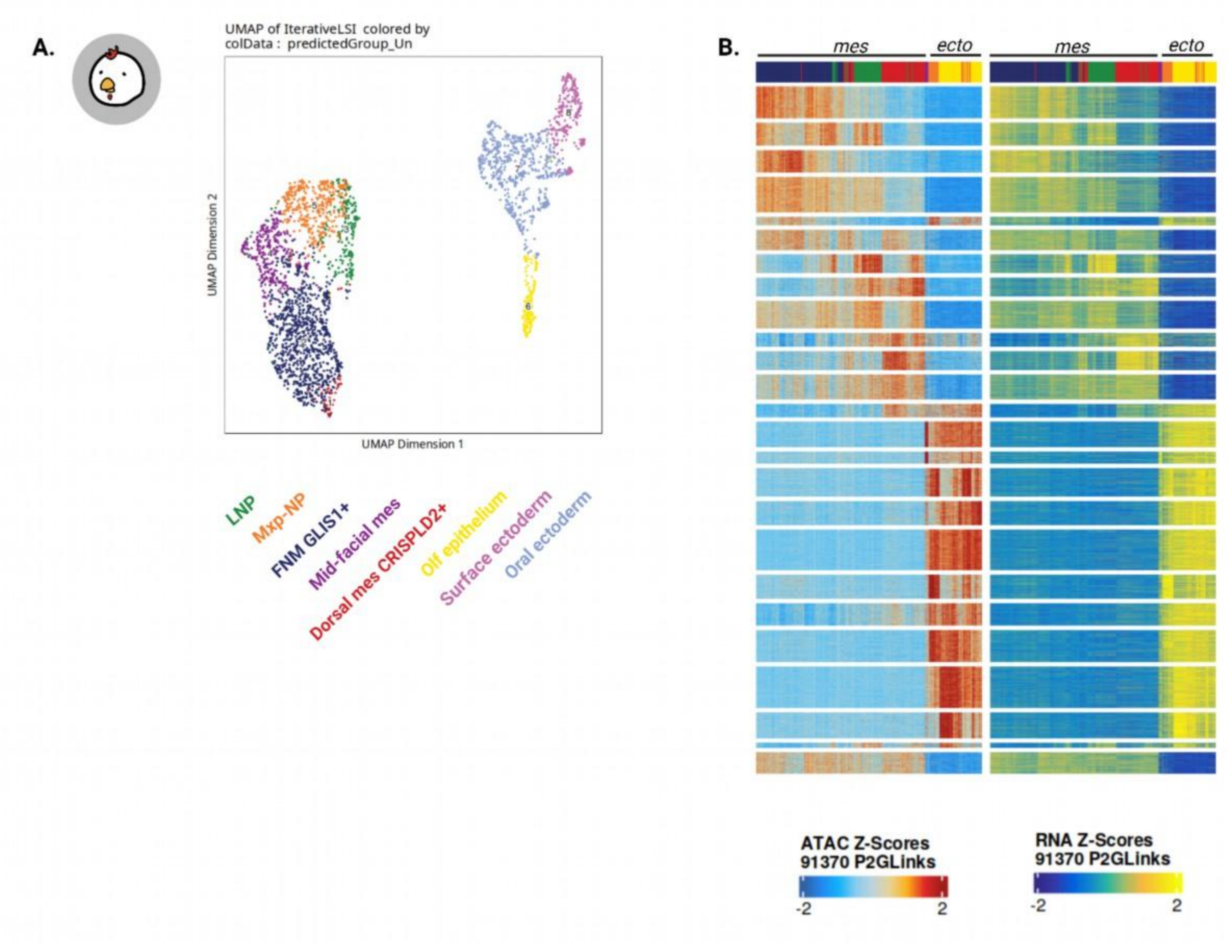

Figure S12. Visualization of ATAC-RNA integration in chicken. A. UMAP showing predicted groups obtained from unconstrained ATAC-RNA integration in chicken. **B.** Heatmap of peak-to-gene links identified across the chicken scATAC-seq dataset. *Abbreviations:* LNP, lateral nasal prominence; MxP-NP, mesenchyme spanning maxillary prominence to nasal pits; FNM, frontonasal mass mesenchyme; Dorsal mes, dorsal mesenchyme; olf epithelium, olfactory epithelium; mes, mesenchyme; ecto, ectoderm. Figure created in BioRender. Kyomen, S. (2026) https://BioRender.com/g01q360

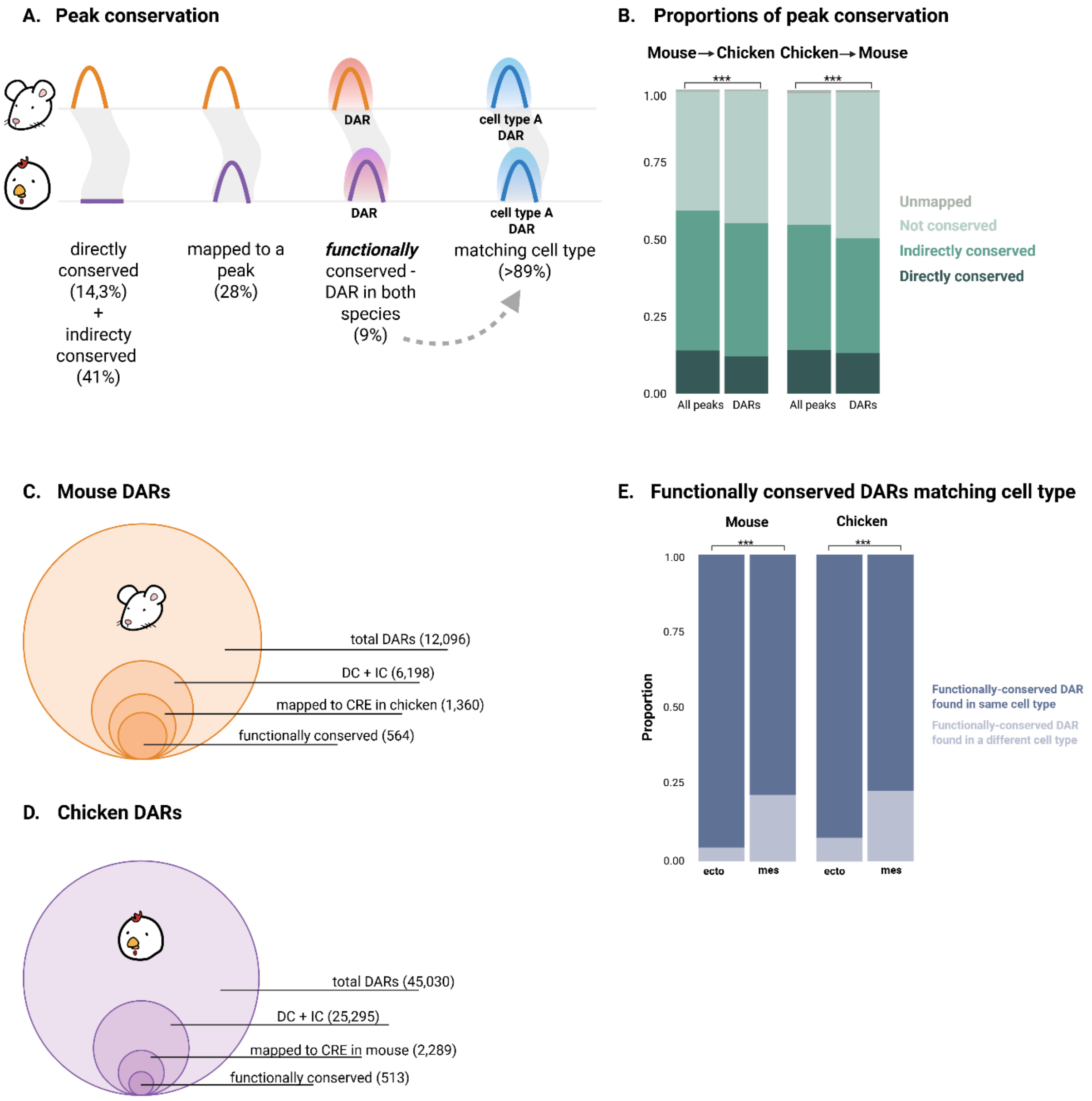

Figure S13. Conservation of DARs in mouse and chicken scATAC-seq data. A. Schematic illustration of the three levels of conservation assessed using IPP: positional conservation (directly and indirectly mapped), chromatin accessibility conservation (mapped to a peak), and functional conservation (shared DARs between species). **B.** Stacked bar plots showing the proportions of unmapped peaks, not conserved, indirectly and directly conserved for both directions: mouse to chicken (left) and chicken to mouse (right). **C.** Nested circle plot showing the proportions of conserved DARs from mouse data. Circle sizes are proportional to the number of elements at each conservation level. **D.** Nested circle plot showing the proportions of conserved DARs from chicken data. Circle sizes are proportional to the number of elements at each conservation level. **E.** Stacked bar plot showing the proportion of functionally-conserved DARs that retained the same cell type in the other species. Functionally-conserved ectodermal

DARs exhibited a markedly higher conservation rate compared to mesenchymal DARs (~4.4-fold higher, independent of species), consistent with cross-species similarity observed in SAMap analysis (Figure S4). P-values: $p < 0.001$ (***). Panel A shows the same schematic representation as in Figure 4J to facilitate comparison with the data presented in this figure. Figure created in BioRender. Kyomen, S. (2026) https://BioRender.com/g01q360

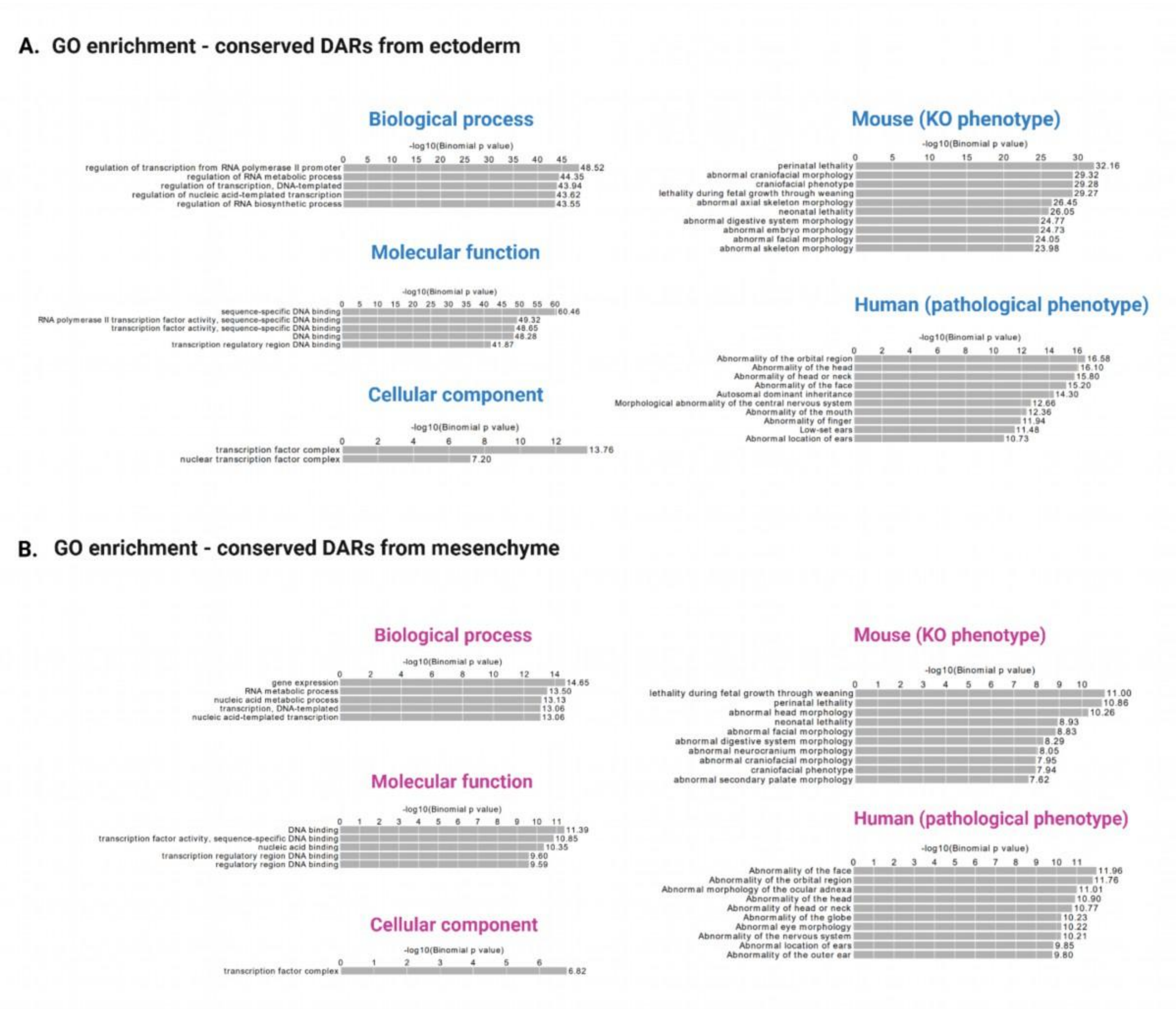

Figure S14. Gene Ontology of functionally-conserved DARs. A, B. Bar plots showing Gene Ontology enrichment for functionally-conserved DARs identified in ectodermal (A; 433 regions) and mesenchymal (B; 122 regions) populations. Enriched terms are grouped by biological process, molecular function, and cellular component, as determined using GREAT following the default parameters (5 kb upstream and 1 kb downstream basal plus extension for proximal regulatory regions, up to 1,000 kb for distal regions). The top ten mouse and human phenotypes associated with developmental malformations linked to these regions are also shown. Functionally-conserved DARs are predominantly associated with genes involved in transcriptional regulation and craniofacial development, supporting their potential roles in facial patterning programs. Figure created in BioRender. Kyomen, S. (2026) https://BioRender.com/g01q360

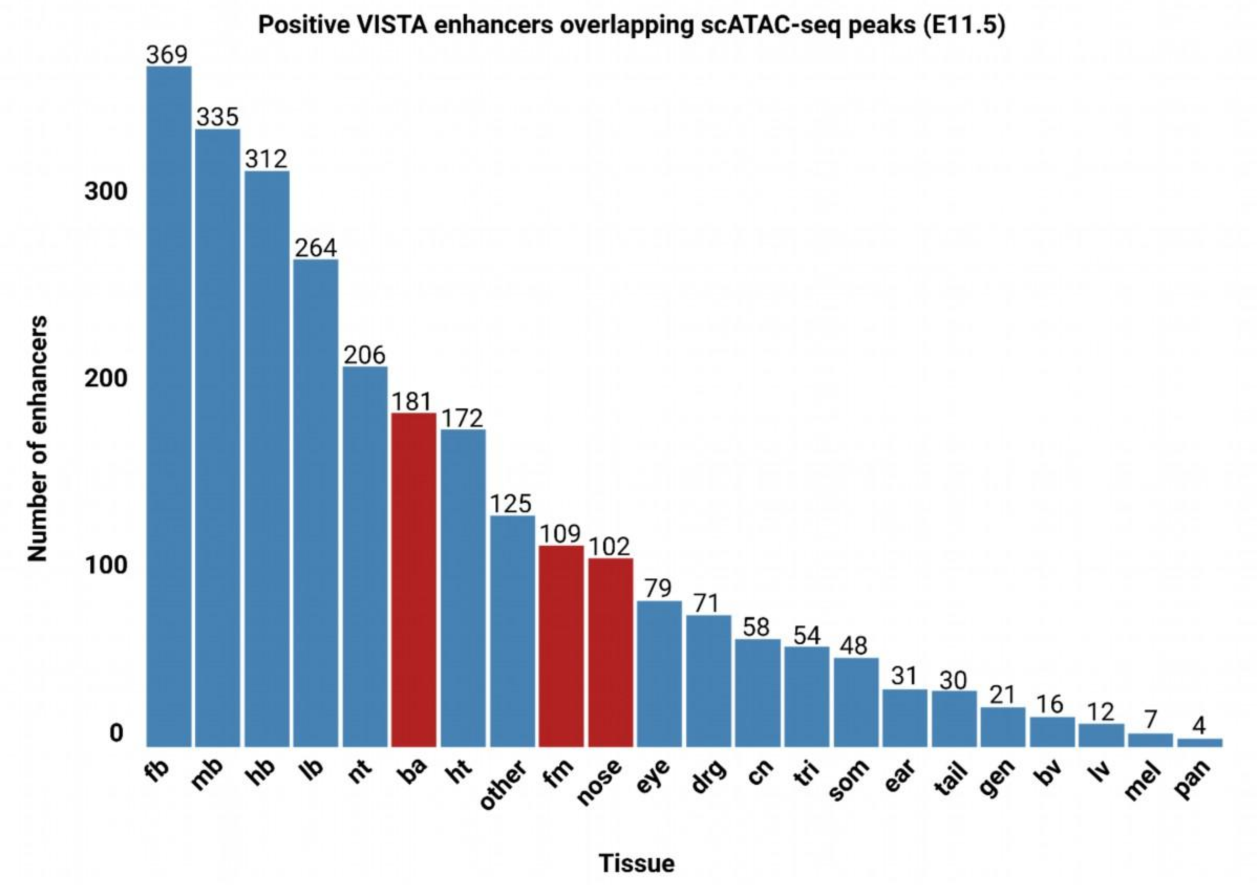

Figure S15. Number of *in vivo* validated enhancers per tissue at embryonic day 11.5 based on overlap between VISTA database and scATAC-seq peaks. Each bar represents a specific tissue, with the total number of *in vivo* validated enhancers indicated above. Only enhancers with confirmed *in vivo* activity ("positive" curation status) were included in the analysis. Because individual enhancers can exhibit activity in multiple tissues, the same enhancer may contribute to counts in more than one category. Enhancers active in craniofacial tissues (e.g., facial mesenchyme, nose, and branchial arches) are highlighted in red to emphasize tissue-specific regulatory activity. *Abbreviations*: fb, forebrain; mb, midbrain; hb, hindbrain; lb, limb; nt, neural tube; ba, branchial arch; ht, heart; fm, facial mesenchyme; drg, dorsal root ganglion; cn, cranial nerve; tri, trigeminal; som, somite; gen, genital tubercle; bv, blood vessels; lv, liver; mel, melanocytes; pan, pancreas. Figure created in BioRender. Kyomen, S. (2026) https://BioRender.com/g01q360

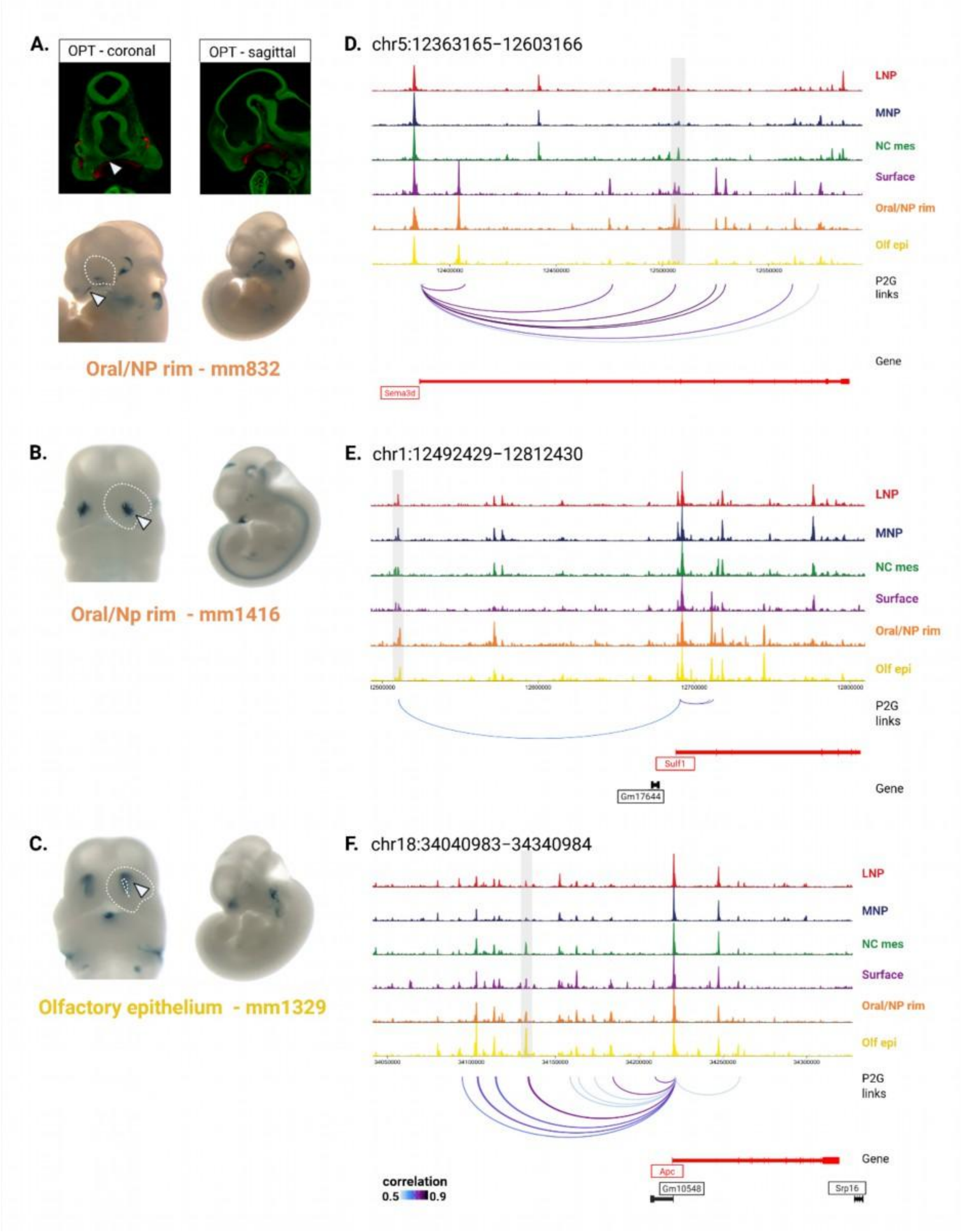

Figure S16. Selected *in vivo* validated enhancers associated with ectoderm-specific DARs. A, B, C. (Top) Optical Projection Tomography (OPT) scanning of transgenic E11.5 mouse embryos showing enhancer activity (red). (Bottom) Frontal and lateral views of transgenic mouse E11.5

embryos displaying enhancer activity in the ectoderm of frontonasal prominences. **D, E, F.** Genome track visualization of the *Sema3d*, *Sulf1*, and *Apc* locus. Each track represents the aggregated scATAC-seq signals from individual cell clusters in the mouse dataset, colored by cell cluster assignment. Gray boxes highlight specific cCREs located within DARs. Inferred peak-to-gene links between cCREs and the gene promoters are shown below the tracks, with darker colors indicating stronger correlations. *Abbreviations:* LNP, lateral nasal prominence; MNP, medial nasal prominence; NC mesenchyme, nasal cavity mesenchyme; Oral/NP rim, oral ectoderm and nasal pit rim; Olf epi, olfactory epithelium. Figure created in BioRender. Kyomen, S. (2026) https://BioRender.com/g01q360

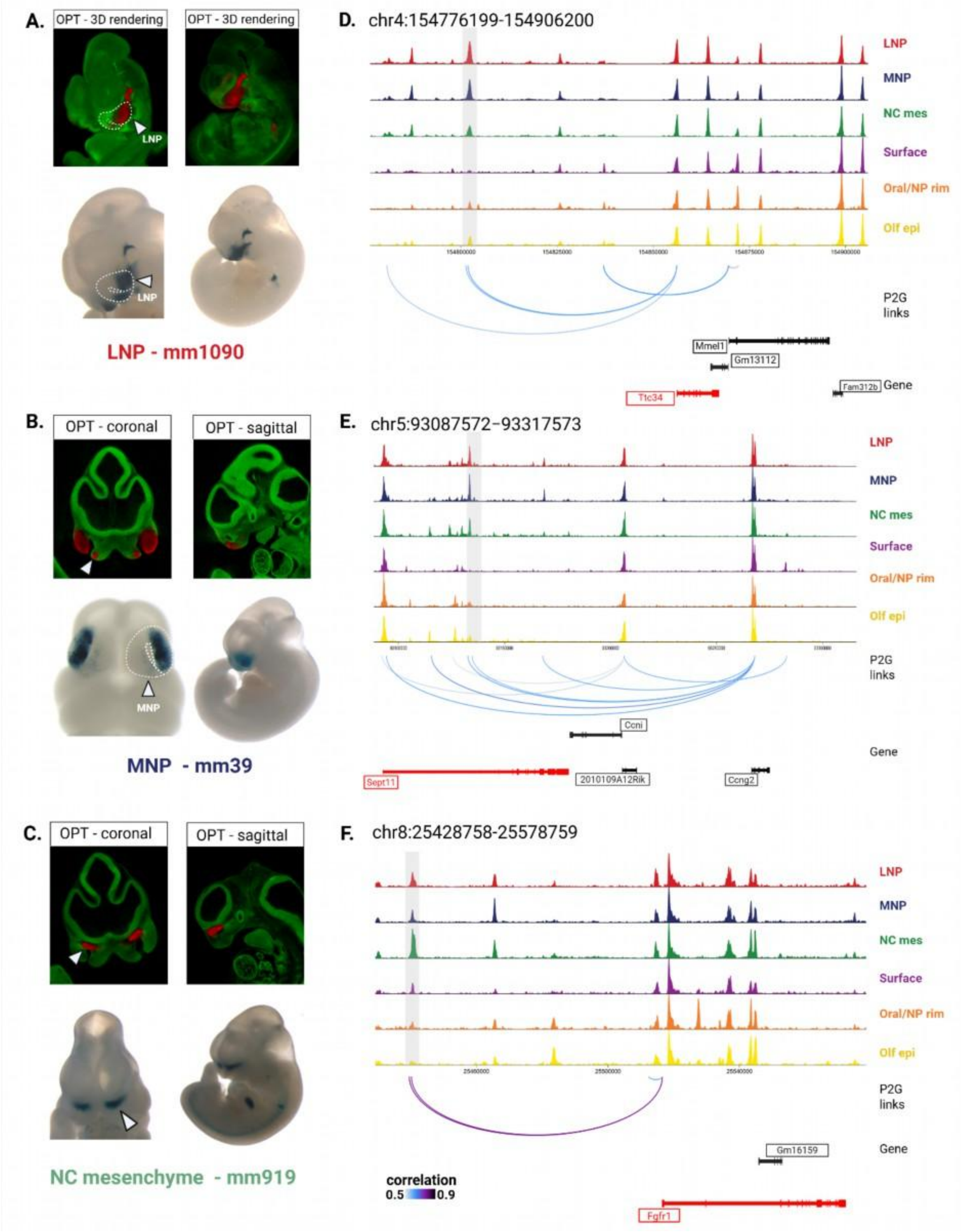

Figure S17. Selected *in vivo* validated enhancers associated with mesenchyme-specific DARs. A, B, C. (Top) Optical Projection Tomography (OPT) scanning of transgenic E11.5 mouse embryos showing enhancer activity (red). (Bottom) Frontal and lateral views of transgenic mouse

E11.5 embryos displaying enhancer activity in the mesenchyme of frontonasal prominences. **D, E, F.** Genome track visualization of the *Ttc34, Sept11*, and *Fgfr1* locus. Each track represents the aggregated scATAC-seq signals from individual cell clusters in the mouse dataset, colored by cell cluster assignment. Gray boxes highlight specific cCREs located within DARs. Inferred peak-to-gene links between cCREs and the gene promoters are shown below the tracks, with darker colors indicating stronger correlations. *Abbreviations:* LNP, lateral nasal prominence; MNP, medial nasal prominence; NC mesenchyme, nasal cavity mesenchyme; Oral/NP rim, oral ectoderm and nasal pit rim; Olf epi, olfactory epithelium. Figure created in BioRender. Kyomen, S. (2026) https://BioRender.com/g01q360

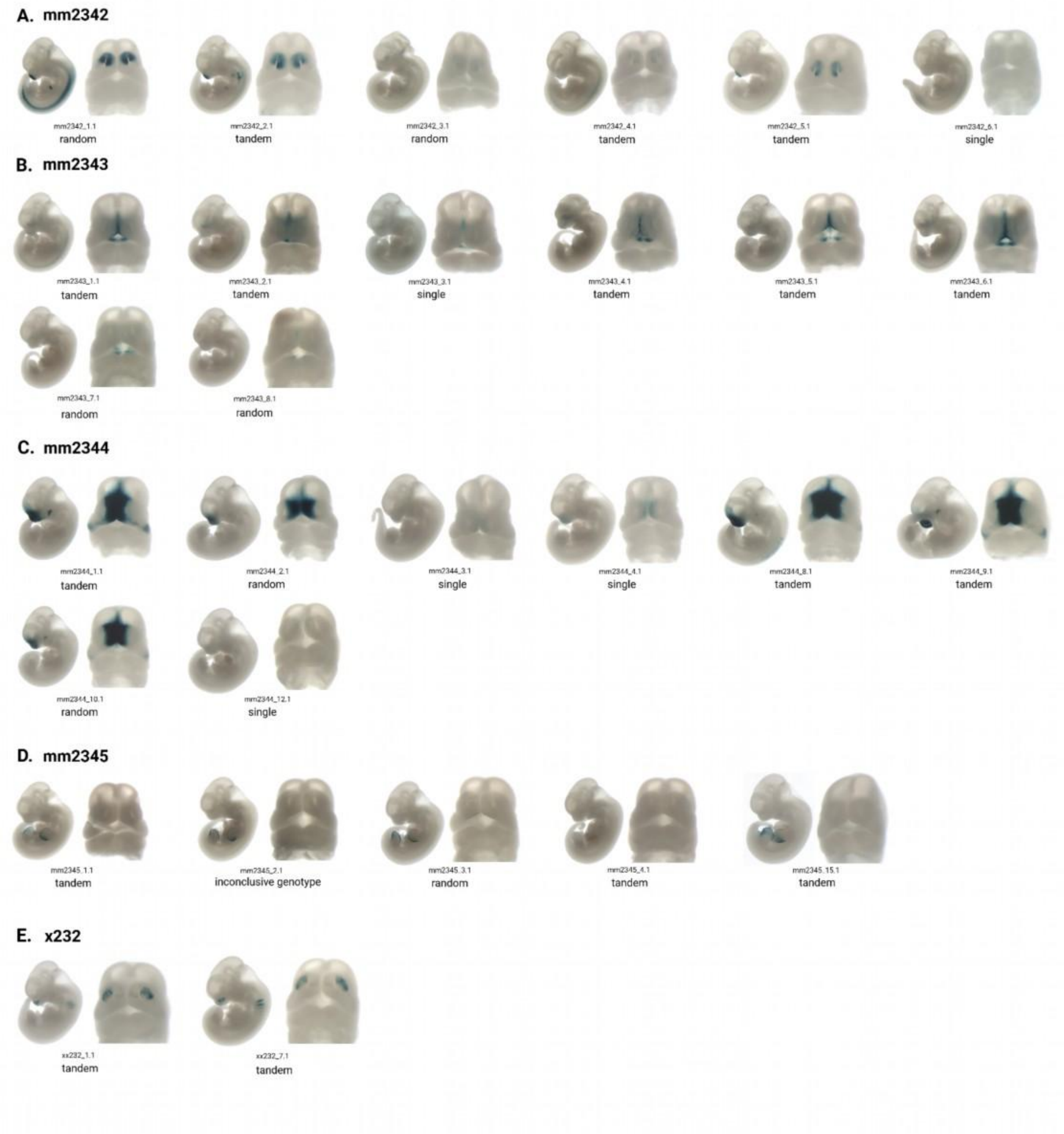

Figure S18. Replicate embryos of *in vivo* validated cCREs identified in this study. Frontal and lateral views of transgenic mouse E11.5 embryos showing reporter activity driven by each candidate enhancer. Genotyping PCR confirmed integration at the H11 locus and distinguished single-copy from tandem (concatemeric) insertions. Insertions at the H11 locus produced consistent reporter expression patterns across embryos, whereas tandem integrations typically resulted in stronger signal intensity. Embryos categorized as "random", which may include

integrations at the H11 locus and additional ectopic sites, displayed more variable reporter activity. Labels beneath each image correspond to individual embryo IDs. Figure created in BioRender. Kyomen, S. (2026) https://BioRender.com/g01q360

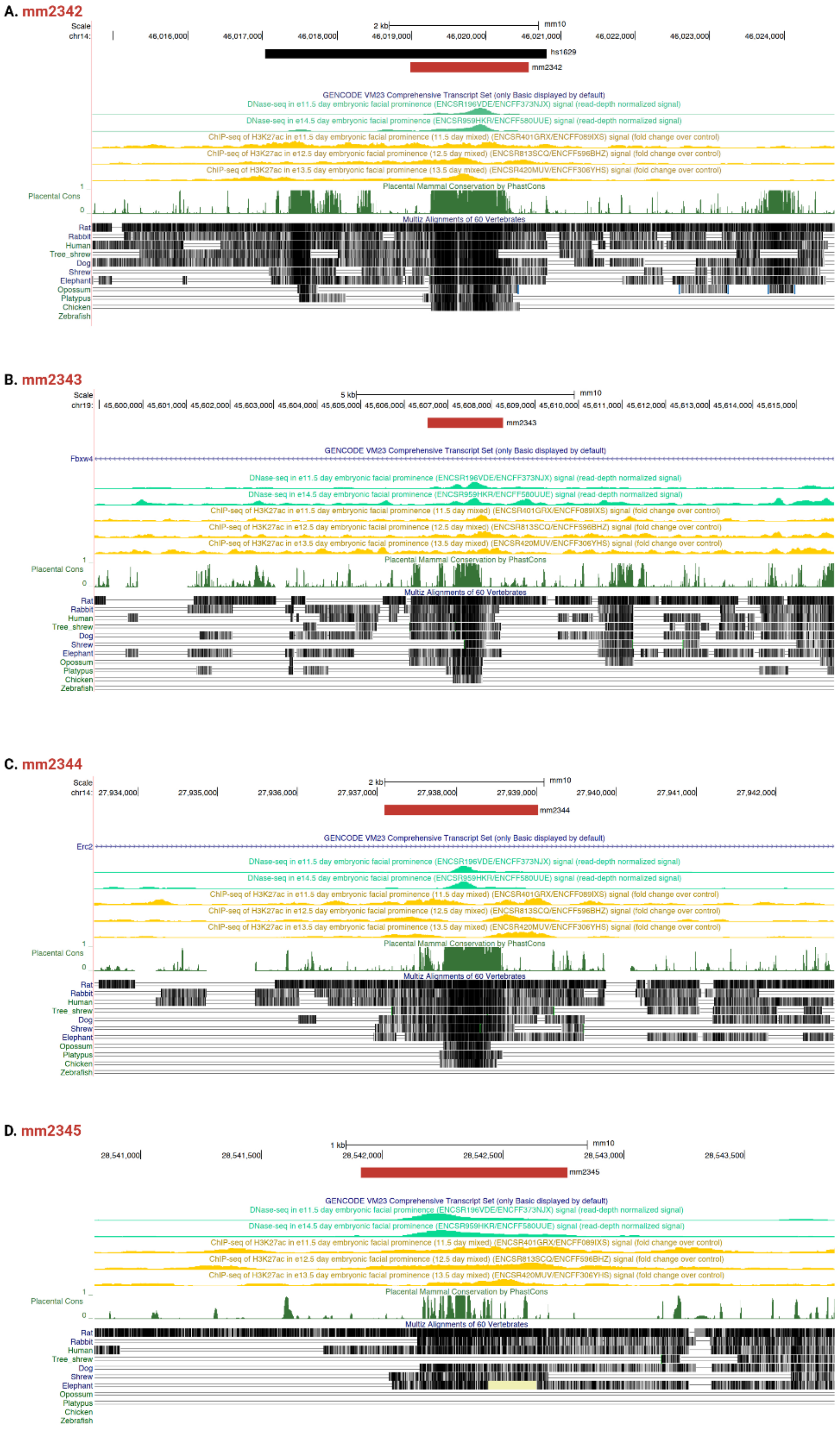

Figure S19. Genomic distribution and evolutionary conservation of *in vivo* validated cCREs. The genomic locations of mm2342 **(A)**, mm2343 **(B)**, mm2344 **(C)**, and mm2345 **(D)**, obtained from UCSC Genome Browser, alongside their overlap with DNase/ChIP-seq (H3K27ac) peaks from embryonic facial prominences. Evolutionary conservation across twelve vertebrate lineages, indicated by UCSC PhyloP scores, is shown below. Figure created in BioRender. Kyomen, S. (2026) https://BioRender.com/g01q360

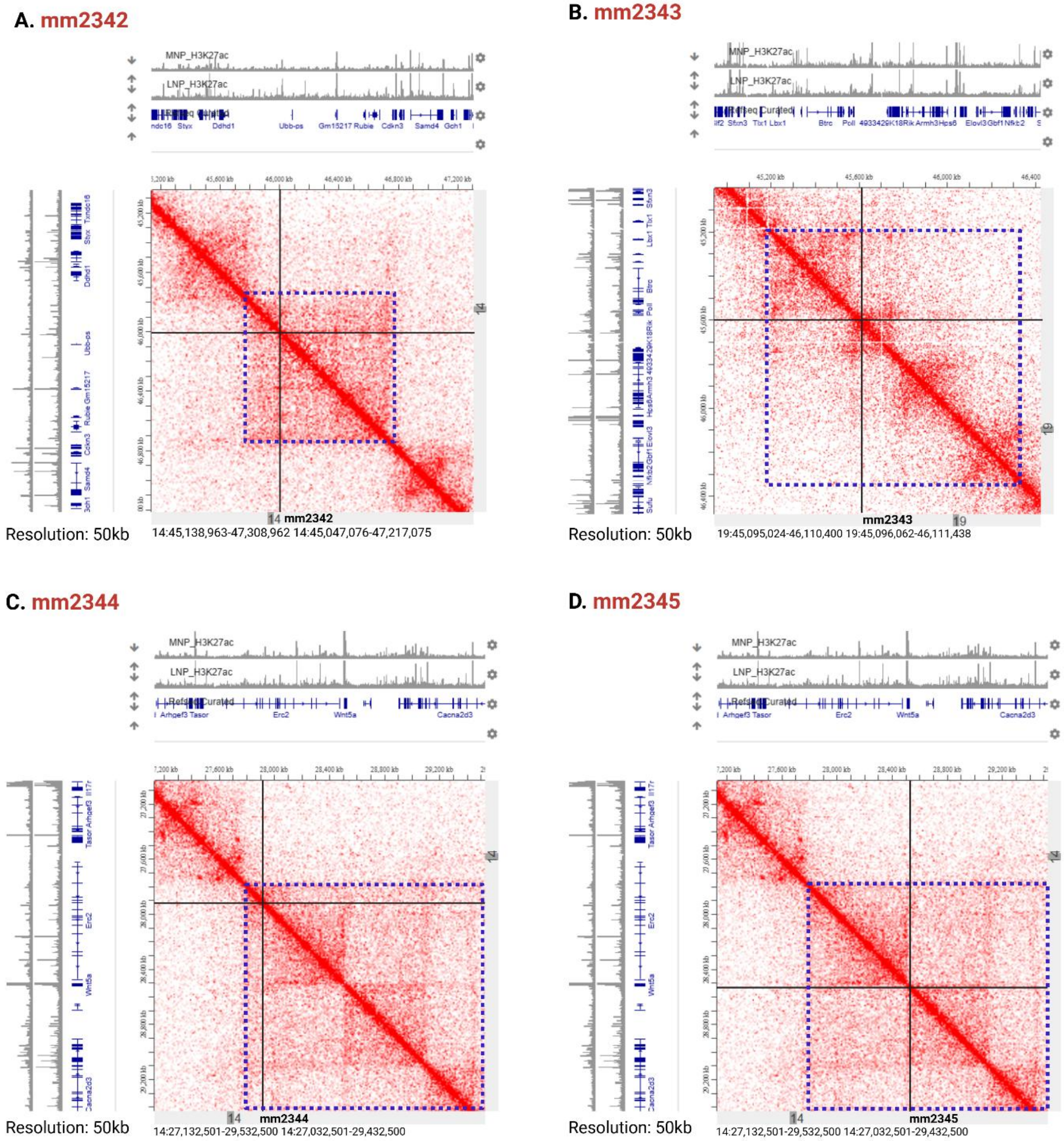

Figure S20. High-resolution Hi-C contact maps highlighting *in vivo* validated cCREs in mouse. Panels A-D show contact matrices centering four genomic loci: mm2342 **(A)**, mm2343 **(B)**, mm2344 **(C)**, and mm2345 **(D)**, at 50 kb resolution, each spanning ~2.5 Mb of intra-chromosomal interactions. Hi-C data from E11.5 mouse facial prominences were visualized using the Juicebox Web App with balanced normalization. ChIP-seq tracks (H3K27ac) from LNP and MNP of E11.5 embryos are shown alongside each matrix in gray. Black lines indicate the genomic

positions of *in vivo* validated enhancers, while blue dashed boxes mark topologically associating domains. Genomic coordinates corresponding to each window are shown beneath the maps. Figure created in BioRender. Kyomen, S. (2026) https://BioRender.com/g01q360

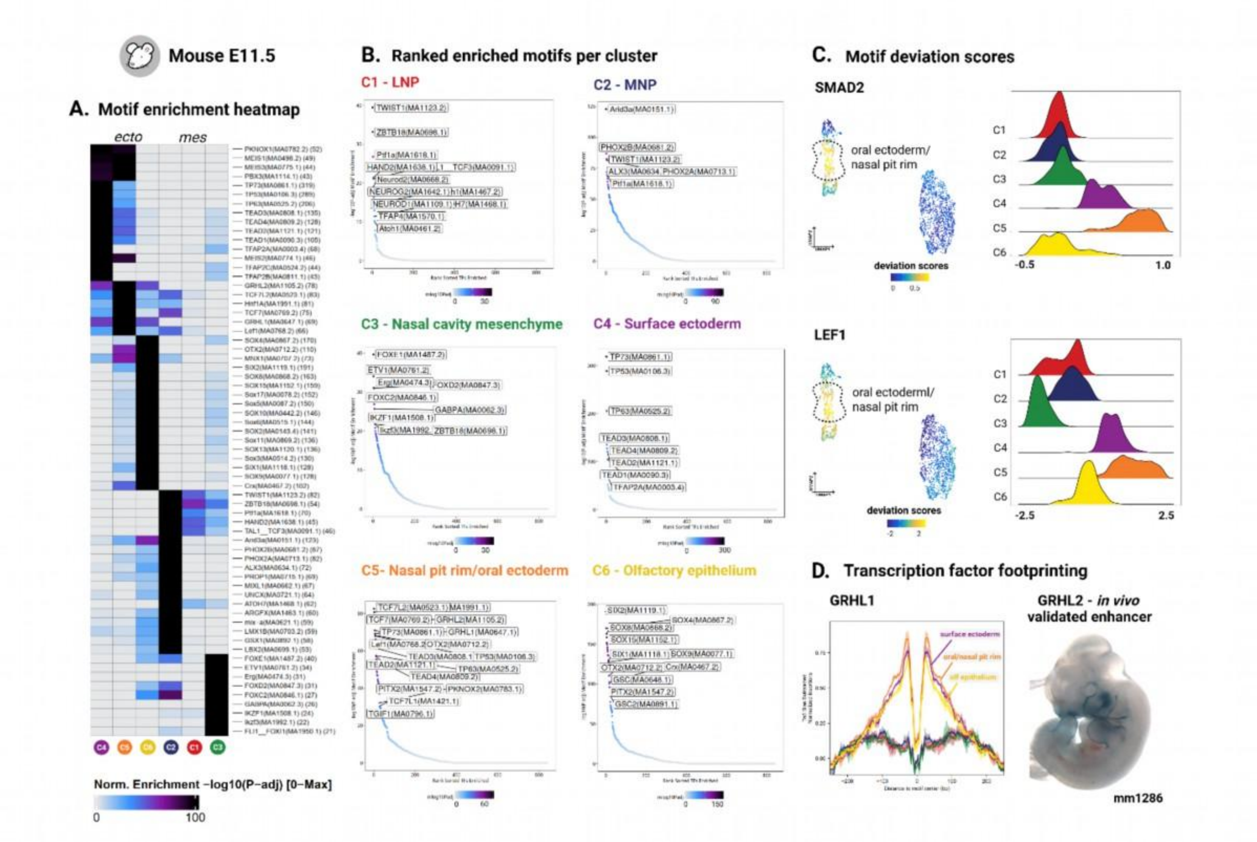

Figure S21. Identification of cell-type-specific TFBS motifs in mouse scATAC-seq data. A. Heatmap displaying enriched TF motifs specific to individual cell clusters (FDR ≤ 0.05, log2FC ≥ 1.5). Darker colors indicate higher motif enrichment. **B.** Ranked list of enriched TF motifs per cluster. Note the cell-population specificity of TF binding sites. **C.** (Left) Overlay of selected cluster-specific TFs on UMAP, based on chromVAR deviation scores. (Right) Distribution of chromVAR deviation scores across cell clusters, highlighting SMAD2 and LEF1 motifs. **D.** (Left) Tn5-bias-adjusted TF footprint showing ATAC-seq signal at predicted binding sites for GRHL1. (Right) Lateral view of transgenic mouse E11.5 embryo exhibiting positive enhancer activity associated with *Grhl2* in the surface ectoderm (mm1286). *Abbreviations:* LNP, lateral nasal prominence; MNP, medial nasal prominence; mes, mesenchyme; ecto, ectoderm. Figure created in BioRender. Kyomen, S. (2026) https://BioRender.com/g01q360

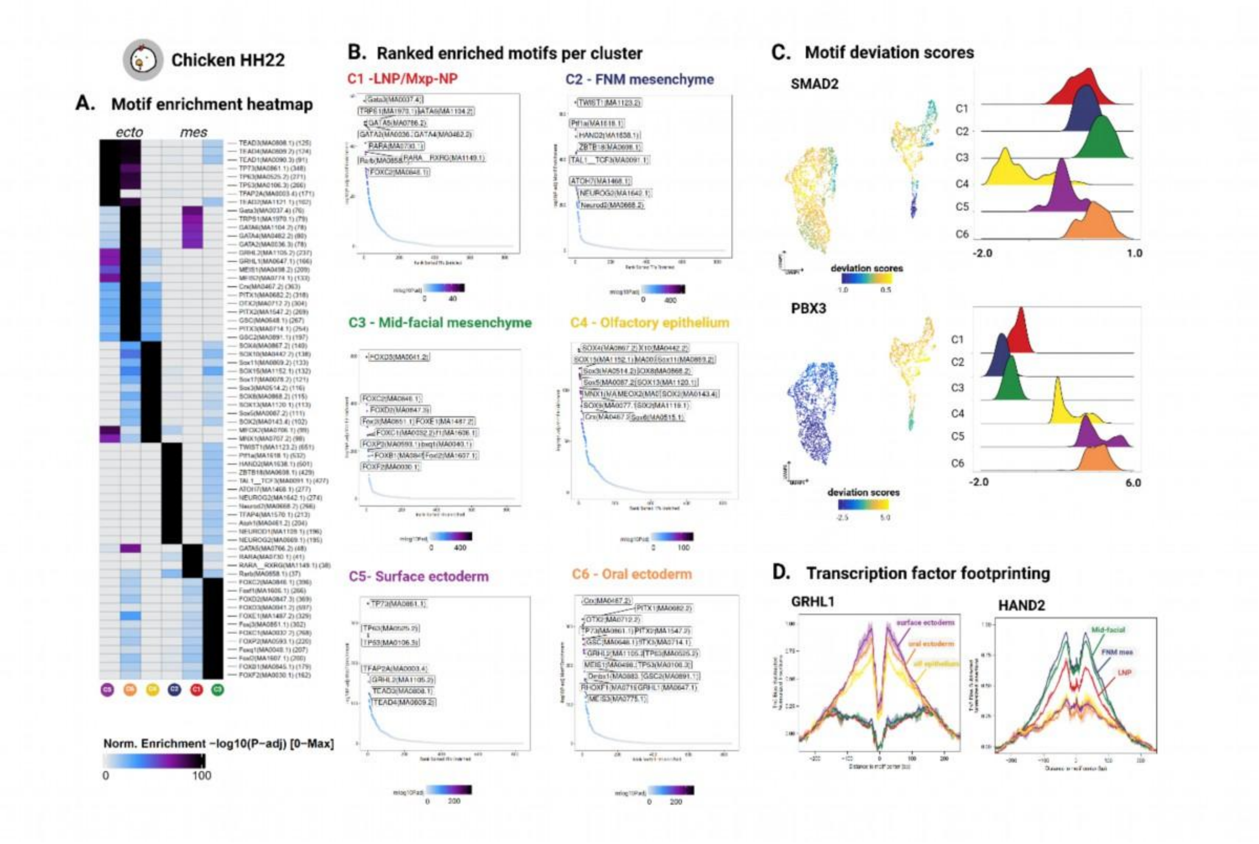

Figure S22. Identification of cell-type-specific TFBS motifs in chicken scATAC-seq data. A. Heatmap displaying enriched TF motifs specific to individual cell clusters (FDR ≤ 0.05, log2FC ≥ 1.5). Darker colors indicate higher motif enrichment. **B**. Ranked list of enriched TF motifs per cluster. Note the cell-population specificity of TF binding sites. **C.** (Left) Overlay of selected cluster-specific TFs on UMAP, based on chromVAR deviation scores. (Right) Distribution of chromVAR deviation scores across cell clusters. Note that SMAD2 motifs are found predominantly in the mesenchyme and the oral ectoderm clusters, while PBX3 motifs are specific to ectoderm**. D.** Tn5-bias-adjusted TF footprint showing ATAC-seq signal at predicted binding sites for GRHL1 and HAND2. Note the cell-cluster specificity of TF binding sites. *Abbreviations*: LNP, lateral nasal prominence; MxP-NP, mesenchyme spanning maxillary prominence to nasal pits; FNM, frontonasal mass mesenchyme; mes, mesenchyme; ecto, ectoderm. Figure created in BioRender. Kyomen, S. (2026) https://BioRender.com/g01q360

Supplementary Table 1. Craniofacial phenotypes associated with members of FGF, Hh, TGF-ß/BMP, WNT and NOTCH signaling pathways. Gene names are according to mouse nomenclature.

Pathway	Gene	Main molecular role in pathway	Craniofacial phenotypes	Observations	References
FGF	*Fgf10*	Ligand	Cleft in secondary palate in homozygous null mutant mice.	*Shh* expression in the palatal epithelium is a downstream target of FGF10 signaling. NOTCH ligand *Jag2* is downregulated in the palatal epithelium in the FGF10 mutant mouse.	(*97*, *98*)
FGF	*Fgf8*	Ligand	First pharyngeal arch malformations such as agnathia in *Fgf8;Nes-cre* mice. Abnormal cartilage development in *Fgf8a* homo- and heterozygous mutants in zebrafish larvae.	More examples in Yin et al.	(*99–102*)
FGF	*Fgfr1*	Receptor	Associated with Crouzon, Pfeiffer and Kallmann syndromes in humans. Cleft lip and palate, micrognathia and	Ablation of *Fgfr1* affected BMP and WNT signaling in E10.5 embryos.	(*103–105*)

			tongue malformation in conditional knock-out mutants in mice.		
FGF	*Fgfr2*	Receptor	Associated with Beare-Stevenson cutis gyrata, Bent Bone Dysplasia, Crouzon and Apert syndromes in humans. Craniofacial hypoplasia and cleft palate as a result of conditional overexpression of *Fgfr2c* in mutant mice.	More examples in Azoury et al.	(*106–108*)
Hh	*Gas1*	Co-receptor	Midline defects, midfacial hypoplasia, incisor abnormalities, and cleft palate in homozygous null mutant mice.	Loss of *Shh* expression in the forebrain. Integrates NOTCH and Hh signaling in neuroepithelium. Reduction of *Ptch1* expression in the palatal mesenchyme in mutant mice.	(*109*, *110*)
Hh	*Ptch1*	Receptor	Associated with basal cell nevus syndrome in humans.		(*111–113*)
Hh	*Shh*	Ligand	Associated with holoprosencephaly and fetal alcohol syndrome		(*114–120*)

			in humans. Midline malformation, hypertelorism, abnormal forebrain morphology, bilateral asymmetry, arrested tooth development, and presence of a frontal proboscis in mutant mice.		
Hh	*Smo*	Co-receptor	Cyclopia and bilateral asymmetry in mutant mice. Disrupted chondrogenesis in mutant zebrafish.	Associated with meningioma formation in humans. Reduced expression of *Nodal* in Smo mutant mice.	(*114*, *121–123*)
Hh	*Sufu*	Regulator	Exencephaly, truncated mandible, absence of eyes, and cleft lip in mutant mice.		(*124*)
NOTCH	*Dll3*	Ligand	Mandibular and palatal malformations in mutant mice.	Associated with axial skeleton malformations in humans.	(*125*, *126*)
NOTCH	*Jag1*	Ligand	Mutant mice recapitulate human Alagille syndrome phenotypes, specifically midface hypoplasia.	Associated with osteoporosis in humans.	(*127*, *128*)

NOTCH	*Jag2*	Ligand	Cleft in secondary palate, fusion of the tongue with palatal shelves in mutant mice.	Homozygous mice mutant mice also display syndactyly.	(*47*, *129*)
NOTCH	*Notch1*	Receptor	NOTCH1 mutations are associated with Adams-Oliver syndrome, causing scalp malformation in humans.		(*130*)
NOTCH	*Notch2*	Receptor	Mutation associated with Hadju-Cheney syndrome causing dysmorphic face, aplasia of facial sinuses and abnormalities in teeth development in humans.		(*131–133*)
TGF-ß/BMP	*Acvr2a*	Receptor	Defect in neural crest-derived cartilage and bone in zebrafish morphants. Micrognathia, dysmorphic Meckel's cartilage and cleft palate in mutant mice.		(*134*, *135*)
TGF-ß/BMP	*Alk2*	Receptor	Cleft palate, mandible malformation, impaired skull ossification, impaired chondrogenesis and		(*136*, *137*)

			dysmorphic Meckel's cartilage in mutant mice.		
TGF-ß/BMP	*Axin1/2*	Regulators	Malformation in the temporomandibular joint, accelerated chondrocyte hypertrophy, snout truncation and premature closure of cranial sutures in mutant mice.	Negative regulators of the WNT pathway.	(*138*, *139*)
TGF-ß/BMP	*Bmp2*	Ligand	Malformation in cranial neural crest-derived bones when deleted in the *Bmp4* conditional knockout background in mice.		(*140*)
TGF-ß/BMP	*Bmp4*	Ligand	Hypoplasia of maxilla and mandible, cleft lip and palate, and arrested tooth development in mutant mice.		(*140–142*)
TGF-ß/BMP	*Bmp7*	Ligand	Associated with bilateral anophthalmia, microphthalmia, and cleft palate in humans. Impaired elevation of palatal shelves, cleft palate, tooth malformation and		(*143–145*)

			mandible dysplasia in mutant mice.		
TGF-ß/BMP	*Bmpr1a/b*	Receptors	Mutations associated with Pierre Robin syndrome in humans. Mandible malformation, orbital hypertelorism, craniosynostosis, dome-shaped skull in mutant mice.	Mutant mice also presented limb malformations.	(*146–148*)
TGF-ß/BMP	*Smad2*	Mediator	Cyclopia, severe rostral truncation and mandible malformation were observed in mutant mice when crossed with *Nodal*.		(*149*)
WNT	*Dkk1*	Antagonist	Severe head truncation, absence of nasal, mandibular and maxillary bones in mutant mice. Overexpression leads to severe molar defects.	Mutant mice also presented limb malformations.	(*150*, *151*)
WNT	*Dvl1*	Mediator	Mutations are associated with Robinow syndrome in humans.		(*152–154*)

WNT	*Fzd1/2*	Receptors	Mutations associated with Robinow syndrome in humans. Cleft palate, wider roof of the mouth and absence of palatine bones in the mutant mouse.	Mutant mice also presented limb malformations.	(*155–158*)
WNT	*Lef1*	Mediator	Associated with ectodermal dysplasia in humans. Arrested tooth development at bud stage, lack of body hair and vibrissae in mutant mice.		(*159–161*)
WNT	*Lrp5*	Receptor	Associated with craniosynostosis in humans. Mild effect in craniofacial phenotype in mutant mice.	Mouse model for Apert syndrome has higher expression of *Lrp5/6* in coronal sutures.	(*162–164*)
WNT	*Lrp6*	Receptor	Associated with cleft palate and cleft lip, microdontia, root malformation and fusion of teeth in humans.	Mouse model for Apert syndrome has higher expression of *Lrp5/6* in coronal sutures.	(*165*, *166*)
WNT	*Ror2*	Receptor	Associated with Pierre Robinow syndrome in humans and beak length in pigeons. Midface hypoplasia,	Mutant mice also presented limb malformations.	(*167–169*)

			hypertelorism, oral abnormalities, truncated nasal capsule and delayed ossification of the maxilla in mutant mice.		
WNT	*Wnt10a/b*	Ligand	In humans, related to dental abnormalities, tooth agenesis and syndromes causing ectodermal dysplasia. Tooth agenesis, enamel hypoplasia and root malformation observed in mutant mice.		(*170–173*)
WNT	*Wnt11*	Ligand	Overexpression by viral infection in chicken caused beak malformation, cleft palate and bone defects. Loss-of-function in zebrafish led to cyclopia and midline defects.		(*174*, *175*)
WNT	*Wnt3/3a*	Ligand	Associated with Tetra-Amelia syndrome and non-syndromic cleft lip and palate.		(*176*, *177*)
WNT	*Wnt5a*	Ligand	Associated with Pierre Robinow syndrome in humans. Cleft palate		(*67*, *178–180*)

			and truncation of upper and lower jaws in mutant mice.		
WNT	*Wnt6*	Ligand	Wnt6-Wnt10a region contains cleft palate-associated SNPs in humans.		(*181*)
WNT	*Wnt7a*	Ligand	Mutation associated with Al-Awadi-Raas-Rothschild syndrome in humans.		(*182*, *183*)
WNT	*Wnt9b*	Ligand	Delayed outgrowth of frontonasal prominences, midline malformations, cleft palate and lip in mutant mice.		(*75*, *184*)
WNT, Hh	*Gsk3b*	Regulator	Cleft palate, delayed ossification of the skull, ear bones and cranial base in homozygous null mutant mice.	Mutants also presented impaired fusion of ribs and bifid sternum.	(*185*)

Supplementary Table 2. Morphological comparison of mouse and chicken embryos.

	Mouse (E11.5)	Chicken (HH22)	References
**Facial morphology**	The medial nasal prominences (MNP) are formed and positioned along the midline, beginning to contact the maxillary prominences (MXP) on either side of the developing face. The lambdoidal junction, where the MNP, lateral nasal prominence (LNP), and MXP meet, is established. The convergence of the MNP at the midline, along with their inward extension into the oral cavity, gives rise to the primary palate. By this stage, the nasal pits are well-formed. Mandibular prominence (MDP) is well pronounced.	The frontonasal mass (FNM), lateral nasal prominence (LNP), and maxillary prominence (MXP) are well defined. The globular process, the region where the LNP and MXP will eventually fuse, is actively forming and aligning. The FNM appears as a flat structure positioned above the stomodeum. The nasal pits deepen and project ventrally, shifting laterally due to continued expansion of the forebrain. Mandibular prominence (MDP) is well pronounced.	(*186*, *187*, *36*)
**Limb morphology**	Limb buds are paddle-shaped and undivided, showing early signs of skeletal condensations. Apical ectodermal ridge is visible. Onset of *Sox9* expression in the limb bud	Limb buds are paddle-shaped and undivided, showing early signs of skeletal condensations. Apical ectodermal ridge is visible. Onset of *SOX9* expression in the limb bud	(*188*, *189*)

	mesenchyme occurs at E10.5.	mesenchyme is around stage HH22.	
**Heart morphology**	Muscular septum initiates formation. Progressive septation of the outflow tract, the atria and ventricles. Endocardial cushions are well-developed. Ventricular trabeculation, valve and epicardium formation start to take place. Migration of epicardial cells around the heart is complete.	Progressive development of the primitive atrium and primitive ventricle. Endocardial cushions have formed in the outflow tract and atrioventricular canal junction. Migration of epicardial cells around the heart is complete.	(*190–193*)
**Brain morphology**	Forebrain, midbrain and hindbrain are regionalized, with clear anatomical boundaries. Hippocampal neurons begin to develop. Neural tube is fully closed.	Forebrain, midbrain and hindbrain are regionalized, with clear anatomical boundaries. Neural tube is fully closed.	(*194–196*)
***Shh* expression domain in the face**	*Shh* has paired bilateral expression domains in the ectoderm of the ventral MNP, extending dorsally from the stomodeum toward the MXP. Small expression domain in the ventral nasal pit rim.	*SHH* is expressed in the oral ectoderm following the ventral FNM, extending dorsally from the stomodeum toward the MXP.	(*34*, *197*, *35*, *2*), this work
***Fgf8* expression domain in the face**	*Fgf8* is expressed in the ectoderm surrounding the nasal pits, as well as in the	*FGF8* is expressed in the surface ectoderm, the oral ectoderm, around the nasal	(*34*, *197*, *35*, *2*), this work

	oral ectoderm and along the ventral region of the MXP.	pits, and along the ventral region of the MXP.	
***Bmp4* expression domain in the face**	In the ectoderm, *Bmp4* is expressed in the oral region, partially overlapping with *Shh* and *Fgf8* expression in FEZ. *Bmp4* is also expressed in the mesenchyme of frontonasal prominences, especially around the nasal pits and in the rostral part of the MDP.	In the ectoderm, *BMP4* is expressed in the oral region, partially overlapping with *SHH* and *FGF8* expression in FEZ. In the mesenchyme, the gene is expressed in the FNM, LNP and MXP.	(*61*, *197*), this work
***Wnt5a/6* expression domains in the face**	*Wnt5a* is expressed in the mesenchyme of LNP, MNP, MXP and the midline of MDP. The gene is also expressed in the ectoderm covering the rim of nasal pits. *Wnt6* is expressed in the surface ectoderm of frontonasal prominences.	*WNT5A* is expressed in the mesenchyme of LNP, FNM, MXP and midline of MDP. *WNT6* is expressed in the surface ectoderm of frontonasal prominences.	(*198*, *199*), this work

Description of Supplementary Tables 3-7 provided as separate files

Supplementary Table 3. Marker genes used for cluster annotation related to Figure 1.

Sheet 1. Marker genes from all clusters mouse E11.5 scRNA-seq.

Sheet 2. Marker genes from facial clusters mouse E11.5 scRNA-seq.

Sheet 3. Marker genes from all clusters chicken HH22 scRNA-seq.

Sheet 4. Marker genes from facial clusters chicken HH22 scRNA-seq.

Sheet 5. Summary of marker genes of main facial populations in mouse and chicken.

Sheet 6. Information on *in situ* HCR probes.

Sheet 7. Alignment scores obtained by SAMap.

Sheet 8. List of one-to-one orthologs (mouse-chicken) obtained by SAMap.

Supplementary Table 4. Statistics and scATAC-seq peaks related to Figure 4 and Supplementary Figure 13.

Sheet 1. Proportion and annotation of all peaks from mouse scATAC-sea dataset.

Sheet 2. Proportion and annotation of all peaks from chicken scATAC-sea dataset.

Sheet 3. Coordinates of DARs found in mouse scATAC-seq dataset related to Figure 3.

Sheet 4. Coordinates of DARs found in chicken scATAC-seq dataset related to Figure 3.

Sheet 5. Annotation of mouse DARs.

Sheet 6. Annotation of chicken DARs.

Sheet 7. Summary mouse-chicken IPP.

Sheet 8. Summary chicken-mouse IPP.

Sheet 9. Mouse DARs functionally conserved.

Sheet 10. Chicken DARs functionally conserved.

Sheet 11. Coordinates of conserved DARs from ectodermal clusters used for Gene Ontology enrichment.

Sheet 12. Coordinates of conserved DARs from mesenchymal clusters used for Gene Ontology enrichment.

Sheet 13. Bridging species used for IPP.

Sheet 14. Proportion test results for peak conservation.

Sheet 15. Data for IPP mouse-chicken all peaks.

Sheet 16. Data for IPP mouse-chicken unmapped peaks.

Sheet 17. Data for IPP chicken-mouse all peaks.

Sheet 18. Data for IPP chicken-mouse unmapped peaks.

Sheet 19. Data for IPP *in vivo* enhancers.

Supplementary Table 5. ChIP-seq (H3K27ac) in chicken HH22 facial prominences.

Sheet 1. Quality control statistics of the ChIP-seq data.

Sheet 2. Annotated ChIP-seq peaks from chicken HH22 facial prominences.

Sheet 3. ChIP-seq peaks overlapping scATAC-seq peaks in chicken datasets.

Supplementary Table 6. Candidate cis-regulatory elements validated *in vivo*.

Sheet 1. *In vivo* validated enhancers described in the main text.

Sheet 2. *In vivo* validated enhancers from VISTA database that intersect scATAC-seq peaks (E11.5).

Sheet 3. *In vivo* validated enhancers from VISTA database that intersect scATAC-seq DARs (E11.5).

Supplementary Table 7. GWAS enrichment related to Figure 7.

Sheet 1. LD blocks from mouse GWAS from Pallares et al (82).

Sheet 2. LD blocks from Pallares et al (82), liftedOver from mouse mm8 to mm10 genome assembly.

Sheet 3. Craniofacial-trait-associated SNPs in human used for generating LD blocks.

Sheet 4. scATAC-seq peaks liftOver from mouse (mm10) to human (GRCh38).

Sheet 5. Raw LD blocks generated from human craniofacial-trait-associated GWAS data.

Sheet 6. Filtered LD blocks generated from human craniofacial-trait-associated GWAS data.

REFERENCES

1. D. Hu, R. S. Marcucio, Unique organization of the frontonasal ectodermal zone in birds and mammals. *Dev. Biol.* **325**, 200–210 (2009).

2. D. Hu, N. M. Young, X. Li, Y. Xu, B. Hallgrímsson, R. S. Marcucio, A dynamic Shh expression pattern, regulated by SHH and BMP signaling, coordinates fusion of primordia in the amniote face. *Development* **142**, 567–574 (2015).

3. X.-J. Zhu, Y. Liu, X. Yuan, M. Wang, W. Zhao, X. Yang, X. Zhang, W. Hsu, M. Qiu, Z. Zhang, Z. Zhang, Ectodermal Wnt controls nasal pit morphogenesis through modulation of the BMP/FGF/JNK signaling axis. *Dev. Dyn.* **245**, 414–426 (2016).

4. M. Marchini, D. Hu, L. Lo Vercio, N. M. Young, N. D. Forkert, B. Hallgrímsson, R. Marcucio, Wnt signaling drives correlated changes in facial morphology and brain shape. *Front. Cell Dev. Biol.* **9**, 644099 (2021).

5. S. Kyomen, A. P. Murillo-Rincón, M. Kaucká, Evolutionary mechanisms modulating the mammalian skull development. *Philos. Trans. R. Soc. Lond. B Biol. Sci.* **378**, 20220080 (2023).

6. A. Abzhanov, C. J. Tabin, Shh and Fgf8 act synergistically to drive cartilage outgrowth during cranial development. *Dev. Biol.* **273**, 134–148 (2004).

7. S. A. Brugmann, K. E. Powder, N. M. Young, L. H. Goodnough, S. M. Hahn, A. W. James, J. A. Helms, M. Lovett, Comparative gene expression analysis of avian embryonic facial structures reveals new candidates for human craniofacial disorders. *Hum. Mol. Genet.* **19**, 920–930 (2010).

8. S. R. F. Twigg, A. O. M. Wilkie, New insights into craniofacial malformations. *Hum. Mol. Genet.* **24**, R50–R59 (2015).

9. A. O. M. Wilkie, G. M. Morriss-Kay, Genetics of craniofacial development and malformation. *Nat. Rev. Genet.* **2**, 458–468 (2001).

10. R. S. Marcucio, D. R. Cordero, D. Hu, J. A. Helms, Molecular interactions coordinating the development of the forebrain and face. *Dev. Biol.* **284**, 48–61 (2005).

11. D. Hu, R. S. Marcucio, J. A. Helms, A zone of frontonasal ectoderm regulates patterning and growth in the face. *Development* **130**, 1749–1758 (2003).

12. A. Abzhanov, D. R. Cordero, J. Sen, C. J. Tabin, J. A. Helms, Cross-regulatory interactions between Fgf8 and Shh in the avian frontonasal prominence. *Congenit. Anom.* **47**, 136–148 (2007).

13. C. H. Mok, D. Hu, M. Losa, M. Risolino, L. Selleri, R. S. Marcucio, PBX1 and PBX3 transcription factors regulate SHH expression in the Frontonasal Ectodermal Zone through complementary mechanisms. *PLOS Genet*. **21**, e1011315 (2025).

14. N. M. Young, H. J. Chong, D. Hu, B. Hallgrímsson, R. S. Marcucio, Quantitative analyses link modulation of sonic hedgehog signaling to continuous variation in facial growth and shape. *Development.* **137**, 3405–3409 (2010).

15. D. Cordero, R. Marcucio, D. Hu, W. Gaffield, M. Tapadia, J. A. Helms, Temporal perturbations in sonic hedgehog signaling elicit the spectrum of holoprosencephaly phenotypes. *J. Clin. Invest.* **114**, 485–494 (2004).

16. S. S. Rajderkar, K. Paraiso, M. L. Amaral, M. Kosicki, L. E. Cook, F. Darbellay, C. H. Spurrell, M. Osterwalder, Y. Zhu, H. Wu, S. Y. Afzal, M. J. Blow, G. Kelman, I. Barozzi, Y. Fukuda-Yuzawa, J. A. Akiyama, V. Afzal, S. Tran, I. Plajzer-Frick, C. S. Novak, M. Kato, R. D. Hunter, K. von Maydell, A. Wang, L. Lin, S. Preissl, S. Lisgo, B. Ren, D. E. Dickel, L. A. Pennacchio, A. Visel, Dynamic enhancer landscapes in human craniofacial development. *Nat. Commun.* **15**, 2030 (2024).

17. T. N. Yankee, S. Oh, E. W. Winchester, A. Wilderman, K. Robinson, T. Gordon, J. A. Rosenfeld, J. VanOudenhove, D. A. Scott, E. J. Leslie, J. Cotney, Integrative analysis of transcriptome dynamics during human craniofacial development identifies candidate disease genes. *Nat. Commun.* **14**, 4623 (2023).

18. K.-C. Tseng, J. G. Crump, Craniofacial developmental biology in the single-cell era. *Development* **150**, dev202077 (2023).

19. E. R. Brooks, A. R. Moorman, B. Bhattacharya, I. S. Prudhomme, M. Land, H. L. Alcorn, R. Sharma, D. Pe'er, J. A. Zallen, A single-cell atlas of spatial and temporal gene expression in the mouse cranial neural plate. *eLife* **13**, RP102819 (2025).

20. F. Yan, A. Suzuki, C. Iwaya, G. Pei, X. Chen, H. Yoshioka, M. Yu, L. M. Simon, J. Iwata, Z. Zhao, Single-cell multiomics decodes regulatory programs for mouse secondary palate development. *Nat. Commun.* **15**, 821 (2024).

21. H. Li, K. L. Jones, J. E. Hooper, T. Williams, The molecular anatomy of mammalian upper lip and primary palate fusion at single cell resolution. *Development* **146**, dev174888 (2019).

22. Y. H. Ozekin, R. O'Rourke, E. A. Bates, Single cell sequencing of the mouse anterior palate reveals mesenchymal heterogeneity. *Dev. Dyn.* **252**, 713–727 (2023).

23. J. Sun, Y. Lin, N. Ha, J. Zhang, W. Wang, X. Wang, Q. Bian, Single-cell RNA-Seq reveals transcriptional regulatory networks directing the development of mouse maxillary prominence. *J. Genet. Genomics* **50**, 676–687 (2023).

24. H. K. Long, S. L. Prescott, J. Wysocka, Ever-changing landscapes: Transcriptional enhancers in development and evolution. *Cell* **167**, 1170–1187 (2016).

25. P. J. Wittkopp, G. Kalay, Cis-regulatory elements: Molecular mechanisms and evolutionary processes underlying divergence. *Nat. Rev. Genet.* **13**, 59–69 (2012).

26. M. Kaucka, Cis-regulatory landscapes in the evolution and development of the mammalian skull. *Philos. Trans. R. Soc. Lond. B Biol. Sci.* **378**, 20220079 (2023).

27. N. R. Zemke, E. J. Armand, W. Wang, S. Lee, J. Zhou, Y. E. Li, H. Liu, W. Tian, J. R. Nery, R. G. Castanon, A. Bartlett, J. K. Osteen, D. Li, X. Zhuo, V. Xu, L. Chang, K. Dong, H. S. Indralingam, J. A. Rink, Y. Xie, M. Miller, F. M. Krienen, Q. Zhang, N. Taskin, J. Ting, G. Feng, S. A. McCarroll, E. M. Callaway, T. Wang, E. S. Lein, M. M. Behrens, J. R. Ecker, B.

Ren, Conserved and divergent gene regulatory programs of the mammalian neocortex. *Nature* **624**, 390–402 (2023).

28. S. L. Prescott, R. Srinivasan, M. C. Marchetto, I. Grishina, I. Narvaiza, L. Selleri, F. H. Gage, T. Swigut, J. Wysocka, Enhancer divergence and cis-regulatory evolution in the human and chimp neural crest. *Cell* **163**, 68–83 (2015).

29. H. K. Zieger, L. Weinhold, A. Schmidt, M. Holtgrewe, S. A. Juranek, A. Siewert, A. B. Scheer, F. Thieme, E. Mangold, N. Ishorst, F. U. Brand, J. Welzenbach, D. Beule, K. Paeschke, P. M. Krawitz, K. U. Ludwig, Prioritization of non-coding elements involved in non-syndromic cleft lip with/without cleft palate through genome-wide analysis of de novo mutations. *HGG Adv.* **4**, 100166 (2023).

30. L. A. Lettice, S. J. H. Heaney, L. A. Purdie, L. Li, P. de Beer, B. A. Oostra, D. Goode, G. Elgar, R. E. Hill, E. de Graaff, A long-range Shh enhancer regulates expression in the developing limb and fin and is associated with preaxial polydactyly. *Hum. Mol. Genet.* **12**, 1725–1735 (2003).

31. H. K. Long, M. Osterwalder, I. C. Welsh, K. Hansen, J. O. J. Davies, Y. E. Liu, M. Koska, A. T. Adams, R. Aho, N. Arora, K. Ikeda, R. M. Williams, T. Sauka-Spengler, M. H. Porteus, T. Mohun, D. E. Dickel, T. Swigut, J. R. Hughes, D. R. Higgs, A. Visel, L. Selleri, J. Wysocka, Loss of extreme long-range enhancers in human neural crest drives a craniofacial disorder. *Cell Stem Cell* **27**, 765–783.e14 (2020).

32. E. Smith, A. Shilatifard, Enhancer biology and enhanceropathies. *Nat. Struct. Mol. Biol.* **21**, 210–219 (2014).

33. F. Rahimov, M. L. Marazita, A. Visel, M. E. Cooper, M. J. Hitchler, M. Rubini, F. E. Domann, M. Govil, K. Christensen, C. Bille, M. Melbye, A. Jugessur, R. T. Lie, A. J. Wilcox, D. R. Fitzpatrick, E. D. Green, P. A. Mossey, J. Little, R. P. Steegers-Theunissen, L. A. Pennacchio, B. C. Schutte, J. C. Murray, Disruption of an AP-2α binding site in an IRF6 enhancer is associated with cleft lip. *Nat. Genet.* **40**, 1341–1347 (2008).

34. D. Hu, N. M. Young, Q. Xu, H. Jamniczky, R. M. Green, W. Mio, R. S. Marcucio, B. Hallgrimsson, Signals from the brain induce variation in avian facial shape. *Dev. Dyn.* **244**, 1133–1143 (2015).

35. H. Kurosaka, The roles of hedgehog signaling in upper lip formation. *Biomed. Res. Int.* **2015**, e901041 (2015).

36. J. Lu, B. Peng, W. Wang, Y. Zou, Epithelial-mesenchymal crosstalk: The scriptwriter of craniofacial morphogenesis. *Front. Cell Dev. Biol.* **12**, 1497002 (2024).

37. P. B. Antin, T. A. Yatskievych, S. Davey, D. K. Darnell, GEISHA: An evolving gene expression resource for the chicken embryo. *Nucleic Acids Res.* **42**, D933–D937 (2014).

38. A. Visel, C. Thaller, G. Eichele, GenePaint.org: An atlas of gene expression patterns in the mouse embryo. *Nucleic Acids Res.* **32**, D552–D556 (2004).

39. L. Richardson, S. Venkataraman, P. Stevenson, Y. Yang, J. Moss, L. Graham, N. Burton, B. Hill, J. Rao, R. A. Baldock, C. Armit, EMAGE mouse embryo spatial gene expression database: 2014 update. *Nucleic Acids Res.* **42**, D835–D844 (2014).

40. J. Abramyan, J. M. Richman, Craniofacial development: Discoveries made in the chicken embryo. *Int. J. Dev. Biol.* **62**, 97–107 (2018).

41. S. A. Brugmann, M. D. Tapadia, J. A. Helms, "The molecular origins of species-specific facial pattern," in *Current Topics in Developmental Biology* (Academic Press, 2006), vol. 73, pp. 1–42.

42. A. J. Tarashansky, J. M. Musser, M. Khariton, P. Li, D. Arendt, S. R. Quake, B. Wang, Mapping single-cell atlases throughout Metazoa unravels cell type evolution. *eLife* **10**, e66747 (2021).

43. S. Jin, C. F. Guerrero-Juarez, L. Zhang, I. Chang, R. Ramos, C.-H. Kuan, P. Myung, M. V. Plikus, Q. Nie, Inference and analysis of cell-cell communication using CellChat. *Nat. Commun.* **12**, 1088 (2021).

44. H.-J. Kim, D. P. C. Rice, P. J. Kettunen, I. Thesleff, FGF-, BMP- and Shh-mediated signalling pathways in the regulation of cranial suture morphogenesis and calvarial bone development. *Development* **125**, 1241–1251 (1998).

45. H. L. Szabo-Rogers, P. Geetha-Loganathan, C. J. Whiting, S. Nimmagadda, K. Fu, J. M. Richman, Novel skeletogenic patterning roles for the olfactory pit. *Development* **136**, 219–229 (2009).

46. H. L. Szabo-Rogers, P. Geetha-Loganathan, S. Nimmagadda, K. K. Fu, J. M. Richman, FGF signals from the nasal pit are necessary for normal facial morphogenesis. *Dev. Biol.* **318**, 289–302 (2008).

47. L. M. Casey, Y. Lan, E.-S. Cho, K. M. Maltby, T. Gridley, R. Jiang, Jag2-Notch1 signaling regulates oral epithelial differentiation and palate development. *Dev. Dyn.* **235**, 1830–1844 (2006).

48. M. Marchini, G. Keller, N. Khan, R. Shah, A. Saliceti Galarza, K. B. Starr, A. Apostopoulos, T. J. Sanger, Sonic hedgehog and fibroblast growth factor 8 regulate the evolution of amniote facial proportions. *Commun. Biol.* **8**, 84 (2025).

49. T. J. Sanger, J. B. Losos, J. J. Gibson-Brown, A developmental staging series for the lizard genus *Anolis*: A new system for the integration of evolution, development, and ecology. *J. Morphol.* **269**, 129–137 (2008).

50. M. R. Corces, J. D. Buenrostro, B. Wu, P. G. Greenside, S. M. Chan, J. L. Koenig, M. P. Snyder, J. K. Pritchard, A. Kundaje, W. J. Greenleaf, R. Majeti, H. Y. Chang, Lineage-specific and single-cell chromatin accessibility charts human hematopoiesis and leukemia evolution. *Nat. Genet.* **48**, 1193–1203 (2016).

51. J. M. Granja, M. R. Corces, S. E. Pierce, S. T. Bagdatli, H. Choudhry, H. Y. Chang, W. J. Greenleaf, ArchR is a scalable software package for integrative single-cell chromatin accessibility analysis. *Nat. Genet.* **53**, 403–411 (2021).

52. H. E. Arda, J. Tsai, Y. R. Rosli, P. Giresi, R. Bottino, W. J. Greenleaf, H. Y. Chang, S. K. Kim, A chromatin basis for cell lineage and disease risk in the human pancreas. *Cell Syst.* **7**, 310–322.e4 (2018).

53. P. A. Alexandre, M. Naval-Sánchez, M. Menzies, L. T. Nguyen, L. R. Porto-Neto, M. R. S. Fortes, A. Reverter, Chromatin accessibility and regulatory vocabulary across indicine cattle tissues. *Genome Biol.* **22**, 273 (2021).

54. B. Borsari, P. Villegas-Mirón, S. Pérez-Lluch, I. Turpin, H. Laayouni, A. Segarra-Casas, J. Bertranpetit, R. Guigó, S. Acosta, Enhancers with tissue-specific activity are enriched in intronic regions. *Genome Res.* **31**, 1325–1336 (2021).

55. A. P. Murillo-Rincón, L. W. G. Seton, E. Escamilla-Vega, A. Damatac, J. Fuß, C. Fortmann-Grote, M. Kaucká, Positional programs in early murine facial development and their role in human facial shape variability. *Nat. Commun.* **16**, 10112 (2025).

56. M. H. Q. Phan, T. Zehnder, F. Puntieri, A. Magg, B. Majchrzycka, M. Antonović, H. Wieler, B.-W. Lo, D. Baranasic, B. Lenhard, F. Müller, M. Vingron, D. M. Ibrahim, Conservation of regulatory elements with highly diverged sequences across large evolutionary distances. *Nat. Genet.* **57**, 1524–1534 (2025).

57. C. Y. McLean, D. Bristor, M. Hiller, S. L. Clarke, B. T. Schaar, C. B. Lowe, A. M. Wenger, G. Bejerano, GREAT improves functional interpretation of cis-regulatory regions. *Nat. Biotechnol.* **28**, 495–501 (2010).

58. M. Kosicki, F. A. Baltoumas, G. Kelman, J. Boverhof, Y. Ong, L. E. Cook, D. E. Dickel, G. A. Pavlopoulos, L. A. Pennacchio, A. Visel, VISTA Enhancer browser: An updated database of tissue-specific developmental enhancers. *Nucleic Acids Res.* **53**, D324–D330 (2025).

59. R. Ramani, K. Krumholz, Y.-F. Huang, A. Siepel, PhastWeb: A web interface for evolutionary conservation scoring of multiple sequence alignments using phastCons and phyloP. *Bioinformatics* **35**, 2320–2322 (2019).

60. E. Anderson, P. S. Devenney, R. E. Hill, L. A. Lettice, Mapping the Shh long-range regulatory domain. *Development* **141**, 3934–3943 (2014).

61. D. Jumlongras, S. A. Lachke, D. J. O'Connell, A. Aboukhalil, X. Li, S. E. Choe, J. W. K. Ho, A. Turbe-Doan, E. A. Robertson, B. R. Olsen, M. L. Bulyk, B. A. Amendt, R. L. Maas, An evolutionarily conserved enhancer regulates Bmp4 expression in developing incisor and limb bud. *PLOS ONE* **7**, e38568 (2012).

62. C. Attanasio, A. S. Nord, Y. Zhu, M. J. Blow, Z. Li, D. K. Liberton, H. Morrison, I. Plajzer-Frick, A. Holt, R. Hosseini, S. Phouanenavong, J. A. Akiyama, M. Shoukry, V. Afzal, E. M. Rubin, D. R. FitzPatrick, B. Ren, B. Hallgrímsson, L. A. Pennacchio, A. Visel, Fine tuning of craniofacial morphology by distant-acting enhancers. *Science* **342**, 1241006 (2013).

63. A. Wilderman, E. D'haene, M. Baetens, T. N. Yankee, E. W. Winchester, N. Glidden, E. Roets, J. Van Dorpe, S. Janssens, D. E. Miller, M. Galey, K. M. Brown, R. W. Stottmann, S. Vergult, K. N. Weaver, S. A. Brugmann, T. C. Cox, J. Cotney, A distant global control region is essential for normal expression of anterior HOXA genes during mouse and human craniofacial development. *Nat. Commun.* **15**, 136 (2024).

64. A. Hörnblad, S. Bastide, K. Langenfeld, F. Langa, F. Spitz, Dissection of the Fgf8 regulatory landscape by in vivo CRISPR-editing reveals extensive intra- and inter-enhancer redundancy. *Nat. Commun.* **12**, 439 (2021).

65. M. Marinić, T. Aktas, S. Ruf, F. Spitz, An integrated holo-enhancer unit defines tissue and gene specificity of the *Fgf8* regulatory landscape. *Dev. Cell* **24**, 530–542 (2013).

66. J. Topczewski, R. M. Dale, B. E. Sisson, Planar cell polarity signaling in craniofacial development. *Organogenesis* **7**, 255–259 (2011).

67. M. Kaucka, E. Ivashkin, D. Gyllborg, T. Zikmund, M. Tesarova, J. Kaiser, M. Xie, J. Petersen, V. Pachnis, S. K. Nicolis, T. Yu, P. Sharpe, E. Arenas, H. Brismar, H. Blom, H. Clevers, U. Suter, A. S. Chagin, K. Fried, A. Hellander, I. Adameyko, Analysis of neural crest–derived clones reveals novel aspects of facial development. *Sci. Adv.* **2**, e1600060 (2016).

68. H. Nishihara, N. Kobayashi, C. Kimura-Yoshida, K. Yan, O. Bormuth, Q. Ding, A. Nakanishi, T. Sasaki, M. Hirakawa, K. Sumiyama, Y. Furuta, V. Tarabykin, I. Matsuo, N. Okada, Coordinately co-opted multiple transposable elements constitute an enhancer for wnt5a expression in the mammalian secondary palate. *PLOS Genet.* **12**, e1006380 (2016).

69. X. Fan, D. Wang, J. E. Burgmaier, Y. Teng, R.-A. Romano, S. Sinha, R. Yi, Single cell and open chromatin analysis reveals molecular origin of epidermal cells of the skin. *Dev. Cell* **47**, 21–37.e5 (2018).

70. J. M. Santos-Pereira, L. Gallardo-Fuentes, A. Neto, R. D. Acemel, J. J. Tena, Pioneer and repressive functions of p63 during zebrafish embryonic ectoderm specification. *Nat. Commun.* **10**, 3049 (2019).

71. T. K. Panaliappan, W. Wittmann, V. K. Jidigam, S. Mercurio, J. A. Bertolini, S. Sghari, R. Bose, C. Patthey, S. K. Nicolis, L. Gunhaga, Sox2 is required for olfactory pit formation and olfactory neurogenesis through BMP restriction and Hes5 upregulation. *Development* **145**, dev153791 (2018).

72. P. Xu, B. Balczerski, A. Ciozda, K. Louie, V. Oralova, A. Huysseune, J. G. Crump, Fox proteins are modular competency factors for facial cartilage and tooth specification. *Development* **145**, dev165498 (2018).

73. Y. Liu, M. Festing, J. C. Thompson, M. Hester, S. Rankin, H. M. El-Hodiri, A. M. Zorn, M. Weinstein, Smad2 and Smad3 coordinately regulate craniofacial and endodermal development. *Dev. Biol.* **270**, 411–426 (2004).

74. S. Lamichhaney, J. Berglund, M. S. Almén, K. Maqbool, M. Grabherr, A. Martinez-Barrio, M. Promerová, C.-J. Rubin, C. Wang, N. Zamani, B. R. Grant, P. R. Grant, M. T. Webster, L. Andersson, Evolution of Darwin's finches and their beaks revealed by genome sequencing. *Nature* **518**, 371–375 (2015).

75. E. Ferretti, B. Li, R. Zewdu, V. Wells, J. M. Hebert, C. Karner, M. J. Anderson, T. Williams, J. Dixon, M. J. Dixon, M. J. Depew, L. Selleri, A conserved Pbx-Wnt-p63-Irf6 regulatory

module controls face morphogenesis by promoting epithelial apoptosis. *Dev. Cell* **21**, 627–641 (2011).

76. F. Liu, S. E. Millar, Wnt/β-catenin signaling in oral tissue development and disease. *J. Dent. Res.* **89**, 318–330 (2010).

77. T. Wilanowski, J. Caddy, S. B. Ting, N. R. Hislop, L. Cerruti, A. Auden, L. Zhao, S. Asquith, S. Ellis, R. Sinclair, J. M. Cunningham, S. M. Jane, Perturbed desmosomal cadherin expression in grainy head-like 1-null mice. *EMBO J.* **27**, 886–897 (2008).

78. C. T. Miller, D. Yelon, D. Y. R. Stainier, C. B. Kimmel, Two endothelin 1 effectors, hand2 and bapx1,pattern ventral pharyngeal cartilage and the jaw joint. *Development* **130**, 1353–1365 (2003).

79. M. Abe, I. Michikami, T. Fukushi, A. Abe, Y. Maeda, T. Ooshima, S. Wakisaka, Hand2 regulates chondrogenesis *in vitro* and *in vivo*. *Bone* **46**, 1359–1368 (2010).

80. L. F. Pallares, B. Harr, L. M. Turner, D. Tautz, Use of a natural hybrid zone for genomewide association mapping of craniofacial traits in the house mouse. *Mol. Ecol.* **23**, 5756–5770 (2014).

81. Z. Xiong, G. Dankova, L. J. Howe, M. K. Lee, P. G. Hysi, M. A. de Jong, G. Zhu, K. Adhikari, D. Li, Y. Li, B. Pan, E. Feingold, M. L. Marazita, J. R. Shaffer, K. McAloney, S.-H. Xu, L. Jin, S. Wang, F. M. de Vrij, B. Lendemeijer, S. Richmond, A. Zhurov, S. Lewis, G. C. Sharp, L. Paternoster, H. Thompson, R. Gonzalez-Jose, M. C. Bortolini, S. Canizales-Quinteros, C. Gallo, G. Poletti, G. Bedoya, F. Rothhammer, A. G. Uitterlinden, M. A. Ikram, E. Wolvius, S. A. Kushner, T. E. Nijsten, R.-J. T. Palstra, S. Boehringer, S. E. Medland, K. Tang, A. Ruiz-Linares, N. G. Martin, T. D. Spector, E. Stergiakouli, S. M. Weinberg, F. Liu, M. Kayser, International Visible Trait Genetics (VisiGen) Consortium, Novel genetic loci affecting facial shape variation in humans. *eLife* **8**, e49898 (2019).

82. P. Claes, J. Roosenboom, J. D. White, T. Swigut, D. Sero, J. Li, M. K. Lee, A. Zaidi, B. C. Mattern, C. Liebowitz, L. Pearson, T. González, E. J. Leslie, J. C. Carlson, E. Orlova, P. Suetens, D. Vandermeulen, E. Feingold, M. L. Marazita, J. R. Shaffer, J. Wysocka, M. D.

Shriver, S. M. Weinberg, Genome-wide mapping of global-to-local genetic effects on human facial shape. *Nat. Genet.* **50**, 414–423 (2018).

83. Y. G. Tak, P. J. Farnham, Making sense of GWAS: Using epigenomics and genome engineering to understand the functional relevance of SNPs in non-coding regions of the human genome. *Epigenetics Chromatin* **8**, 57 (2015).

84. C. Berthelot, D. Villar, J. E. Horvath, D. T. Odom, P. Flicek, Complexity and conservation of regulatory landscapes underlie evolutionary resilience of mammalian gene expression. *Nat. Ecol. Evol.* **2**, 152–163 (2018).

85. I. S. Peter, E. H. Davidson, Assessing regulatory information in developmental gene regulatory networks. *Proc. Natl. Acad. Sci. U.S.A.* **114**, 5862–5869 (2017).

86. M. Rebeiz, M. Tsiantis, Enhancer evolution and the origins of morphological novelty. *Curr. Opin. Genet. Dev.* **45**, 115–123 (2017).

87. T. P. Yamaguchi, A. Bradley, A. P. McMahon, S. Jones, A Wnt5a pathway underlies outgrowth of multiple structures in the vertebrate embryo. *Development* **126**, 1211–1223 (1999).

88. N. Hecker, N. Kempynck, D. Mauduit, D. Abaffyová, R. Vandepoel, S. Dieltiens, L. Borm, I. Sarropoulos, C. B. González-Blas, J. De Man, K. Davie, E. Leysen, J. Vandensteen, R. Moors, G. Hulselmans, L. Lim, J. De Wit, V. Christiaens, S. Poovathingal, S. Aerts, Enhancer-driven cell type comparison reveals similarities between the mammalian and bird pallium. *Science* **387**, eadp3957 (2025).

89. V. Hamburger, H. L. Hamilton, A series of normal stages in the development of the chick embryo. 1951. *Dev. Dyn.* **195**, 231–272 (1992).

90. S. L. Wolock, R. Lopez, A. M. Klein, Scrublet: Computational identification of cell doublets in single-cell transcriptomic data. *Cell Syst.* **8**, 281–291.e9 (2019).

91. Y. Zhang, T. Liu, C. A. Meyer, J. Eeckhoute, D. S. Johnson, B. E. Bernstein, C. Nusbaum, R. M. Myers, M. Brown, W. Li, X. S. Liu, Model-based analysis of ChIP-Seq (MACS). *Genome Biol.* **9**, R137 (2008).

92. D. van Dijk, R. Sharma, J. Nainys, K. Yim, P. Kathail, A. J. Carr, C. Burdziak, K. R. Moon, C. L. Chaffer, D. Pattabiraman, B. Bierie, L. Mazutis, G. Wolf, S. Krishnaswamy, D. Pe'er, Recovering gene interactions from single-cell data using data diffusion. *Cell* **174**, 716–729.e27 (2018).

93. J. T. Robinson, D. Turner, N. C. Durand, H. Thorvaldsdóttir, J. P. Mesirov, E. L. Aiden, Juicebox.js provides a cloud-based visualization system for Hi-C data. *Cell Syst.* **6**, 256–258.e1 (2018).

94. M. Byrska-Bishop, U. S. Evani, X. Zhao, A. O. Basile, H. J. Abel, A. A. Regier, A. Corvelo, W. E. Clarke, R. Musunuri, K. Nagulapalli, S. Fairley, A. Runnels, L. Winterkorn, E. Lowy, Human Genome Structural Variation Consortium, P. Flicek, S. Germer, H. Brand, I. M. Hall, M. E. Talkowski, G. Narzisi, M. C. Zody, High-coverage whole-genome sequencing of the expanded 1000 Genomes Project cohort including 602 trios. *Cell* **185**, 3426–3440.e19 (2022).

95. M. Osterwalder, S. Tran, R. D. Hunter, E. M. Meky, K. von Maydell, A. N. Harrington, J. Godoy, C. S. Novak, I. Plajzer-Frick, Y. Zhu, J. A. Akiyama, V. Afzal, E. Z. Kvon, L. A. Pennacchio, D. E. Dickel, A. Visel, Characterization of mammalian in vivo enhancers using mouse transgenesis and CRISPR genome editing. *Methods Mol. Biol.* **2403**, 147–186 (2022).

96. K. Becker, N. Jährling, S. Saghafi, R. Weiler, H.-U. Dodt, Chemical clearing and dehydration of GFP expressing mouse brains. *PLOS ONE* **7**, e33916 (2012).

97. S. R. Alappat, Z. Zhang, K. Suzuki, X. Zhang, H. Liu, R. Jiang, G. Yamada, Y. Chen, The cellular and molecular etiology of the cleft secondary palate in Fgf10 mutant mice. *Dev. Biol.* **277**, 102–113 (2005).

98. R. Rice, B. Spencer-Dene, E. C. Connor, A. Gritli-Linde, A. P. McMahon, C. Dickson, I. Thesleff, D. P. C. Rice, Disruption of Fgf10/Fgfr2b-coordinated epithelial-mesenchymal interactions causes cleft palate. *J. Clin. Invest.* **113**, 1692–1700 (2004).

99. D. U. Frank, L. K. Fotheringham, J. A. Brewer, L. J. Muglia, M. Tristani-Firouzi, M. R. Capecchi, A. M. Moon, An Fgf8 mouse mutant phenocopies human 22q11 deletion syndrome. *Development* **129**, 4591–4603 (2002).

100. I. G. E. Gebuijs, S. T. Raterman, J. R. Metz, L. Swanenberg, J. Zethof, R. Van den Bos, C. E. L. Carels, F. A. D. T. G. Wagener, J. W. Von den Hoff, Fgf8a mutation affects craniofacial development and skeletal gene expression in zebrafish larvae. *Biol. Open* **8**, bio039834 (2019).

101. A. Trumpp, M. J. Depew, J. L. R. Rubenstein, J. M. Bishop, G. R. Martin, Cre-mediated gene inactivation demonstrates that FGF8 is required for cell survival and patterning of the first branchial arch. *Genes Dev.* **13**, 3136–3148 (1999).

102. H. Yin, L. Duan, Z. Wang, L. Liu, J. Shen, Fibroblast growth factor 8: Multifaceted role in development and developmental disorder. *Genes Dis.* **12**, 101524 (2025).

103. M. Muenke, U. Schell, A. Hehr, N. H. Robin, H. W. Losken, A. Schinzel, L. J. Pulleyn, P. Rutland, W. Reardon, S. Malcolm, R. M. Winter, A common mutation in the fibroblast growth factor receptor 1 gene in Pfeiffer syndrome. *Nat. Genet.* **8**, 269–274 (1994).

104. N. Pitteloud, J. S. Acierno, A. Meysing, A. V. Eliseenkova, J. Ma, O. A. Ibrahimi, D. L. Metzger, F. J. Hayes, A. A. Dwyer, V. A. Hughes, M. Yialamas, J. E. Hall, E. Grant, M. Mohammadi, W. F. Crowley, Mutations in fibroblast growth factor receptor 1 cause both Kallmann syndrome and normosmic idiopathic hypogonadotropic hypogonadism. *Proc. Natl. Acad. Sci. U.S.A.* **103**, 6281–6286 (2006).

105. C. Wang, J. Y. F. Chang, C. Yang, Y. Huang, J. Liu, P. You, W. L. McKeehan, F. Wang, X. Li, Type 1 fibroblast growth factor receptor in cranial neural crest cell-derived mesenchyme is required for palatogenesis. *J. Biol. Chem.* **288**, 22174–22183 (2013).

106. S. C. Azoury, S. Reddy, V. Shukla, C.-X. Deng, Fibroblast growth factor receptor 2 (FGFR2) mutation related syndromic craniosynostosis. *Int. J. Biol. Sci.* **13**, 1479–1488 (2017).

107. K. A. Przylepa, W. Paznekas, M. Zhang, M. Golabi, W. Bias, M. J. Bamshad, J. C. Carey, B. D. Hall, R. Stevenson, S. J. Orlow, M. M. Cohen Jr., E. W. Jabs, Fibroblast growth factor receptor 2 mutations in Beare–Stevenson cutis gyrata syndrome. *Nat. Genet.* **13**, 492–494 (1996).

108. K. K. L. Lee, E. Peskett, C. M. Quinn, R. Aiello, L. Adeeva, D. A. Moulding, P. Stanier, E. Pauws, Overexpression of Fgfr2c causes craniofacial bone hypoplasia and ameliorates craniosynostosis in the Crouzon mouse. *Dis. Model. Mech.* **11**, dmm035311 (2018).

109. M. Marczenke, D. Y. Sunaga-Franze, O. Popp, I. W. Althaus, S. Sauer, P. Mertins, A. Christ, B. L. Allen, T. E. Willnow, GAS1 is required for NOTCH-dependent facilitation of SHH signaling in the ventral forebrain neuroepithelium. *Development* **148**, dev200080 (2021).

110. M. Seppala, M. J. Depew, D. C. Martinelli, C.-M. Fan, P. T. Sharpe, M. T. Cobourne, Gas1 is a modifier for holoprosencephaly and genetically interacts with sonic hedgehog. *J. Clin. Invest.* **117**, 1575–1584 (2007).

111. Y. Murata, H. Kurosaka, Y. Ohata, T. Aikawa, S. Takahata, K. Fujii, T. Miyashita, C. Morita, T. Inubushi, T. Kubota, N. Sakai, K. Ozono, M. Kogo, T. Yamashiro, A novel PTCH1 mutation in basal cell nevus syndrome with rare craniofacial features. *Hum. Genome Var.* **6**, 16 (2019).

112. R. L. Johnson, A. L. Rothman, J. Xie, L. V. Goodrich, J. W. Bare, J. M. Bonifas, A. G. Quinn, R. M. Myers, D. R. Cox, E. H. Epstein, M. P. Scott, Human homolog of patched, a candidate gene for the basal cell nevus syndrome. *Science* **272**, 1668–1671 (1996).

113. W. Feng, I. Choi, D. E. Clouthier, L. Niswander, T. Williams, The Ptch1(DL) mouse: A new model to study lambdoid craniosynostosis and basal cell nevus syndrome-associated skeletal defects. *Genesis* **51**, 677–689 (2013).

114. J. Abramyan, Hedgehog signaling and embryonic craniofacial disorders. *J. Dev. Biol.* **7**, 9 (2019).

115. D. Hu, J. A. Helms, The role of Sonic hedgehog in normal and abnormal craniofacial morphogenesis. *Development* **126**, 4873–4884 (1999).

116. S. A. Brugmann, N. C. Allen, A. W. James, Z. Mekonnen, E. Madan, J. A. Helms, A primary cilia-dependent etiology for midline facial disorders. *Hum. Mol. Genet.* **19**, 1577–1592 (2010).

117. C. Chiang, Y. Litingtung, E. Lee, K. E. Young, J. L. Corden, H. Westphal, P. A. Beachy, Cyclopia and defective axial patterning in mice lacking Sonic hedgehog gene function. *Nature* **383**, 407–413 (1996).

118. E. Belloni, M. Muenke, E. Roessler, G. Traverso, J. Siegel-Bartelt, A. Frumkin, H. F. Mitchell, H. Donis-Keller, C. Helms, A. V. Hing, H. H. Heng, B. Koop, D. Martindale, J. M. Rommens, L. C. Tsui, S. W. Scherer, Identification of Sonic hedgehog as a candidate gene responsible for holoprosencephaly. *Nat. Genet.* **14**, 353–356 (1996).

119. M. Yamada, S. Mizuno, M. Inaba, T. Uehara, H. Inagaki, H. Suzuki, F. Miya, T. Takenouchi, H. Kurahashi, K. Kosaki, Truncating variants of the sterol recognition region of SHH cause hypertelorism phenotype rather than hypotelorism-holoprosencephaly. *Am. J. Med. Genet. A* **194**, e63614 (2024).

120. M. T. Cobourne, G. M. Xavier, M. Depew, L. Hagan, J. Sealby, Z. Webster, P. T. Sharpe, Sonic hedgehog signalling inhibits palatogenesis and arrests tooth development in a mouse model of the nevoid basal cell carcinoma syndrome. *Dev. Biol.* **331**, 38–49 (2009).

121. X. M. Zhang, M. Ramalho-Santos, A. P. McMahon, Smoothened mutants reveal redundant roles for Shh and Ihh signaling including regulation of L/R asymmetry by the mouse node. *Cell* **105**, 781–792 (2001).

122. J. Boetto, C. Apra, F. Bielle, M. Peyre, M. Kalamarides, Selective vulnerability of the primitive meningeal layer to prenatal Smo activation for skull base meningothelial meningioma formation. *Oncogene* **37**, 4955–4963 (2018).

123. M. E. Swartz, V. Nguyen, N. Q. McCarthy, J. K. Eberhart, Hh signaling regulates patterning and morphogenesis of the pharyngeal arch-derived skeleton. *Dev. Biol.* **369**, 65–75 (2012).

124. M. A. Hoelzl, K. Heby-Henricson, M. Gerling, J. M. Dias, R. V. Kuiper, C. Trünkle, Å. Bergström, J. Ericson, R. Toftgård, S. Teglund, Differential requirement of SUFU in tissue development discovered in a hypomorphic mouse model. *Dev. Biol.* **429**, 132–146 (2017).

125. M. P. Bulman, K. Kusumi, T. M. Frayling, C. McKeown, C. Garrett, E. S. Lander, R. Krumlauf, A. T. Hattersley, S. Ellard, P. D. Turnpenny, Mutations in the human Delta homologue, DLL3, cause axial skeletal defects in spondylocostal dysostosis. *Nat. Genet.* **24**, 438–441 (2000).

126. K. M. Loomes, S. A. Stevens, M. L. O'Brien, D. M. Gonzalez, M. J. Ryan, M. Segalov, N. J. Dormans, M. S. Mimoto, J. D. Gibson, W. Sewell, A. A. Schaffer, H.-D. Nah, E. F. Rappaport, S. C. Pratt, S. L. Dunwoodie, K. Kusumi, Dll3 and Notch1 genetic interactions model axial segmental and craniofacial malformations of human birth defects. *Dev. Dyn.* **236**, 2943–2951 (2007).

127. R. Humphreys, W. Zheng, L. S. Prince, X. Qu, C. Brown, K. Loomes, S. S. Huppert, S. Baldwin, S. Goudy, Cranial neural crest ablation of Jagged1 recapitulates the craniofacial phenotype of Alagille syndrome patients. *Hum. Mol. Genet.* **21**, 1374–1383 (2012).

128. A. W. C. Kung, S.-M. Xiao, S. Cherny, G. H. Y. Li, Y. Gao, G. Tso, K. S. Lau, K. D. K. Luk, J. Liu, B. Cui, M.-J. Zhang, Z. Zhang, J. He, H. Yue, W. Xia, L. Luo, S. He, D. P. Kiel, D. Karasik, Y.-H. Hsu, L. A. Cupples, S. Demissie, U. Styrkarsdottir, B. V. Halldorsson, G. Sigurdsson, U. Thorsteinsdottir, K. Stefansson, J. B. Richards, G. Zhai, N. Soranzo, A. Valdes, T. D. Spector, P. C. Sham, Association of JAG1 with bone mineral density and osteoporotic fractures: A genome-wide association study and follow-up replication studies. *Am. J. Hum. Genet.* **86**, 229–239 (2010).

129. R. Jiang, Y. Lan, H. D. Chapman, C. Shawber, C. R. Norton, D. V. Serreze, G. Weinmaster, T. Gridley, Defects in limb, craniofacial, and thymic development in Jagged2 mutant mice. *Genes Dev.* **12**, 1046–1057 (1998).

130. L. Southgate, M. Sukalo, A. S. V. Karountzos, E. J. Taylor, C. S. Collinson, D. Ruddy, K. M. Snape, B. Dallapiccola, J. L. Tolmie, S. Joss, F. Brancati, M. C. Digilio, L. M. Graul-Neumann, L. Salviati, W. Coerdt, E. Jacquemin, W. Wuyts, M. Zenker, R. D. Machado, R. C. Trembath, Haploinsufficiency of the NOTCH1 receptor as a cause of Adams–Oliver syndrome with variable cardiac anomalies. *Circ. Cardiovasc. Genet.* **8**, 572–581 (2015).

131. M. Abdelkarim, D. Alageel, F. Ahsan, R. Alhuthil, H. Alsarhani, A. Alsagheir, Hajdu-Cheney syndrome with a novel variant in NOTCH2 gene: A case report. *Bone Rep.* **19**, 101709 (2023).

132. E. Canalis, S. Zanotti, Hajdu-Cheney syndrome, a disease associated with NOTCH2 mutations. *Curr. Osteoporos. Rep.* **14**, 126–131 (2016).

133. B. Isidor, P. Lindenbaum, O. Pichon, S. Bézieau, C. Dina, S. Jacquemont, D. Martin-Coignard, C. Thauvin-Robinet, M. Le Merrer, J.-L. Mandel, A. David, L. Faivre, V. Cormier-Daire, R. Redon, C. Le Caignec, Truncating mutations in the last exon of NOTCH2 cause a rare skeletal disorder with osteoporosis. *Nat. Genet.* **43**, 306–308 (2011).

134. R. C. Albertson, T. L. Payne-Ferreira, J. Postlethwait, P. C. Yelick, Zebrafish acvr2a and acvr2b exhibit distinct roles in craniofacial development. *Dev. Dyn.* **233**, 1405–1418 (2005).

135. M. M. Matzuk, T. R. Kumar, A. Bradley, Different phenotypes for mice deficient in either activins or activin receptor type II. *Nature* **374**, 356–360 (1995).

136. M. Dudas, S. Sridurongrit, A. Nagy, K. Okazaki, V. Kaartinen, Craniofacial defects in mice lacking BMP type I receptor Alk2 in neural crest cells. *Mech. Dev.* **121**, 173–182 (2004).

137. D. Rigueur, S. Brugger, T. Anbarchian, J. K. Kim, Y. Lee, K. M. Lyons, The type I BMP receptor ACVR1/ALK2 is required for chondrogenesis during development. *J. Bone Miner. Res.* **30**, 733–741 (2015).

138. Y. Zhou, B. Shu, R. Xie, J. Huang, L. Zheng, X. Zhou, G. Xiao, L. Zhao, D. Chen, Deletion of Axin1 in condylar chondrocytes leads to osteoarthritis-like phenotype in temporomandibular joint via activation of β-catenin and FGF signaling. *J. Cell. Physiol.* **234**, 1720–1729 (2019).

139. H.-M. I. Yu, B. Jerchow, T.-J. Sheu, B. Liu, F. Costantini, J. E. Puzas, W. Birchmeier, W. Hsu, The role of Axin2 in calvarial morphogenesis and craniosynostosis. *Development* **132**, 1995–2005 (2005).

140. M. Bonilla-Claudio, J. Wang, Y. Bai, E. Klysik, J. Selever, J. F. Martin, Bmp signaling regulates a dose-dependent transcriptional program to control facial skeletal development. *Development* **139**, 709–719 (2012).

141. S. Jia, J. Zhou, Y. Gao, J.-A. Baek, J. F. Martin, Y. Lan, R. Jiang, Roles of Bmp4 during tooth morphogenesis and sequential tooth formation. *Development* **140**, 423–432 (2013).

142. J. Gluhak-Heinrich, D. Guo, W. Yang, M. A. Harris, A. Lichtler, B. Kream, J. Zhang, J. Q. Feng, L. C. Smith, P. Dechow, S. E. Harris, New roles and mechanism of action of BMP4 in postnatal tooth cytodifferentiation. *Bone* **46**, 1533–1545 (2010).

143. T. Kouskoura, A. Kozlova, M. Alexiou, S. Blumer, V. Zouvelou, C. Katsaros, M. Chiquet, T. A. Mitsiadis, D. Graf, The etiology of cleft palate formation in BMP7-deficient mice. *PLOS ONE* **8**, e59463 (2013).

144. A. W. Wyatt, R. J. Osborne, H. Stewart, N. K. Ragge, Bone morphogenetic protein 7 (BMP7) mutations are associated with variable ocular, brain, ear, palate, and skeletal anomalies. *Hum. Mutat.* **31**, 781–787 (2010).

145. Z. Malik, D. M. Roth, F. Eaton, J. M. Theodor, D. Graf, Mesenchymal Bmp7 controls onset of tooth mineralization: A novel way to regulate molar cusp shape. *Front. Physiol.* **11**, 698 (2020).

146. Y. Yang, J. Yuan, X. Yao, R. Zhang, H. Yang, R. Zhao, J. Guo, K. Jin, H. Mei, Y. Luo, L. Zhao, M. Tu, Y. Zhu, BMPR1B mutation causes Pierre Robin sequence. *Oncotarget* **8**, 25864–25871 (2017).

147. H. Pan, H. Zhang, P. Abraham, Y. Komatsu, K. Lyons, V. Kaartinen, Y. Mishina, BmpR1A is a major type 1 BMP receptor for BMP-Smad signaling during skull development. *Dev. Biol.* **429**, 260–270 (2017).

148. T. Maruyama, R. Stevens, A. Boka, L. DiRienzo, C. Chang, H.-M. I. Yu, K. Nishimori, C. Morrison, W. Hsu, BMPR1A maintains skeletal stem cell properties in craniofacial development and craniosynostosis. *Sci. Transl. Med.* **13**, eabb4416 (2021).

149. M. Nomura, E. Li, Smad2 role in mesoderm formation, left–right patterning and craniofacial development. *Nature* **393**, 786–790 (1998).

150. M. Mukhopadhyay, S. Shtrom, C. Rodriguez-Esteban, L. Chen, T. Tsukui, L. Gomer, D. W. Dorward, A. Glinka, A. Grinberg, S.-P. Huang, C. Niehrs, J. C. I. Belmonte, H. Westphal, Dickkopf1 is required for embryonic head induction and limb morphogenesis in the mouse. *Dev. Cell* **1**, 423–434 (2001).

151. S. L. Lewis, P. L. Khoo, R. Andrea De Young, H. Bildsoe, M. Wakamiya, R. R. Behringer, M. Mukhopadhyay, H. Westphal, P. P. L. Tam, Genetic interaction of Gsc and Dkk1 in head morphogenesis of the mouse. *Mech. Dev.* **124**, 157–165 (2007).

152. J. White, J. F. Mazzeu, A. Hoischen, S. N. Jhangiani, T. Gambin, M. C. Alcino, S. Penney, J. M. Saraiva, H. Hove, F. Skovby, H. Kayserili, E. Estrella, A. T. Vulto-van Silfhout, M. Steehouwer, D. M. Muzny, V. R. Sutton, R. A. Gibbs, Baylor-Hopkins Center for Mendelian Genomics, J. R. Lupski, H. G. Brunner, B. W. M. van Bon, C. M. B. Carvalho, DVL1 frameshift mutations clustering in the penultimate exon cause autosomal-dominant Robinow syndrome. *Am. J. Hum. Genet.* **96**, 612–622 (2015).

153. S. Beiraghi, V. Leon-Salazar, B. E. Larson, M. T. John, M. L. Cunningham, A. Petryk, J. L. Lohr, Craniofacial and intraoral phenotype of Robinow syndrome forms. *Clin. Genet.* **80**, 15–24 (2011).

154. S. J. Gignac, K. R. MacCharles, K. Fu, K. Bonaparte, G. Akarsu, T. W. Barrett, E. M. Verheyen, J. M. Richman, Mechanistic studies in *Drosophila* and chicken give new insights into functions of DVL1 in dominant Robinow syndrome. *Dis. Model. Mech.* **16**, dmm049844 (2023).

155. S. S. Tophkhane, K. Fu, E. M. Verheyen, J. M. Richman, Craniofacial studies in chicken embryos confirm the pathogenicity of human FZD2 variants associated with Robinow syndrome. *Dis. Model. Mech.* **17**, dmm050584 (2024).

156. C. Zhang, A. Jolly, B. J. Shayota, J. F. Mazzeu, H. Du, M. Dawood, P. C. Soper, A. R. de Lima, B. M. Ferreira, Z. Coban-Akdemir, J. White, D. Shears, F. R. Thomson, S. L. Douglas, A. Wainwright, K. Bailey, P. Wordsworth, M. Oldridge, T. Lester, A. D. Calder, K. Dumic, S. Banka, D. Donnai, S. N. Jhangiani, L. Potocki, W. K. Chung, S. Mora, H. Northrup, M. Ashfaq, J. A. Rosenfeld, K. Mason, L. C. Pollack, A. McConkie-Rosell, W. Kelly, M. McDonald, N. S. Hauser, P. Leahy, C. M. Powell, R. Boy, R. S. Honjo, F. Kok, L. R. Martelli, V. O. Filho, Genomics England Research Consortium, D. M. Muzny, R. A. Gibbs, J. E. Posey, P. Liu, J. R. Lupski, V. R. Sutton, C. M. B. Carvalho, Novel pathogenic variants and quantitative phenotypic analyses of Robinow syndrome: WNT signaling perturbation and phenotypic variability. *HGG Adv.* **3**, 100074 (2022).

157. H. Yu, P. M. Smallwood, Y. Wang, R. Vidaltamayo, R. Reed, J. Nathans, Frizzled 1 and frizzled 2 genes function in palate, ventricular septum and neural tube closure: General implications for tissue fusion processes. *Development* **137**, 3707–3717 (2010).

158. X. Zhu, M. Xu, N. A. Leu, E. E. Morrisey, S. E. Millar, FZD2 regulates limb development by mediating β-catenin-dependent and -independent Wnt signaling pathways. *Dis. Model. Mech.* **16**, dmm049876 (2023).

159. C. van Genderen, R. M. Okamura, I. Fariñas, R. G. Quo, T. G. Parslow, L. Bruhn, R. Grosschedl, Development of several organs that require inductive epithelial-mesenchymal interactions is impaired in LEF-1-deficient mice. *Genes Dev.* **8**, 2691–2703 (1994).

160. T. Sasaki, Y. Ito, X. Xu, J. Han, P. Bringas, T. Maeda, H. C. Slavkin, R. Grosschedl, Y. Chai, LEF1 is a critical epithelial survival factor during tooth morphogenesis. *Dev. Biol.* **278**, 130–143 (2005).

161. W. Dufour, S. Alawbathani, A.-S. Jourdain, M. Asif, G. Baujat, C. Becker, B. Budde, L. Gallacher, T. Georgomanolis, J. Ghoumid, W. Höhne, S. Lyonnet, I. A. Ba-Saddik, S. Manouvrier-Hanu, S. Motameny, A. A. Noegel, L. Pais, C. Vanlerberghe, P. Wagle, S. M. White, M. Willems, P. Nürnberg, F. Escande, F. Petit, M. S. Hussain, Monoallelic and biallelic variants in *LEF1* are associated with a new syndrome combining ectodermal dysplasia and limb malformations caused by altered WNT signaling. *Genet. Med.* **24**, 1708–1721 (2022).

162. N. M. Min Swe, Y. Kobayashi, H. Kamimoto, K. Moriyama, Aberrantly activated Wnt/β-catenin pathway co-receptors LRP5 and LRP6 regulate osteoblast differentiation in the developing coronal sutures of an Apert syndrome ($Fgfr2^{S252W/+}$) mouse model. *Dev. Dyn.* **250**, 465–476 (2021).

163. M. L. Kwee, W. Balemans, E. Cleiren, J. J. Gille, F. Van Der Blij, J. M. Sepers, W. Van Hul, An autosomal dominant high bone mass phenotype in association with craniosynostosis in an extended family is caused by an LRP5 missense mutation. *J. Bone Miner. Res.* **20**, 1254–1260 (2005).

164. H. Turkkahraman, S. Flanagan, T. Zhu, T. M. Bellido, X. Yuan, The LRP5 high-bone-mass mutation causes alveolar bone accrual with minor craniofacial alteration. *J. Periodontal Res.* **58**, 723–732 (2023).

165. P. Kantaputra, P. Jatooratthawichot, K. Chintakanon, W. Intachai, P. Pradermdutsadeeporn, P. Adisornkanj, S. Tongsima, C. Ngamphiw, B. Olsen, A. S. Tucker, J. R. Ketudat Cairns, Mutations in *LRP6* highlight the role of WNT signaling in oral exostoses and dental anomalies. *Arch. Oral Biol.* **142**, 105514 (2022).

166. C. W. Ockeloen, K. D. Khandelwal, K. Dreesen, K. U. Ludwig, R. Sullivan, I. A. L. M. van Rooij, M. Thonissen, S. Swinnen, M. Phan, F. Conte, N. Ishorst, C. Gilissen, L. Roa Fuentes, M. van de Vorst, A. Henkes, M. Steehouwer, E. van Beusekom, M. Bloemen, B. Vankeirsbilck, S. Bergé, G. Hens, J. Schoenaers, V. Vander Poorten, J. Roosenboom, A. Verdonck, K. Devriendt, N. Roeleveldt, S. N. Jhangiani, L. E. L. M. Vissers, J. R. Lupski, J. de Ligt, J. W. Von den Hoff, R. Pfundt, H. G. Brunner, H. Zhou, J. Dixon, E. Mangold, H. van Bokhoven, M. J. Dixon, T. Kleefstra, A. Hoischen, C. E. L. Carels, Novel mutations in LRP6 highlight the role of WNT signaling in tooth agenesis. *Genet. Med.* **18**, 1158–1162 (2016).

167. E. F. Boer, H. F. Van Hollebeke, E. T. Maclary, C. Holt, M. Yandell, M. D. Shapiro, A ROR2 coding variant is associated with craniofacial variation in domestic pigeons. *Curr. Biol.* **31**, 5069–5076.e5 (2021).

168. A. R. Afzal, A. Rajab, C. D. Fenske, M. Oldridge, N. Elanko, E. Ternes-Pereira, B. Tüysüz, V. A. Murday, M. A. Patton, A. O. Wilkie, S. Jeffery, Recessive Robinow syndrome, allelic to dominant brachydactyly type B, is caused by mutation of ROR2. *Nat. Genet.* **25**, 419–422 (2000).

169. G. C. Schwabe, B. Trepczik, K. Süring, N. Brieske, A. S. Tucker, P. T. Sharpe, Y. Minami, S. Mundlos, Ror2 knockout mouse as a model for the developmental pathology of autosomal recessive Robinow syndrome. *Dev. Dyn.* **229**, 400–410 (2004).

170. P. Kantaputra, M. Kaewgahya, D. Jotikasthira, W. Kantaputra, Tricho-odonto-onycho-dermal dysplasia and WNT10A mutations. *Am. J. Med. Genet. A* **164**, 1041–1048 (2014).

171. C. P. Vink, C. W. Ockeloen, S. ten Kate, D. A. Koolen, J. K. Ploos van Amstel, A.-M. Kuijpers-Jagtman, C. C. van Heumen, T. Kleefstra, C. E. L. Carels, Variability in dentofacial phenotypes in four families with WNT10A mutations. *Eur. J. Hum. Genet.* **22**, 1063–1070 (2014).

172. P. Yu, W. Yang, D. Han, X. Wang, S. Guo, J. Li, F. Li, X. Zhang, S.-W. Wong, B. Bai, Y. Liu, J. Du, Z. S. Sun, S. Shi, H. Feng, T. Cai, Mutations in WNT10B are identified in individuals with oligodontia. *Am. J. Hum. Genet.* **99**, 195–201 (2016).

173. K. Yoshinaga, A. Yasue, S. N. Mitsui, Y. Minegishi, S. Oyadomari, I. Imoto, E. Tanaka, Effects of *Wnt10a* and *Wnt10b* double mutations on tooth development. *Genes* **14**, 340 (2023).

174. P. Geetha-Loganathan, S. Nimmagadda, K. Fu, J. M. Richman, Avian facial morphogenesis is regulated by c-Jun N-terminal kinase/planar cell polarity (JNK/PCP) Wingless-related (WNT) signaling. *J. Biol. Chem.* **289**, 24153–24167 (2014).

175. C.-P. Heisenberg, M. Tada, G.-J. Rauch, L. Saúde, M. L. Concha, R. Geisler, D. L. Stemple, J. C. Smith, S. W. Wilson, Silberblick/Wnt11 mediates convergent extension movements during zebrafish gastrulation. *Nature* **405**, 76–81 (2000).

176. L. Slavec, N. Karas Kuželički, I. Locatelli, K. Geršak, Genetic markers for non-syndromic orofacial clefts in populations of European ancestry: A meta-analysis. *Sci. Rep.* **12**, 1214 (2022).

177. M. Vaivads, I. Akota, M. Pilmane, Characterization of SHH, SOX3, WNT3A and WNT9B proteins in human non-syndromic cleft lip and palate tissue. *Dent. J.* **11**, 151 (2023).

178. M. Kaucka, T. Zikmund, M. Tesarova, D. Gyllborg, A. Hellander, J. Jaros, J. Kaiser, J. Petersen, B. Szarowska, P. T. Newton, V. Dyachuk, L. Li, H. Qian, A.-S. Johansson, Y. Mishina, J. D. Currie, E. M. Tanaka, A. Erickson, A. Dudley, H. Brismar, P. Southam, E. Coen, M. Chen, L. S. Weinstein, A. Hampl, E. Arenas, A. S. Chagin, K. Fried, I. Adameyko, Oriented clonal cell dynamics enables accurate growth and shaping of vertebrate cartilage. *eLife* **6**, e25902 (2017).

179. F. He, W. Xiong, X. Yu, R. Espinoza-Lewis, C. Liu, S. Gu, M. Nishita, K. Suzuki, G. Yamada, Y. Minami, Y. Chen, Wnt5a regulates directional cell migration and cell proliferation via Ror2-mediated noncanonical pathway in mammalian palate development. *Development* **135**, 3871–3879 (2008).

180. S. Hosseini-Farahabadi, S. J. Gignac, A. Danescu, K. Fu, J. M. Richman, Abnormal WNT5A signaling causes mandibular hypoplasia in Robinow syndrome. *J. Dent. Res.* **96**, 1265–1272 (2017).

181. T. H. Beaty, J. C. Murray, M. L. Marazita, R. G. Munger, I. Ruczinski, J. B. Hetmanski, K. Y. Liang, T. Wu, T. Murray, M. D. Fallin, R. A. Redett, G. Raymond, H. Schwender, S.-C. Jin, M. E. Cooper, M. Dunnwald, M. A. Mansilla, E. Leslie, S. Bullard, A. C. Lidral, L. M. Moreno, R. Menezes, A. R. Vieira, A. Petrin, A. J. Wilcox, R. T. Lie, E. W. Jabs, Y. H. Wu-Chou, P. K. Chen, H. Wang, X. Ye, S. Huang, V. Yeow, S. S. Chong, S. H. Jee, B. Shi, K. Christensen, M. Melbye, K. F. Doheny, E. W. Pugh, H. Ling, E. E. Castilla, A. E. Czeizel, L. Ma, L. L. Field, L. Brody, F. Pangilinan, J. L. Mills, A. M. Molloy, P. N. Kirke, J. M. Scott, M. Arcos-Burgos, A. F. Scott, A genome-wide association study of cleft lip with and without cleft palate identifies risk variants near MAFB and ABCA4. *Nat. Genet.* **42**, 525–529 (2010).

182. P. N. Kantaputra, S. Kapoor, P. Verma, M. Kaewgahya, K. Kawasaki, A. Ohazama, J. R. Ketudat Cairns, Al-Awadi-Raas-Rothschild syndrome with dental anomalies and a novel WNT7A mutation. *Eur. J. Med. Genet.* **60**, 695–700 (2017).

183. P. N. Kantaputra, S. Mundlos, W. Sripathomsawat, A novel homozygous Arg222Trp missense mutation in WNT7A in two sisters with severe Al-Awadi/Raas-Rothschild/Schinzel phocomelia syndrome. *Am. J. Med. Genet. A* **152A**, 2832–2837 (2010).

184. Y.-R. Jin, X. H. Han, M. M. Taketo, J. K. Yoon, Wnt9b-dependent FGF signaling is crucial for outgrowth of the nasal and maxillary processes during upper jaw and lip development. *Development* **139**, 1821–1830 (2012).

185. K. J. Liu, J. R. Arron, K. Stankunas, G. R. Crabtree, M. T. Longaker, Chemical rescue of cleft palate and midline defects in conditional GSK-3β mice. *Nature* **446**, 79–82 (2007).

186. K. Yu, M. Deng, T. Naluai-Cecchini, I. A. Glass, T. C. Cox, Differences in oral structure and tissue interactions during mouse vs. human palatogenesis: Implications for the translation of findings from mice. *Front. Physiol.* **8**, 154 (2017).

187. I. M. McGonnell, J. D. W. Clarke, C. Tickle, Fate map of the developing chick face: Analysis of expansion of facial primordia and establishment of the primary palate. *Dev. Dyn.* **212**, 102–118 (1998).

188. H. Stainton, M. Towers, Retinoic acid influences the timing and scaling of avian wing development. *Cell Rep.* **38**, 110288 (2022).

189. C.-K. L. Wang, M. Omi, D. Ferrari, H.-C. Cheng, G. Lizarraga, H.-J. Chin, W. B. Upholt, C. N. Dealy, R. A. Kosher, Function of BMPs in the apical ectoderm of the developing mouse limb. *Dev. Biol.* **269**, 109–122 (2004).

190. J. G. Wittig, A. Münsterberg, The chicken as a model organism to study heart development. *Cold Spring Harb. Perspect. Biol.* **12**, a037218 (2020).

191. L. Andrés-Delgado, N. Mercader, Interplay between cardiac function and heart development. *Biochim. Biophys. Acta* **1863**, 1707–1716 (2016).

192. T. Brade, L. S. Pane, A. Moretti, K. R. Chien, K.-L. Laugwitz, Embryonic heart progenitors and cardiogenesis. *Cold Spring Harb. Perspect. Med.* **3**, a013847 (2013).

193. T. P. Kelder, S. N. Duim, R. Vicente-Steijn, A. M. D. Végh, B. P. T. Kruithof, A. M. Smits, T. C. van Bavel, N. A. M. Bax, M. J. Schalij, A. C. Gittenberger-de Groot, M. C. DeRuiter, M.-J. Goumans, M. R. M. Jongbloed, The epicardium as modulator of the cardiac autonomic response during early development. *J. Mol. Cell. Cardiol.* **89**, 251–259 (2015).

194. I. Rzhepakovsky, S. Piskov, S. Avanesyan, M. Shakhbanov, M. Sizonenko, L. Timchenko, A. Nagdalian, M. A. Shariati, A. Al-Farga, F. Aqlan, A. Likhovid, Expanding understanding of chick embryo's nervous system development at HH22-HH41 embryonic stages using X-ray microcomputed tomography. *PLOS ONE* **19**, e0310426 (2024).

195. C. L. Thompson, L. Ng, V. Menon, S. Martinez, C.-K. Lee, K. Glattfelder, S. M. Sunkin, A. Henry, C. Lau, C. Dang, R. Garcia-Lopez, A. Martinez-Ferre, A. Pombero, J. L. R. Rubenstein, W. B. Wakeman, J. Hohmann, N. Dee, A. J. Sodt, R. Young, K. Smith, T.-N. Nguyen, J. Kidney, L. Kuan, A. Jeromin, A. Kaykas, J. Miller, D. Page, G. Orta, A. Bernard, Z. Riley, S. Smith, P. Wohnoutka, M. J. Hawrylycz, L. Puelles, A. R. Jones, A high-resolution spatiotemporal atlas of gene expression of the developing mouse brain. *Neuron* **83**, 309–323 (2014).

196. V. S. Chen, J. P. Morrison, M. F. Southwell, J. F. Foley, B. Bolon, S. A. Elmore, Histology atlas of the developing prenatal and postnatal mouse central nervous system, with emphasis on prenatal days E7.5 to E18.5. *Toxicol. Pathol.* **45**, 705–744 (2017).

197. S. Foppiano, D. Hu, R. S. Marcucio, Signaling by bone morphogenetic proteins directs formation of an ectodermal signaling center that regulates craniofacial development. *Dev. Biol.* **312**, 103–114 (2007).

198. P. Geetha-Loganathan, S. Nimmagadda, L. Antoni, K. Fu, C. J. Whiting, P. Francis-West, J. M. Richman, Expression of WNT signalling pathway genes during chicken craniofacial development. *Dev. Dyn.* **238**, 1150–1165 (2009).

199. P. Murphy, C. Armit, B. Hill, S. Venkataraman, P. Frankel, R. A. Baldock, D. R. Davidson, Integrated analysis of Wnt signalling system component gene expression. *Development* **149**, dev200312 (2022).
